# Leveraging the Aminothiol-Specific Phosphorogenic Response of Iridium(III) Thioester Complexes for the Development of Intracellular Sensors and Cancer Phototherapeutics

**DOI:** 10.1021/jacsau.5c00413

**Published:** 2025-06-10

**Authors:** Eunice Chiu-Lam Mak, Ziyong Chen, Lawrence Cho-Cheung Lee, Liang-Liang Yan, Vivian Wing-Wah Yam, Kenneth Kam-Wing Lo

**Affiliations:** † Department of Chemistry, 53025 City University of Hong Kong, Kowloon, Hong Kong, P. R. China; ‡ Institute of Molecular Functional Materials and Department of Chemistry, 25809 The University of Hong Kong, Pokfulam Road, Hong Kong, P. R. China; & State Key Laboratory of Structural Chemistry, 58281 Fujian Institute of Research on the Structure of Matter, Chinese Academy of Sciences, Fuzhou 350002, P. R. China; § State Key Laboratory of Terahertz and Millimeter Waves, 53025 City University of Hong Kong, Kowloon, Hong Kong, P. R. China

**Keywords:** bioconjugation, bioimaging, intracellular sensing, iridium, N-terminal cysteine, phosphorogenic, photosensitizers, thioester

## Abstract

Site-specific bioconjugation techniques are extensively utilized in biological and biomedical fields to precisely label biomolecules with luminescent tags for direct visualization of their intracellular dynamics or with cytotoxic agents for the development of novel anticancer therapeutics. In this work, a series of cyclometalated iridium­(III) polypyridine complexes featuring a thioester moiety was designed as novel phosphorogenic probes for labeling N-terminal cysteine (N-Cys)-containing biomolecules. These thioester complexes were weakly emissive in solutions due to the presence of a low-lying nonradiative distorted triplet intraligand (^3^IL) state localized on the thioester unit, as elucidated by computational analyses. However, their emission intensities and singlet oxygen (^1^O_2_)-photosensitization efficiencies substantially increased upon reaction with l-Cys due to the conversion of the quenching thioester moiety to a nonquenching amide unit. Additionally, the thioester complexes exhibited high selectivity toward N-Cys and displayed significantly enhanced reactivity due to the electron-withdrawing iridium­(III) polypyridine moiety. The remarkable aminothiol-induced emission and ^1^O_2_-photosensitization turn-on of the thioester complexes were exploited for the development of intracellular Cys sensors and Cys-activatable photosensitizers for cancer-targeted photodynamic therapy. Furthermore, one of the thioester complexes was selected to react with various N-Cys-modified tumor-targeting peptides, yielding photofunctional iridium­(III)–peptide conjugates with high ^1^O_2_ generation efficiencies. These conjugates retained the tumor-targeting capabilities of the original peptides and showed high specificity for MDA-MB-231 cells compared to MCF-7 and HEK-293 cells, resulting in selective photocytotoxicity toward this triple-negative breast cancer cell line. We believe that our design approach will inspire the development of novel luminogenic thioester-based reagents for bioconjugation, bioimaging, and therapeutic applications.

## Graphical abstract

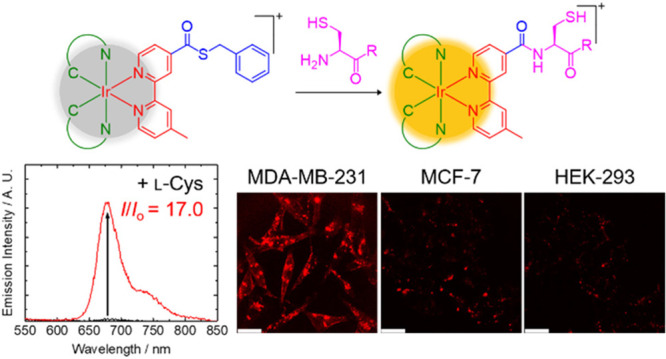

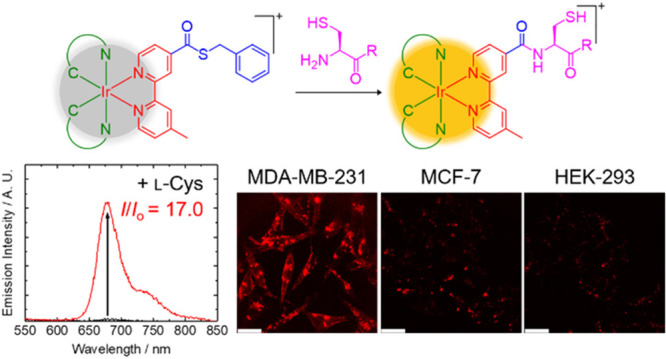

## Introduction

Bioconjugation holds immense importance in the fields of biochemistry and biomedicine.^1^ This process entails the covalent attachment of biomolecules to generate compounds with augmented functionalities. Its versatility is demonstrated across a wide range of applications, such as site-specific labeling of biomolecules for bioimaging and biosensing,^2^ targeted drug delivery,^3^ and immunoassays.^4^ However, traditional bioconjugation techniques, such as those involving the modification of lysine (Lys) and cysteine (Cys) residues, are constrained by drawbacks including the heterogeneous labeling of biomolecules.^5^ This complexity complicates purification and has adverse effects on the biophysical and pharmacokinetic properties of the conjugates. Other biocompatible reactions, such as the Staudinger ligation,^6^ ketone–hydrazide reaction,^7^ and numerous bioorthogonal reactions like the inverse electron-demand Diels–Alder cycloaddition,^8^ necessitate the integration of unnatural functional groups into biomolecules or intricate genetic manipulations. N-Terminal cysteine (N-Cys) residues have been employed for site-specific labeling of peptides and proteins due to several key factors.^9^ These include the unique reactivity of the 1,2-aminothiol group and the low natural abundance of N-Cys residues, enabling the selective labeling of N-Cys over internal residues. Additionally, the modification of N-Cys residues causes minimal structural perturbation, thereby preserving the native conformation of the biomolecule. Thioesters have conventionally been utilized in ligation reactions with N-Cys residues of unprotected peptides via native chemical ligation (NCL) under physiological conditions for protein synthesis.^10,11^ The process involves transthioesterification followed by a spontaneous *S* → *N* acyl shift to yield a stable amide bond. Additionally, thioester-functionalized fluorophores have been developed as NCL-based probes for detecting biothiols^12–20^ and labeling peptides and proteins.^21–24^

Luminescent transition metal complexes have been pivotal in the realm of biological research due to their remarkable photophysical and biological properties.^25–32^ Our group has a long-standing interest in the development of transition metal complexes as bioconjugation reagents,^33^ particularly in the preparation of photofunctional bioconjugates for bioimaging and targeted photodynamic therapy (PDT) applications.^34–38^ Recently, we have developed iridium­(III) 2-formylphenylboronic acid^37^ and 2-cyanobenzothiazole^38^ complexes that enable selective modification of N-Cys-containing biomolecules. However, these complexes lack the capability to display phosphorogenic response (i.e., phosphorescence turn-on) upon the conjugation reaction, hindering their potential applications in imaging N-Cys-containing biomolecules in the cellular environment with high sensitivity. Given that thioesters exhibit distinctive emission quenching capabilities in thioester-modified stilbenes,^39–41^ we postulate that the incorporation of a thioester moiety into transition metal complexes will generate a unique class of phosphorogenic bioconjugation reagents for labeling N-Cys-containing biomolecules, providing a new platform for bioimaging and phototherapeutic applications. In this work, we designed and synthesized a series of cyclometalated iridium­(III) polypyridine complexes featuring a thioester group [Ir­(N^C)_2_(bpy-COSBn)]­(PF_6_) (bpy-COSBn = *S*-benzyl 4’-methyl-2,2’-bipyridine-4-carbothioate; HN^C = 2-phenylquinoline (Hpq) (**1a**), 2-(1-naphthyl)­benzothiazole (Hbsn) (**2a**), 1-(benzo­[*b*]-thiophen-2-yl)­isoquinoline (Hiqbt) (**3a**), and 6-(benzo­[*b*]­thiophen-2-yl)­phenanthridine (Hbtph) (**4a**)) (Chart 1). Their ester counterparts [Ir­(N^C)_2_(bpy-COOMe)]­(PF_6_) (bpy-COOMe = methyl 4’-methyl-2,2’-bipyridine-4-carboxylate; HN^C = Hpq (**1b**), Hbsn (**2b**), Hiqbt (**3b**), and Hbtph (**4b**)) were also prepared for comparison studies. The photophysical, photochemical, and electrochemical properties of the complexes were studied. Additionally, the reactivity, selectivity, and possible phosphorogenic response of the thioester complexes toward l-Cys were examined. The utilization of the thioester complexes for intracellular Cys sensing and cancer-targeted PDT was also investigated. Furthermore, various tumor-targeting peptide conjugates were constructed from the reaction of the thioester complex and N-Cys-modified peptides, and their photophysical and photochemical properties, cellular uptake, intracellular localization, and (photo)­cytotoxicity were studied.

**Chart 1:**
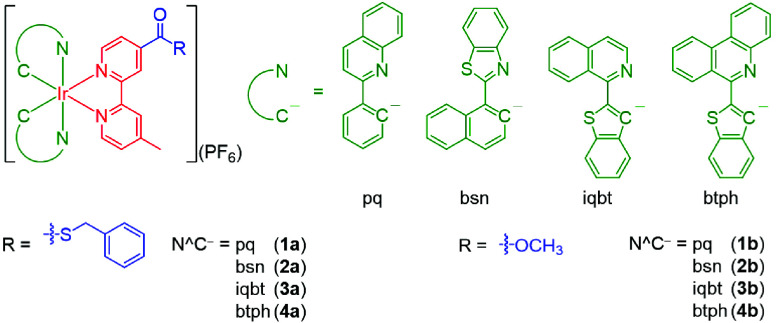
Structures of the Iridium­(III) Thioester Complexes **1a**–**4a** and Ester Complexes **1b**–**4b**

## Results and Discussion

### Synthesis and Characterization of the Iridium­(III) Complexes

The synthesis of the thioester ligand bpy-COSBn involved the reaction of 4-succinimidylcarboxy-4’-methyl-2,2’-bipyridine (bpy-NHS) with benzyl mercaptan in an anhydrous THF solution containing *N*,*N*-diisopropylethylamine (DIPEA) and 4-dimethylaminopyridine (DMAP). The synthesis of the ester ligand bpy-COOMe was performed following procedures in the literature.^42^ The iridium­(III) complexes were prepared by the reaction of iridium­(III) dimers [Ir_2_(N^C)_4_Cl_2_] (HN^C = Hpq, Hbsn, Hiqbt, and Hbtph) with bpy-COSBn or bpy-COOMe in CH_2_Cl_2_/MeOH, followed by anion exchange with KPF_6_, and purification by column chromatography and recrystallization from CH_2_Cl_2_/Et_2_O to afford orange to deep red crystals. The complexes were characterized by HR-ESI-MS, ^1^H and ^13^C NMR, and IR spectroscopy, and gave satisfactory elemental analyses. Single crystals of complex **1a** were obtained by vapor diffusion of Et_2_O into a concentrated solution of the complex in CH_3_CN. Crystallographic data, selected bond lengths, and bond angles are listed in Tables S1 and S2. The perspective view of the complex cation is shown in Figure 1.

**Figure 1:**
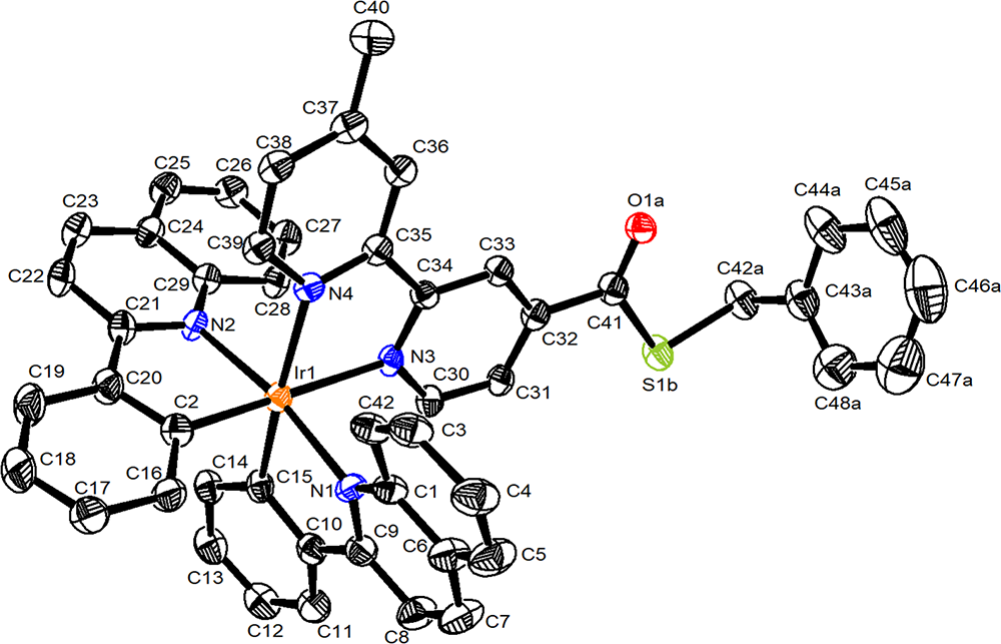
Perspective view of the cation of complex **1a**, [Ir­(pq)_2_(bpy-COSBn)]^+^. Thermal ellipsoids are shown at the 30% probability level. Hydrogen atoms are omitted for clarity.

The iridium­(III) center of the complex adopts a distorted octahedral geometry, and the *trans* angles at the metal center range from 170.7 to 172.1°. The Ir–C bonds of the cyclometalating ligands are coordinated to the metal center in a *cis* orientation. The *trans* influence of the carbon donors renders slightly longer Ir–N bond lengths for the bpy-COSBn ligand (2.169 Å and 2.179 Å) than those for the pq ligands (2.094 and 2.119 Å). The bite angles of the pq ligands (79.2 and 79.5°) are larger than that of the bpy-COSBn ligand (75.06°), which is similar to those of related cyclometalated iridium­(III) polypyridine systems, [Ir­(N^C)_2_(N^N)]^+^.^43–48^

### Photophysical, Photochemical, and Electrochemical Properties

The electronic absorption spectra and data of the thioester complexes **1a**–**4a** and ester complexes **1b**–**4b** are presented in Figures S1 and S2 and Table S3, respectively. All the complexes displayed intense spin-allowed intraligand (^1^IL) (π → π*) (N^N and N^C) absorption bands at ca. 250–350 nm and weaker spin-allowed metal-to-ligand charge-transfer (^1^MLCT) (dπ­(Ir) → π*­(N^N and N^C)) absorption bands/shoulders at ca. 360–550 nm.^49–52^ The weak absorption tail beyond ca. 560 nm is assigned to spin-forbidden ^3^MLCT (dπ­(Ir) → π*­(N^N and N^C)) transitions. Upon photoexcitation, all the complexes exhibited orange-red to near-infrared (NIR) emission in solutions under ambient conditions and in low-temperature alcohol glass. The emission spectra and photophysical data of the thioester and ester complexes are presented in Figures S3 and S4 and Table S4, respectively. Importantly, the thioester complexes **1a**–**4a** (Φ_em_ = 0.002–0.025 in CH_3_CN) exhibited significantly lower emission quantum yields than their ester counterparts **1b**–**4b** (Φ_em_ = 0.010–0.13 in CH_3_CN), indicative of emission quenching associated with the thioester moiety. The pq complexes (**1a**,**b**) displayed positive solvatochromism and short emission lifetimes in fluid solutions at 298 K and a significant blue shift upon cooling the samples to 77 K, suggestive of a predominant ^3^MLCT (dπ­(Ir) → π*­(N^N)) emissive state.^49^ However, there should be mixing of some ^3^IL (π → π*) (pq) character due to their structured emission bands and long emission lifetimes (5.07 and 4.62 μs) in 77-K glass. In contrast, the bsn (**2a**,**b**), iqbt (**3a**,**b**), and btph (**4a**,**b**) complexes showed a structured NIR emission band with low solvent dependency in fluid solutions at 298 K and long emission lifetimes (2.88–5.49 μs) in 77-K glass, suggestive of a predominant ^3^IL (π → π*) (N^C) excited state.^50–52^

The ^1^O_2_ generation efficiencies of all complexes were evaluated by monitoring the emission band of ^1^O_2_ centered at ca. 1270 nm^53,54^ in aerated CH_3_CN (Table S5). The thioester complexes **1a**–**4a** showed lower ^1^O_2_ generation quantum yields (0.13–0.80) than the ester complexes **1b**–**4b** (0.51–0.98), indicating quenching of the complexes by the thioester group. Among the ester complexes **1b**–**4b**, the bsn (**2b**), iqbt (**3b**), and btph (**4b**) complexes displayed substantially higher ^1^O_2_ generation efficiencies (Φ_Δ_ = 0.82–0.98) than the pq complex (**1b**) (Φ_Δ_ = 0.51), primarily due to the presence of a low-lying, long-lived ^3^IL excited state (τ_o_ = 1.28–3.79 μs; Table S4) for ^1^O_2_ photosensitization.

The electrochemical properties of the thioester complexes **1a**–**4a** were studied by cyclic voltammetry, and the electrochemical data are listed in Table S6. These complexes exhibited a quasi-reversible oxidation couple at +1.12 to +1.38 V versus SCE, which is assigned to a metal-centered iridium­(IV)/(III) oxidation process.^43,50^ Based on the first reduction potentials (−0.92 to −0.99 V versus SCE, Table S6) and the low-temperature emission energy (*E*_00_ = 1.79–2.28 eV, Table S4) of the thioester complexes, the excited-state redox potentials (*E*°[Ir^2+/+^*]) of complexes **1a**–**4a** were determined to range from −0.63 to −0.96 V versus SCE (Figure S5). These potentials are less negative than the reduction potential of bpy-COSBn (−1.00 V versus SCE; Table S6), suggesting that the redox reaction between the excited iridium­(III) complexes and the appended thioester moiety is not thermodynamically favorable (Δ*G*° = +0.05 to +0.37 eV). This eliminates the possibility of photoinduced electron transfer (PeT) as the quenching mechanism.

### Reactivity, Selectivity, and Phosphorogenic Response toward N-Cys

The reactivity of the thioester complexes **1a**–**4a** toward N-Cys-containing biomolecules in potassium phosphate buffer (50 mM, pH 7.0)/DMSO (3:2, *v*/*v*) containing tris­(2-carboxyethyl)­phosphine (TCEP) (250 μM) at 298 K (Scheme 1) were investigated using l-Cys as a model. As revealed by high-performance liquid chromatography (HPLC) analyses, the reaction of the thioester complexes (20 μM) with l-Cys (25 μM) completed within 1 h, with conversion yields exceeding 95% (Figure S6). Using complex **1a** as an example, the initial peak at *t*_R_ = 9.7 min disappeared, and a new peak at *t*_R_ = 6.8 min emerged in the chromatogram after incubation with l-Cys for 1 h (Figure S6). The formation of the conjugation products **1a-Cys**–**4a-Cys** was validated by ESI-MS analyses (Figure S7). The isolated conjugate **1a-Cys** was further characterized by 1D and 2D COSY ^1^H NMR. The signals at δ = 8.18 and 1.89 ppm in the ^1^H NMR spectra (Figures S8 and S9) correspond to the CONH and SH protons, respectively, confirming the successful labeling of l-Cys with the thioester complex via the NCL reaction involving transthioesterification and an *S* → *N* acyl shift. The reaction kinetics of complexes **1a**–**4a** with l-Cys was studied in buffer solutions at 298 K by monitoring the reaction at different time intervals using HPLC. The second-order rate constants (*k*_2_) for the reactions range from 189.2 to 2,385.5 M^–1^ s^–1^, following the order: **4a** < **2a** < **3a** < **1a** (Figure S10). These values are one to two orders of magnitude higher than that of the ligand bpy-COSBn (11.3 M^–1^ s^–1^), illustrating that the direct conjugation of the thioester moiety to the cationic iridium­(III) polypyridine unit significantly enhances the reactivity.^47,48,55–58^ In particular, complexes **1a** and **3a** demonstrated remarkable reactivity at a rate of 10^3^ M^–1^ s^–1^, which facilitates the rapid labeling of N-Cys-containing biomolecules. To evaluate the selectivity and stability of the thioester complexes, complex **1a** was selected as a model and the reactions were monitored by HPLC and ESI-MS. The complex remained intact and showed negligible reaction upon incubation with a large excess of amino acids, including l-Lys, l-histidine (l-His), l-serine (l-Ser), and l-threonine (l-Thr) (2 mM) (Figure S11). Importantly, upon incubation with peptide models containing a Cys at the N-terminus (CSS), center (SCS), and C-terminus (SSC), complex **1a** selectively reacted with CSS (Figure 2a), forming the conjugate **1a-CSS** (*t*_R_ = 5.8 min) as evidenced by ESI-MS analysis (Figure 2b). These results highlight the high reactivity and excellent chemo- and regioselectivity of the thioester complexes toward N-Cys-containing biomolecules.

**Scheme 1:**
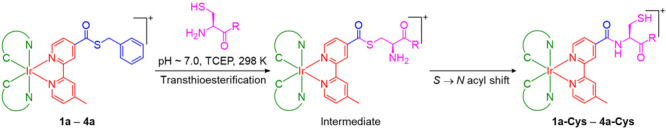
Conjugation of the Thioester Complexes to N-Cys-Containing Biomolecules via NCL

**Figure 2:**
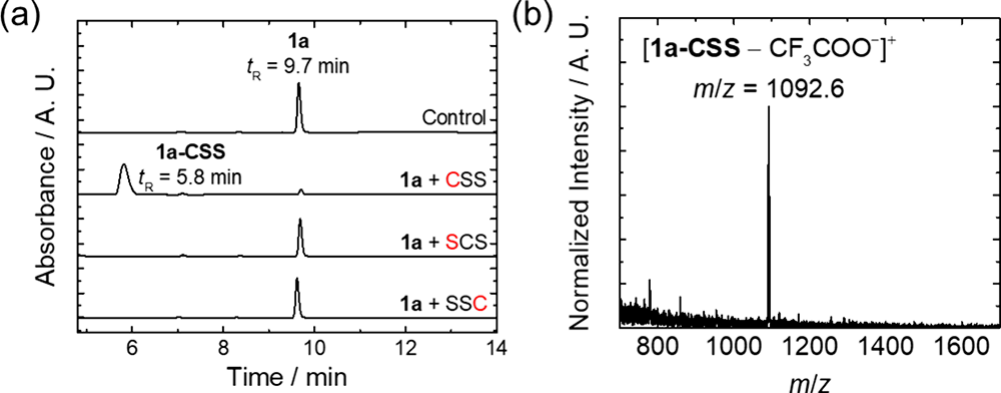
(a) HPLC chromatograms of the reaction mixtures of complex **1a** (25 μM) without (control) and with CSS (1 mM), SCS (1 mM), and SSC (1 mM) in potassium phosphate buffer (50 mM, pH 7.0)/DMSO (3:2, *v*/*v*) containing TCEP (10 mM) after incubation at 298 K for 1 h. The absorbance was monitored at 350 nm. (b) ESI mass spectrum of the eluent collected at *t*_R_ = 5.8 min of the reaction of complex **1a** and CSS.

Notably, upon incubation of the thioester complexes (10 μM) with l-Cys (100 μM) in TCEP-containing buffer solutions at 298 K for 1 h, substantial emission enhancement was observed in the solutions (*I*/*I*_o_ = 10.7–31.8) (Table S7 and Figure 3) due to conversion of the quenching thioester moiety into a nonquenching amide group during the NCL reaction. Interestingly, complex **1a** displayed a bathochromic shift in its emission maximum from ca. 556 to 606 nm upon reaction with l-Cys (Figure 3, Table S7, and Figure S12). The distinct photophysical changes of complex **1a** upon the NCL reaction compared to complexes **2a**–**4a** can be attributed to the larger involvement of ^3^MLCT (dπ­(Ir) → π*­(N^N)) character in its emissive state, which renders it more sensitive to the structural changes on the bpy ligand (i.e., from thioester to amide) associated with the NCL reaction. The observed photophysical changes brought about by the NCL reaction highlight the potential applications of the thioester complexes not only for precise labeling of biomolecules to yield photofunctional conjugates, but also for imaging l-Cys and N-Cys-containing biomolecules in live cells to examine their functions and dynamics.

**Figure 3:**
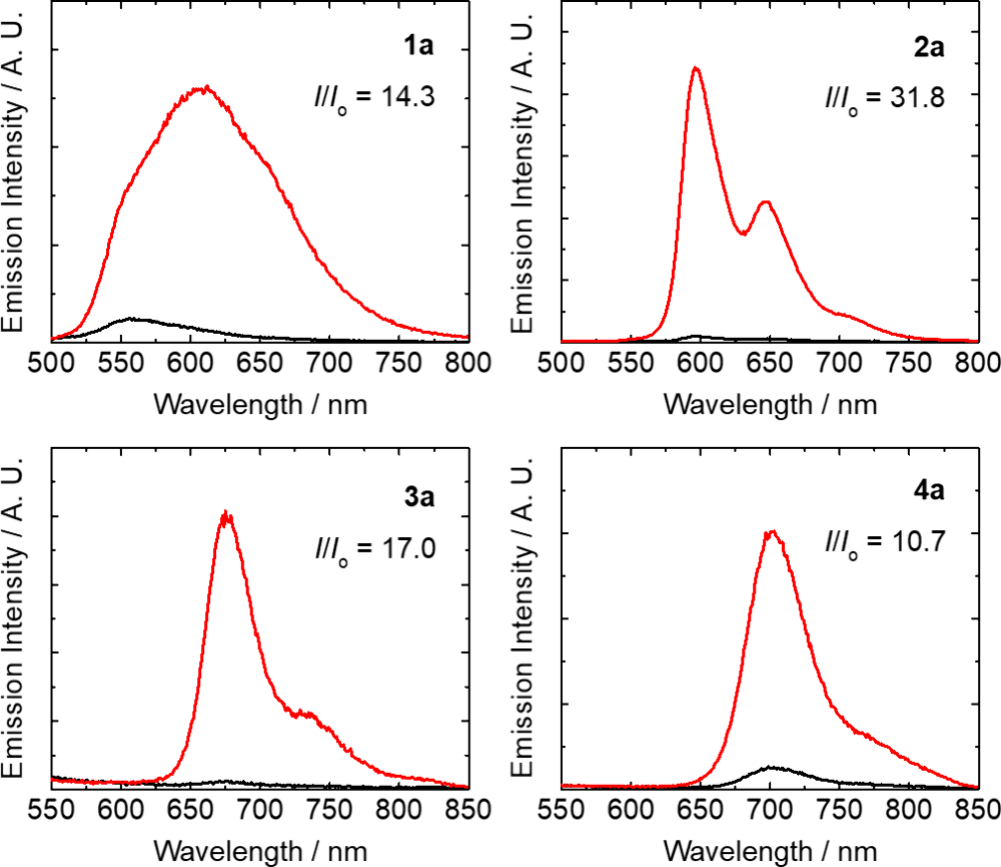
Emission spectra of complexes **1a**–**4a** (10 μM) before (black) and after (red) incubation with l-Cys (100 μM) in aerated potassium phosphate buffer (50 mM, pH 7.0)/CH_3_CN (3:2, *v*/*v*) containing TCEP (1 mM) at 298 K for 1 h.

To determine the effect of bioconjugation on the photophysical and photochemical properties of the complexes, their Cys conjugates **1a-Cys**–**4a-Cys** were isolated and purified by semipreparative HPLC. All Cys conjugates exhibited high emission quantum yields (Φ_em_ = 0.016–0.12) (Table S8 and Figure S13) and ^1^O_2_ generation quantum yields (Φ_Δ_ = 0.53–0.98) (Table S9) in CH_3_CN, comparable to the ester complexes (Φ_em_ = 0.010–0.13, Φ_Δ_ = 0.51–0.98; Tables S4 and S5). Importantly, complex **3a** displayed the most pronounced change in the ^1^O_2_ generation quantum yield after reacting with l-Cys (from 0.13 to 0.92; Tables S5 and S9), showcasing the controllable ^1^O_2_-photosensitization behavior of the thioester complexes via the NCL reaction.

### Computational Studies

To gain more insights into the emission enhancement of thioester complexes upon reaction with l-Cys, density functional theory (DFT) and unrestricted density functional theory (UDFT) calculations were performed on the thioester complex **1a-Me** (an *S*-methyl thioester analogue of complex **1a**), its NCL reaction product **1a-Cys**, and ester counterpart **1b**. The benzyl group in the thioester moiety of complex **1a** is simplified to a methyl group in **1a-Me** for the comparison with the methyl ester complex **1b**. For all three complexes, the emissive triplet (T_1_) state is dominated by the ^3^MLCT (dπ­(Ir) → π*­(bpy-COSMe), dπ­(Ir) → π*­(bpy-CONH-Cys), and dπ­(Ir) → π*­(bpy-COOMe) for complexes **1a-Me**, **1a-Cys**, and **1b**, respectively) character (Figure 4). The emission energy of complex **1a-Me** computed at the relaxed ^3^MLCT structure is 1.82 eV (Table S10), which is in good agreement with the experimental emission measured in CH_2_Cl_2_ (λ_em_ = 648 nm, 1.91 eV; Table S4). The emission bands are computed to be 1.96 and 1.92 eV for complexes **1a-Cys** and **1b**, respectively, which are also in line with the experimentally observed blue shift in λ_em_. It should be noted that the distortion of the bpy-based ligands can lead to triplet distorted states (^3^DS) that deactivate the radiative ^3^MLCT → S_0_ process.^59,60^ A metal-centered (^3^MC) state, featuring a five-coordinate iridium center through the dissociation of an Ir–N bond, is energetically higher-lying than the ground (S_0_) state by ca. 2.8 eV (Figure 4). This state has been identified for all three complexes, and might not be directly related to the emission enhancement upon conversion of complex **1a-Me** into **1a-Cys**. Interestingly, in contrast to the amide and ester groups in complexes **1a-Cys** and **1b**, respectively, the planar thioester moiety (θ­(O=C–S–C) = 0.0°) in complex **1a-Me** can undergo a structural distortion to form a ^3^IL state with an O=C–S–C torsion angle of −86.6°. As compared to the large vertical T_1_–S_0_ gap (1.82 eV) at the relaxed ^3^MLCT structure, the T_1_–S_0_ gap at the relaxed ^3^IL structure is decreased to 1.24 eV, which might facilitate nonradiative decay through the ^3^IL → S_0_ intersystem crossing (ISC) process. Based on Marcus theory (Table S11), the rate constant (*k*_ISC_) of the ^3^IL → S_0_ ISC is computed to be 1.65 × 10^8^ s^–1^, which is several orders of magnitude greater than the rate constant for the radiative decay process (*k*_r_^P^ = 3.41 × 10^2^ s^–1^). Thus, the distorted ^3^IL state localized on the bpy-COSMe ligand is suggested to be nonemissive. From our computational interpretations, we believe that energy transfer from the emissive ^3^MLCT state to the nonemissive ^3^IL (thioester) state leads to the emission quenching observed in the iridium­(III) thioester complexes.

**Figure 4:**
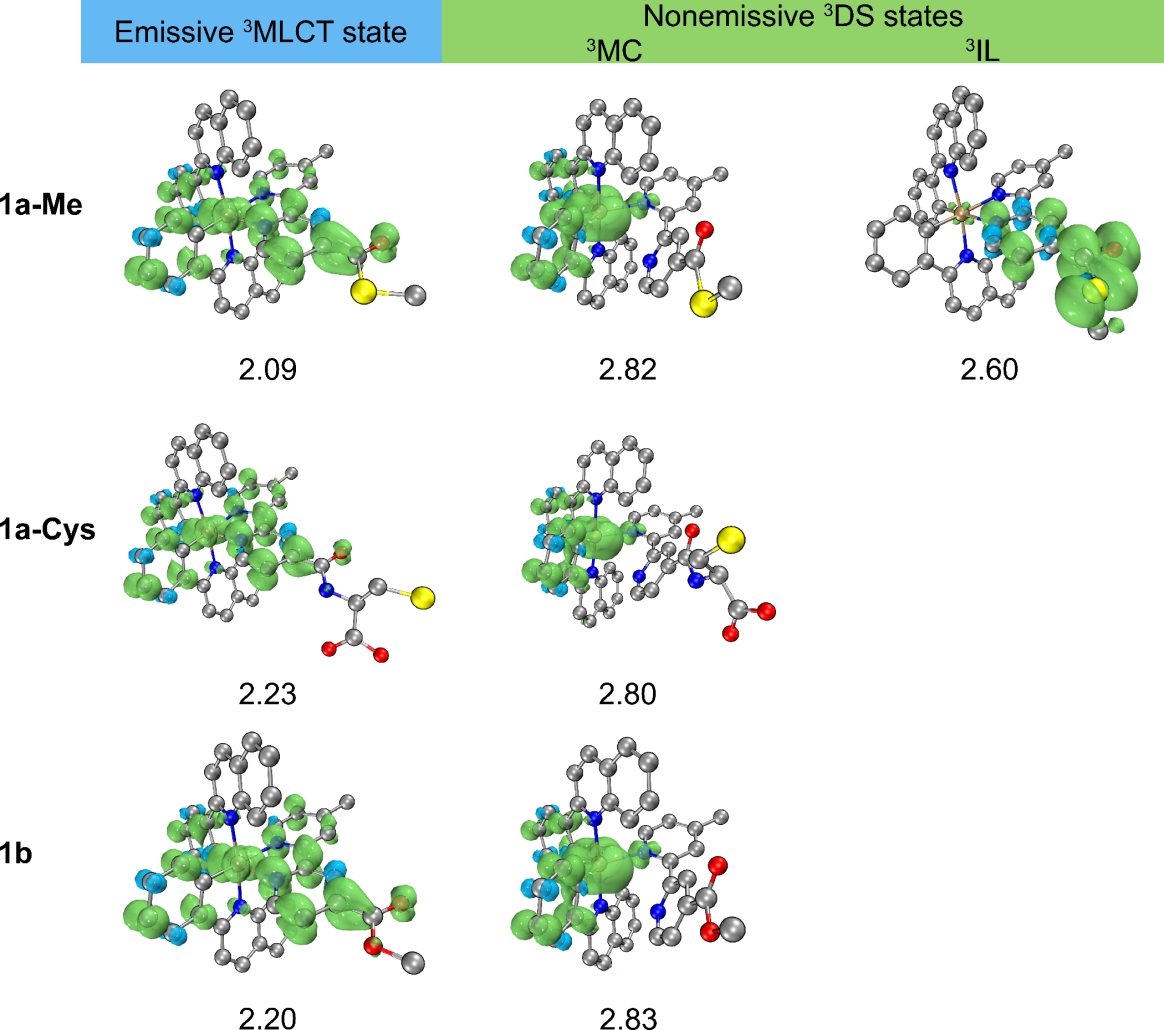
Spin densities of the emissive ^3^MLCT state and nonemissive ^3^DS states at their optimized structures for complexes **1a-Me**, **1a-Cys**, and **1b**. Computed energy levels (eV) with respect to the optimized S_0_ state are provided.

### Intracellular Cysteine Sensing

The intriguing aminothiol-specific phosphorogenic response of the thioester complexes motivated us to explore their applications in sensing intracellular Cys and N-Cys-containing proteins. Complex **1a** was used as a model compound due to (1) its high reactivity (*k*_2_ = 2,385.5 M^–1^ s^–1^; Figure S10); (2) distinctive emission profiles before and after the NCL reaction with Cys (Figure 3); and (3) high selectivity toward Cys over other thiols such as ethanethiol and glutathione (GSH) (the most abundant biothiol in cells) (Table S12 and Figure S14). Laser-scanning confocal microscopy (LSCM) images of live HeLa cells incubated with complex **1a** (10 μM, 1 h) revealed intense cytoplasmic emission (Figure 5), which is due to the reaction of the complex with intracellular Cys to give the emissive amide product **1a-Cys**.^61^ Additionally, the intracellular emission spectrum of cells treated with complex **1a** closely resembles that of cells treated with conjugate **1a-Cys** (Figure S16), confirming the reaction of complex **1a** with intracellular Cys. However, when the cells were pretreated with the thiol scavenger *N*-ethylmaleimide (NEM) (100 μM, 20 min), the emission intensity significantly decreased, which is attributed to the reduced intracellular Cys level. The emission was restored upon further incubation of the NEM-pretreated cells with l-Cys (100 μM, 30 min), supporting that the observed intracellular emission was due to the specific reaction of the thioester complex with Cys. These results demonstrate that complex **1a** can function as a sensor for intracellular Cys. This also suggests the potential for detecting N-Cys-containing proteins in live cells.^62^

**Figure 5:**
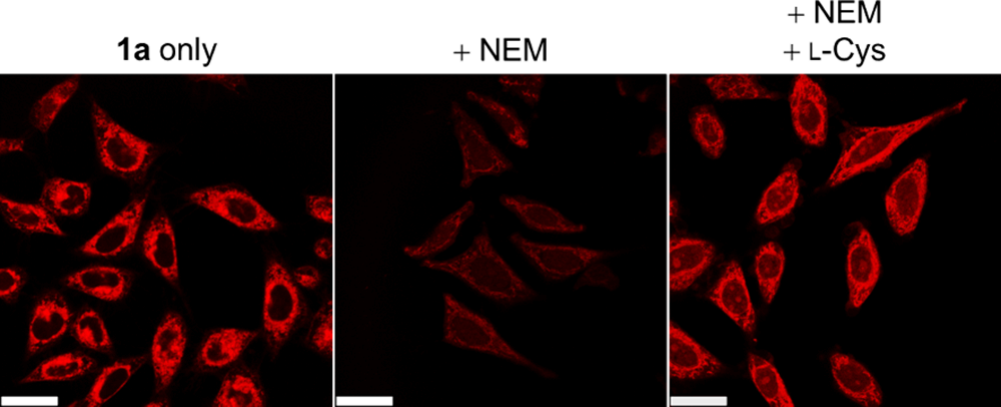
LSCM images of live HeLa cells incubated with complex **1a** (10 μM, 1 h, λ_ex_ = 405 nm, λ_em_ = 560–660 nm) without or with pretreatment of NEM (100 μM, 20 min) or with pretreatment of NEM (100 μM, 20 min) and l-Cys (100 μM, 30 min). Scale bars = 25 μm.

### Cancer-Targeted PDT

Despite its role as an essential biothiol in maintaining intracellular redox homeostasis,^63^ Cys is known as an important cancer biomarker due to its overexpression in cancer tissues compared to normal tissues.^64^ Given the significant increase in the ^1^O_2_ generation efficiency of complex **3a** upon reaction with l-Cys (from 0.13 to 0.92 in CH_3_CN; Tables S5 and S9), we anticipate that complex **3a** can serve as a Cys-activatable photosensitizer for cancer-targeted PDT. Thus, we examined the (photo)­cytotoxic activity of complex **3a** in cancerous MDA-MB-231 (high intracellular Cys level) and normal HEK-293 (low intracellular Cys level) cells using the 3-(4,5-dimethylthiazol-2-yl)-2,5-diphenyltetrazolium bromide (MTT) assay. The complex was essentially noncytotoxic in the dark (IC_50,dark_ > 50 μM) (Table S13) in both cell lines. However, upon irradiation at 450 nm (light dosage = 14.6 mW cm^–2^) for 10 min, the complex exhibited remarkable photocytotoxic effects, with greater potency in MDA-MB-231 cells (IC_50,light_ = 0.57 μM) compared to HEK-293 cells (IC_50,light_ = 1.7 μM) (Table S13). This discrepancy in potency can be ascribed to (1) the higher cellular uptake of the complex in MDA-MB-231 cells (0.18 fmol/cell) than HEK-293 cells (0.087 fmol/cell) (Table S13); and (2) the higher intracellular Cys level in MDA-MB-231 cells leading to the greater *in situ* formation of conjugate **3a-Cys**. These results highlight the potential of complex **3a** in cancer-selective PDT.

### Preparation and Characterization of Tumor-Targeting Iridium­(III)–Peptide Conjugates

Considering that the thioester complex **3a** showed rapid reaction kinetics toward l-Cys (*k*_2_ = 1,658.3 M^–1^ s^–1^; Figure S10) and its Cys conjugate **3a-Cys** showed a high ^1^O_2_ generation quantum yield (Φ_Δ_ = 0.92 in CH_3_CN; Table S9), complex **3a** was used to modify various tumor-targeting peptides to yield photofunctional metal–peptide conjugates for theranostic applications. Three peptides were selected: (1) CASPSGALRSC (CASP), which demonstrates high targeting specificity toward the triple-negative breast cancer MDA-MB-231 cell line;^65^ (2) YNTNHVPLSPKY (YNT), which targets the enzyme carbonic anhydrase IX (CAIX) that is often overexpressed in various cancer cell types under hypoxic conditions;^66^ and (3) CMYIEALDKYAC (CMYI), which targets the epidermal growth factor receptor (EGFR), a critical transmembrane receptor that is overexpressed in numerous cancer cell lines.^67^ All three peptides were modified with an N-Cys residue for conjugation. The iridium­(III)–peptide conjugates **3a-CASP**, **3a-CYNT**, and **3a-CMYI** were prepared by incubation of complex **3a** with the peptides CASP, CYNT, and CMYI, respectively, in TCEP-containing buffer solutions at 298 K for 12 h. Notably, despite the presence of an intrinsic Cys residue at the C-terminus of both CASP and CMYI peptides, the reactions only yielded one major product (Figure S17), highlighting the excellent chemo- and regioselectivity of the thioester complexes. The conjugates were purified by semipreparative HPLC, and the purified products were characterized by HPLC and ESI-MS analyses (Figures S18 and S19). Upon photoexcitation, the conjugates displayed moderate emission intensities and lifetimes in aqueous solutions (Table S8 and Figure S20). Additionally, the peptide conjugates exhibited comparable ^1^O_2_ photosensitization capabilities (Φ_Δ_ = 0.20–0.28) to conjugate **3a-Cys** (Φ_Δ_ = 0.26) in aqueous solutions (Table S14). The enhanced emission quantum yields, extended lifetimes, and increased ^1^O_2_ photosensitization efficiencies of the conjugates, as compared to the thioester complex **3a**, indicate the elimination of the thioester-mediated quenching pathway upon the NCL reaction.

### Cellular Uptake, Localization, and (Photo)­cytotoxicity of the Peptide Conjugates

To evaluate the cellular uptake and localization of the iridium­(III)–peptide conjugates, two cancer cell lines MDA-MB-231 (high expression levels of CAIX and EGFR) and MCF-7 (low expression levels of CAIX and EGFR), and one normal cell line HEK-293 were utilized as the models.^68,69^ As revealed by ICP-MS analyses, the cellular uptake of all three peptide conjugates was substantially higher in MDA-MB-231 cells (0.21–1.8 fmol/cell) compared to MCF-7 (0.074–0.10 fmol/cell) and HEK-293 cells (0.033–0.10 fmol/cell) (Table S15). This observation aligns with the higher abundance of the target CAIX and EGFR proteins in MDA-MB-231 cells and the specific targeting capabilities of the peptide conjugates. In contrast, the peptide-free conjugate **3a-Cys** showed similar levels of cellular uptake in all three cell lines (0.040–0.30 fmol/cell; Table S15), indicating its lack of selectivity. The cellular uptake mechanism and pathways of the conjugates were further studied. For conjugate **3a-CASP**, treatment of MDA-MB-231 cells with the conjugate (10 μM) at 4 °C led to a notable decrease in intracellular iridium content by ca. 70% (Figure S21), suggesting that the conjugate was internalized through an energy-dependent mechanism. The cellular uptake efficiency of the conjugate was reduced by ca. 30% when the cells were pretreated with a clathrin-mediated endocytosis inhibitor chlorpromazine (30 μM, 1 h) (Figure S22). In contrast, it remained largely unchanged when the cells were pretreated with other endocytosis inhibitors, such as a macropinocytosis inhibitor 5-(*N*-ethyl-*N*-isopropyl)­amiloride (EIPA) (50 μM, 1.5 h) and a caveolin-mediated endocytosis inhibitor methyl-β-cyclodextrin (Me-β-CD) (5 mM, 1 h). These findings suggest that conjugate **3a-CASP** was internalized into the cells through an energy-dependent pathway, likely involving clathrin-mediated endocytosis. For conjugates **3a-CYNT** and **3a-CMYI**, pretreatment of the cells with a CAIX inhibitor acetazolamide (1 mM, 6 h) or an EGFR inhibitor gefitinib (50 μM, 1 h) only reduced the uptake of the conjugates by ca. 20 and 10% (Figures S23 and S24), respectively. These findings implied that the modification of the tumor-targeting peptides with an iridium­(III) complex modulated their uptake pathways, which is probably associated with their cationic charge and high lipophilicity.

The intracellular distribution of the conjugates was studied by LSCM. Live MDA-MB-231, MCF-7, and HEK-293 cells were incubated with conjugates **3a-Cys**, **3a-CASP**, **3a-CYNT**, and **3a-CMYI** (10 μM) for 16 h. As depicted in the LSCM images, after incubation with the peptide conjugates, MDA-MB-231 cells displayed more pronounced intracellular emission compared to MCF-7 and HEK-293 cells (Figure 6). However, treatment with the Cys conjugate **3a-Cys** resulted in similar emission intensities across the three cell lines (Figure 6). These observations correlate well with the respective cellular uptake efficiencies of the conjugates (Table S15). Co-staining experiments demonstrate that the conjugates were primarily localized in the lysosomes of MDA-MB-231 cells after uptake (Figure 7 and Figure S25).

**Figure 6:**
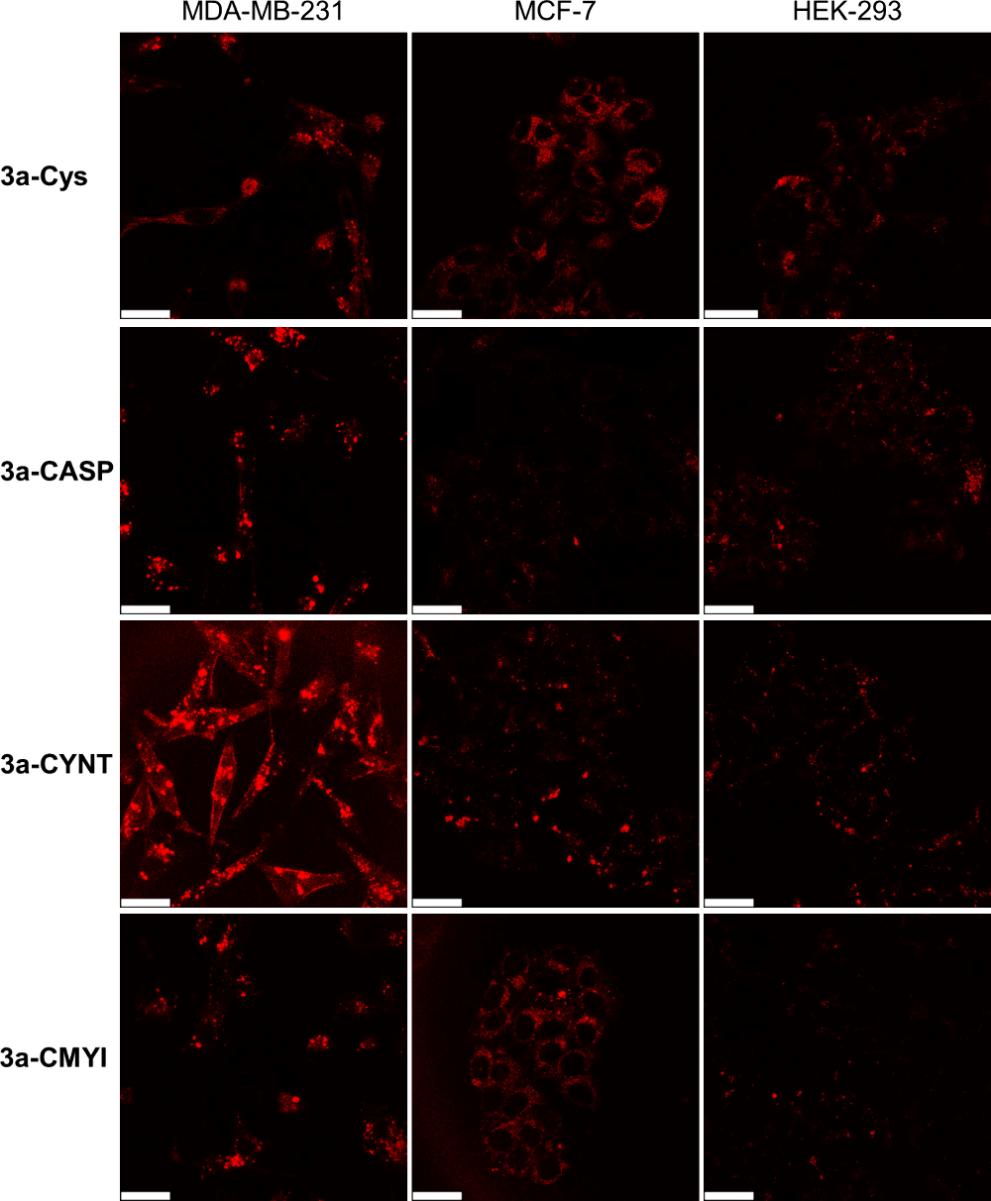
LSCM images of live MDA-MB-231, MCF-7, and HEK-293 cells incubated with conjugates **3a-Cys**, **3a-CASP**, **3a-CYNT**, and **3a-CMYI** (10 μM, 16 h, λ_ex_ = 488 nm, λ_em_ = 650–750 nm). Scale bars = 25 μm.

**Figure 7:**
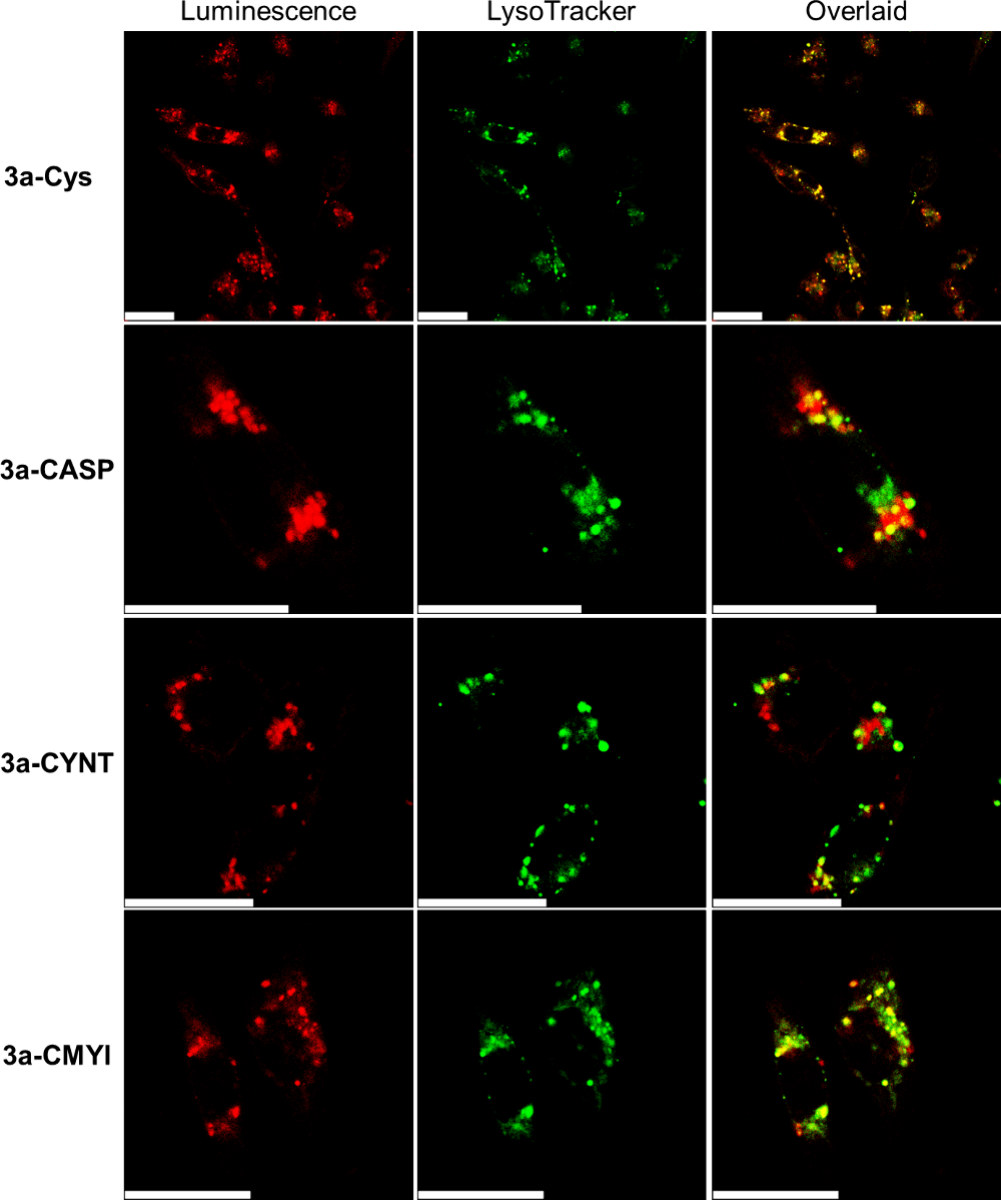
LSCM images of live MDA-MB-231 cells incubated with conjugates **3a-Cys**, **3a-CASP**, **3a-CYNT**, and **3a-CMYI** (10 μM, 16 h, λ_ex_ = 488 nm, λ_em_ = 650–750 nm) and further incubated with LysoTracker Deep Red (100 nM, 1 h, λ_ex_ = 635 nm, λ_em_ = 660–680 nm). Pearson’s correlation coefficient (PCC) = 0.80 (**3a-Cys**), 0.72 (**3a-CASP**), 0.69 (**3a-CYNT**), and 0.81 (**3a-CMYI**). Scale bars = 25 μm.

The (photo)­cytotoxicity of the conjugates was studied using the MTT assay. Conjugates **3a-CASP**, **3a-CYNT**, and **3a-CMYI** all exhibited minimal dark cytotoxicity (IC_50,dark_ > 25 μM; Table 1) across the three cell lines. Upon irradiation (450 nm, 14.6 mW cm^–2^, 10 min), all conjugates showed the highest photocytotoxicity toward MDA-MB-231 cells, with IC_50,light_ values ranging from 0.18 to 0.41 μM (Table 1). The photocytotoxic effects of the conjugates were consistent among MCF-7 and HEK-293 cells (IC_50,light_ = 0.65–1.4 μM and 0.56–1.4 μM, respectively; Table 1), correlating with their comparatively lower cellular uptake efficiencies in these two cell lines (Table S15). These results collectively demonstrate that conjugates **3a-CASP**, **3a-CYNT**, and **3a-CMYI** retained the tumor-targeting properties of the original peptides, and the combined action of photocytotoxic iridium­(III) complexes and tumor-targeting peptides in these conjugates facilitates precise and effective cancer-targeted PDT.

**Table 1:** (Photo)­cytotoxicity of the Peptide Conjugates of Complex **3a** toward MDA-MB-231, MCF-7, and HEK-293 Cells^a^

conjugate	MDA-MB-231			MCF-7			HEK-293		
	IC_50,dark_/μM	IC_50,light_/μM	PI	IC_50,dark_/μM	IC_50,light_/μM	PI	IC_50,dark_/μM	IC_50,light_/μM	PI
**3a-CASP**	>25	0.41 ± 0.01	>61	>25	1.4 ± 0.1	>18	>25	1.4 ± 0.1	>18
**3a-CYNT**	>25	0.18 ± 0.01	>139	>25	0.65 ± 0.03	>38	>25	0.56 ± 0.02	>45
**3a-CMYI**	>25	0.23 ± 0.01	>109	>25	1.0 ± 0.1	>25	>25	0.90 ± 0.08	>28

a PI is the ratio IC_50,dark_/IC_50,light_ under different conditions. The cells were first incubated with the conjugates in the dark for 24 h, replaced with fresh medium, and then incubated in the dark or irradiated at 450 nm (light dosage = 14.6 mW cm^–2^) for 10 min, and subsequently incubated in the dark for 24 h.

## Conclusions

Selective and site-specific bioconjugation plays a pivotal role in biochemistry and biomedical research. In this work, we developed a series of iridium­(III) thioester complexes as phosphorogenic labeling reagents specific for N-Cys-containing biomolecules. Although NCL involving thioesters and N-Cys has been investigated for chemical synthesis of peptides and proteins, the role of thioesters as an emission quenching moiety remains underexplored, and most thioester-based fluorogenic probes for N-Cys typically employ thioester as a responsive linker, connecting a fluorophore to a quencher to enable quenching through PeT or Förster resonance energy transfer. In contrast, our work demonstrates that integrating a thioester moiety into transition metal complexes can effectively modulate their emission and reactive oxygen species photosensitization capabilities. These complexes displayed weak emission in solutions due to the presence of a low-lying nonradiative distorted ^3^IL state localized on the thioester moiety that quenches the emission, as discerned through computational analyses. However, upon NCL reaction with N-Cys, the complexes exhibited substantially increased emission intensities and ^1^O_2_-photosensitization capabilities due to the conversion of the quenching thioester unit to a nonquenching amide linkage. Notably, the emission quenching induced by the ^3^IL (thioester) state is effective across a relatively broad energy range (550–700 nm). Although our computational mechanistic study focuses on iridium­(III) thioester complexes, the proposed strategy should be applicable to other systems beyond these complexes. Specifically, our strategy should be more effective in systems featuring an emissive state of sufficient triplet state energy and efficiency for energy transfer to the designed quenching state involving a large structural distortion. Potential extension of our strategy to other systems may form the basis for future independent work. Additionally, the thioester complexes showed remarkable selectivity toward N-Cys and significantly enhanced reactivity due to the electron-withdrawing iridium­(III) polypyridine moiety. These promising attributes led to the successful applications of complexes **1a** and **3a** as an intracellular Cys sensor and Cys-activatable photosensitizer for cancer-targeted PDT, respectively. Furthermore, complex **3a** was reacted with various N-Cys-modified tumor-targeting peptides to afford photofunctional peptide conjugates that showed high specificity and selective photocytotoxicity toward MDA-MB-231 cells compared to MCF-7 and HEK-293 cells. We believe that our design approach can extend to various organic and inorganic systems, inspiring the development of novel luminogenic thioester-based reagents for bioconjugation, bioimaging, and therapeutic applications.

## Methods

### Physical Measurements and Instrumentation

^1^H and ^13^C NMR spectra were recorded on a Bruker 300, 400, or 600 MHz AVANCE III spectrometer at 298 K using deuterated solvents. Chemical shifts (δ, ppm) were reported relative to tetramethylsilane (TMS) or the residual peak of the deuterated solvent ((CD_3_)_2_CO, 2.05 ppm; CD_3_CN, 1.94 ppm). Positive-ion ESI mass spectra were recorded on a SCIEX API-3200 Triple-Q MS/MS mass spectrometer at 298 K. HR-ESI mass spectra were recorded on a SCIEX X500R Q-TOF at 298 K. IR spectra of the samples in KBr pellets were recorded in the range of 4000–400 cm^–1^ using a PerkinElmer Spectrum 100 FTIR spectrometer. Elemental analyses were carried out on an Elementar Analysensysteme GmbH Vario MICRO elemental analyzer. HPLC was performed on an Agilent 1260 Infinity II system coupled with a diode array detector WR using H_2_O containing 0.1% (*v*/*v*) trifluoroacetic acid (TFA) (solvent A) and CH_3_CN containing 0.1% (*v*/*v*) TFA (solvent B) as the solvents, and the detector was set to 220, 250, and 350 nm.

### Preparation of Iridium­(III)–Cys Conjugates **1a-Cys**–**4a-Cys**

A mixture of the iridium­(III) thioester complex (4 μmol) and l-Cys (200 μmol) in CH_3_CN/potassium phosphate buffer (50 mM, pH 7.0) (10:1, *v*/*v*, 11 mL) was stirred at 298 K in the dark for 18 h. The mixture was diluted with CH_2_Cl_2_ (20 mL), and the organic layer was washed with H_2_O (20 mL × 3), dried over anhydrous MgSO_4_, and filtered. The solvent was removed under reduced pressure and the residual solid was purified by semipreparative reversed-phase HPLC (RP-HPLC). The HPLC purification was carried out on an Agilent semipreparative column (ZORBAX Eclipse XDB-C18 column: 9.4 × 250 mm, 5 μm) using solvent A and solvent B as the solvents, with a linear gradient of 50–100% solvent B over 30 min and a flow rate of 2 mL min^–1^. Fractions containing the product were combined and lyophilized. The orange purified conjugates were characterized by analytical RP-HPLC and ESI-MS. The HPLC analysis was carried out using an Agilent analytical column (ZORBAX Eclipse Plus C18 column: 4.6 × 150 mm, 5 μm) with a linear gradient of 40–100% solvent B over 12 min and a flow rate of 1 mL min^–1^. **1a-Cys**. Yield: 3.4 mg (82%). *t*_R_ = 6.8 min. ^1^H NMR (300 MHz, CD_3_CN, 298 K): δ 8.51 (d, *J* = 1.2 Hz, 1H, H3 of bpy), 8.40–8.27 (m, 5H, H3 and H4 of quinolinyl ring of pq, and H6 of bpy), 8.24–8.10 (m, 4H, CONH, H3′ of bpy, and H3 of phenyl ring of pq), 8.03 (d, *J* = 5.7 Hz, 1H, H6’ of bpy), 7.85–7.77 (m, 3H, H8 of quinolinyl ring of pq and H5 of bpy), 7.39 (td, *J* = 7.6, 2.9 Hz, 2H, H7 of quinolinyl ring of pq), 7.34–7.25 (m, 3H, H5′ of bpy and H5 of quinolinyl ring of pq), 7.17 (t, *J* = 7.6 Hz, 2H, H4 of phenyl ring of pq), 7.13–7.00 (m, 2H, H6 of quinolinyl ring of pq), 6.81 (td, *J* = 7.6, 3.4 Hz, 2H, H5 of phenyl ring of pq), 6.53–6.47 (m, 2H, H6 of phenyl ring of pq), 4.67 (q, *J* = 6.2 Hz, 1H, CH of Cys), 3.07–2.95 (m, 2H, CH_2_ of Cys), 2.40 (s, 3H, CH_3_ of bpy), 1.89 (br, 1H, SH of Cys). MS (ESI, positive mode, *m*/*z*): 918.5 [M – CF_3_COO^–^]^+^. **2a-Cys**. Yield: 3.7 mg (80%). *t*_R_ = 9.7 min. MS (ESI, positive mode, *m*/*z*): 1030.5 [M – CF_3_COO^–^]^+^. **3a-Cys**. Yield: 3.7 mg (80%). *t*_R_ = 9.3 min. MS (ESI, positive mode, *m*/*z*): 1030.5 [M – CF_3_COO^–^]^+^. **4a-Cys**. Yield: 3.9 mg (79%). *t*_R_ = 9.9 min. MS (ESI, positive mode, *m*/*z*): 1130.5 [M – CF_3_COO^–^]^+^.

### Preparation of Peptide Conjugates of Complex **3a**

A mixture of complex **3a** (2 μmol) and N-Cys-containing peptide (CASP, CYNT, and CMYI) (3 μmol) in potassium phosphate buffer (50 mM, pH 7.0)/DMSO (3:2, *v*/*v*, 2 mL) containing TCEP (10 mM) was stirred at 298 K in the dark for 12 h. The solvent was removed under reduced pressure and the residual dark orange solid was purified by semipreparative RP-HPLC. The HPLC purification was carried out on an Agilent semipreparative column (ZORBAX Eclipse XDB-C18 column: 9.4 × 250 mm, 5 μm) using solvent A and solvent B as the solvents, with a linear gradient of 20–100% solvent B over 20 min and a flow rate of 2 mL min^–1^. Fractions containing the product were combined and lyophilized. The dark orange purified conjugates were characterized by analytical RP-HPLC and ESI-MS. The HPLC analysis was carried out using an Agilent analytical column (ZORBAX Eclipse Plus C18 column: 4.6 × 150 mm, 5 μm) with a linear gradient of 40–100% solvent B over 12 min and a flow rate of 1 mL min^–1^. **3a-CASP**. Yield: 3.0 mg (72%). *t*_R_ = 7.3 min. MS (ESI, positive mode, *m*/*z*): 979.7 [M – CF_3_COO^–^ + H^+^]^2+^. **3a-CYNT**. Yield: 3.6 mg (70%). *t*_R_ = 6.9 min. MS (ESI, positive mode, *m*/*z*): 1223.4 [M – CF_3_COO^–^ + H^+^]^2+^, 816.0 [M – CF_3_COO^–^ + 2H^+^]^3+^. **3a-CMYI**. Yield: 3.3 mg (68%). *t*_R_ = 7.7 min. MS (ESI, positive mode, *m*/*z*): 1166.9 [M – CF_3_COO^–^ + H^+^]^2+^.

### X-ray Structural Analysis for Complex **1a**

Single crystals of the complex were obtained by slow diffusion of Et_2_O into a concentrated CH_3_CN solution of the complex at 298 K. Single-X-ray data were collected on a Rigaku Oxford Diffraction, Synergy Custom system, HyPix diffractometer using Cu Kα radiation (1.54184 Å). Cell refinement, data collection, and data reduction were done using CrysAlisPro 1.171.43.127a (Rigaku OD, 2024) program. The structure was resolved by direct methods and refined using full-matrix least-squares on *F* ^2^ with the SHELXL program^70^ through the OLEX2 interface.^71^ All nonhydrogen atoms of the complexes were refined with anisotropic thermal parameters. Hydrogen atoms were placed in idealized positions and refined with fixed geometry with respect to their carrier atoms. The structure features a positional disorder on the bpy-COSBn ligand with a 4:1 ratio under *C*_2_ symmetry. The predominant conformation (80%) is depicted in Figure 1.

### Stability Studies

The thioester complex **1a** (10 μM) was incubated in potassium phosphate buffer (50 mM, pH 7.0)/CH_3_CN (3:2, *v*/*v*) containing TCEP (1 mM) at 37 °C in the dark for 1 h. The solution was extracted with CH_2_Cl_2_ (1 mL × 3). The organic extract was dried over anhydrous MgSO_4_, filtered, and the solvent was removed under reduced pressure. The residue was dissolved in CH_3_CN and analyzed by ESI-MS.

### Selectivity Studies

For the chemoselectivity studies, a mixture of complex **1a** (20 μM) and l-Lys (2 mM), l-His (2 mM), l-Ser (2 mM), or l-Thr (2 mM) was incubated in potassium phosphate buffer (50 mM, pH 7.0)/DMSO (3:2, *v*/*v*) containing TCEP (10 mM) at 298 K in the dark for 1 h. For the regioselectivity studies, a mixture of complex **1a** (25 μM) and the tripeptide CSS, SCS, or SSC (1 mM) was incubated in potassium phosphate buffer (50 mM, pH 7.0)/DMSO (3:2, *v*/*v*) containing TCEP (10 mM) at 298 K in the dark for 1 h. An aliquot of the reaction mixture (20 μL) was analyzed by RP-HPLC. The HPLC analysis was carried out using an Agilent analytical column (ZORBAX Eclipse Plus C18 column: 4.6 × 150 mm, 5 μm) with a linear gradient of 40–100% solvent B over 12 min and a flow rate of 1 mL min^–1^.

### Phosphorogenic Response of Thioester Complexes toward Thiols

The thioester complexes **1a**–**4a** (10 μM) were incubated with l-Cys (100 μM) in aerated potassium phosphate buffer (50 mM, pH 7.0)/CH_3_CN (3:2, *v*/*v*) containing TCEP (1 mM) at 298 K in the dark for 1 h. The emission spectra were recorded on a HORIBA FluoroMax-4 spectrofluorometer and the emission lifetimes were measured on an Edinburgh Instruments FLS980 spectrometer. The emission intensities were determined by the areas under the emission spectra before and after incubation with l-Cys.

An emission titration experiment was conducted by the gradual addition of l-Cys to a solution of complex **1a** (10 μM) in aerated potassium phosphate buffer (50 mM, pH 7.0)/CH_3_CN (3:2, *v*/*v*) containing TCEP (1 mM) at 298 K. The emission spectra were recorded on a HORIBA FluoroMax-4 spectrofluorometer. Each emission spectrum was acquired 4 min after mixing.

For the selectivity studies, a mixture of complex **1a** (10 μM) and l-Cys (100 μM), ethanethiol (100 μM), or GSH (100 μM) was incubated in aerated potassium phosphate buffer (50 mM, pH 7.0)/CH_3_CN (3:2, *v*/*v*) containing TCEP (1 mM) at 298 K in the dark for 1 h. The emission spectra were recorded on a HORIBA FluoroMax-4 spectrofluorometer. The emission intensities were determined by the areas under the emission spectra before and after incubation with thiols.

### Cell Cultures

HeLa, MDA-MB-231, MCF-7, and HEK-293 cells were grown in DMEM supplemented with 10% FBS and 1% penicillin/streptomycin at 37 °C under a 5% CO_2_ atmosphere. They were subcultured every 2 to 3 days.

### Live-Cell Confocal Imaging

Complex **1a**: HeLa cells in growth medium were seeded in a 35-mm confocal dish and grown at 37 °C under a 5% CO_2_ atmosphere for 48 h. The growth medium was removed, and the cells were washed with PBS (1 mL × 3) and incubated with complex **1a** (10 μM) in medium/DMSO (99:1, *v*/*v*) at 37 °C for 1 h. After the treatment, the medium was removed, and the cells were washed with PBS (1 mL × 3) and imaged using a Leica TCS SPE confocal microscope (inverted configuration) with a 63× oil-immersion objective lens. The excitation wavelength of complex **1a** was 405 nm. For the control experiments, the cells were treated with NEM (100 μM) at 37 °C for 20 min with or without posttreatment with l-Cys (100 μM) at 37 °C for 30 min prior to incubation with complex **1a**. The intracellular emission spectra were recorded using a λ-scanning mode. HeLa cells were treated with complex **1a** (10 μM, 1 h) or conjugate **1a-Cys** (20 μM, 6 h). The incubation period was longer for the conjugate due to its poorer cellular uptake efficiency compared with complex **1a**.

Conjugates **3a-Cys**, **3a-CASP**, **3a-CYNT**, and **3a-CMYI**: MDA-MB-231, MCF-7, or HEK-293 cells in growth medium were seeded in a 35-mm confocal dish and grown at 37 °C under a 5% CO_2_ atmosphere for 48 h. The growth medium was removed, and the cells were washed with PBS (1 mL × 3) and incubated with the conjugates (10 μM) in medium/DMSO (99:1, *v*/*v*) at 37 °C for 16 h. After the treatment, the medium was removed, and the cells were washed with PBS (1 mL × 3) and imaged using a Leica TCS SPE confocal microscope (inverted configuration) with a 63× oil-immersion objective lens. The excitation wavelength of the conjugates was 488 nm. For the co-staining experiments in MDA-MB-231 cells, after treatment with the conjugates, the medium was removed, and the cells were washed with PBS (1 mL × 3) and incubated with LysoTracker Deep Red (100 nM) for 1 h or MitoTracker Green (100 nM) for 20 min in growth medium at 37 °C. The medium was then removed, and the cells were washed with PBS (1 mL × 3) and imaged using a Leica TCS SPE confocal microscope (inverted configuration) with a 63× oil-immersion objective lens. The excitation wavelength of LysoTracker Deep Red and MitoTracker Green were 635 and 488 nm, respectively. The PCC values were determined using the program ImageJ (Version 1.4.3.67).

### MTT Assays

HeLa or HEK-293 cells were seeded in two 96-well flat-bottomed microplates (ca. 10,000 cells per well) in growth medium (100 μL) and incubated at 37 °C under a 5% CO_2_ atmosphere for 48 h. The growth medium was removed and replaced with medium/DMSO (100 μL, 99:1, *v*/*v*) containing complex **3a** with concentrations ranging from 10^–5^ to 10^–8^ M. Wells containing untreated cells were used as blank control. The microplates were incubated at 37 °C in the dark under a 5% CO_2_ atmosphere for 2 h. After the treatment, the medium was removed and replenished with phenol red-free growth medium (100 μL). One of the microplates was irradiated at 450 nm (14.6 mW cm^–2^) for 10 min with an LED cellular photocytotoxicity irradiator (PURI Materials, Shenzhen, China) and the other microplate was kept in the dark. Then the growth medium was replaced with fresh medium and the cells were further incubated at 37 °C under a 5% CO_2_ atmosphere for 24 h. The medium in each well was then replaced with fresh medium (90 μL) and 10 μL of MTT (5 mg mL^**–**1^) in PBS. The microplates were incubated at 37 °C under a 5% CO_2_ atmosphere for 4 h. The growth medium was then removed, and DMSO (100 μL) was added to each well. After 20 min, the absorbance of the solutions at 570 nm was measured with an Multiskan SkyHigh Microplate Spectrophotometer (Thermo Scientific). The IC_50_ values of the complex were determined from dose dependence of surviving cells after the treatment using the OriginPro 8.0 software package. For conjugates **3a-CASP**, **3a-CYNT**, and **3a-CMYI**, the procedure was similar to that of complex **3a**, except that MDA-MB-231, MCF-7, and HEK-293 cells were used and the conjugates were incubated for 24 h instead of 2 h before irradiation.

### Cellular Uptake Measurements

HeLa or HEK-293 cells were seeded in a 35-mm tissue culture dish and incubated at 37 °C under a 5% CO_2_ atmosphere for 48 h. The culture medium was removed and replaced with complexes **1a** and **3a** (10 μM) in medium/DMSO (99:1, *v*/*v*) at 37 °C under a 5% CO_2_ atmosphere for 2 h. After the treatment, the medium was removed, and the cells were washed with PBS (1 mL × 3). The cells were then trypsinized and harvested with PBS (1 mL). The cell numbers were obtained by a Logos Biosystems LUNA-II automated cell counter. The resultant solution was digested with 65% HNO_3_ (1 mL) at 70 °C for 2 h, allowed to cool to room temperature, and analyzed by a NexION 2000 ICP-MS (PerkinElmer SCIEX Instruments). For conjugates **3a-Cys**, **3a-CASP**, **3a-CYNT**, and **3a-CMYI**, the procedure was similar to that of complexes **1a** and **3a**, except that MDA-MB-231, MCF-7, and HEK-293 cells were used and the conjugates were incubated for 16 h instead of 2 h before the measurements.

### Cellular Uptake Mechanism Studies

Conjugate **3a-CASP**: MDA-MB-231 cells were seeded in a 35-mm tissue culture dish and incubated at 37 °C under a 5% CO_2_ atmosphere for 48 h. The culture medium was removed and replaced with conjugate **3a-CASP** (10 μM) in medium/DMSO (99:1, *v*/*v*) at 37 °C under a 5% CO_2_ atmosphere for 4 or 16 h. In the low-temperature experiments, the cells were preincubated at 4 °C for 1 h prior to incubation with conjugate **3a-CASP** (10 μM) in medium/DMSO (99:1, *v*/*v*) at 37 °C under a 5% CO_2_ atmosphere for 4 h. In the chemical inhibition experiments, the cells were pretreated with EIPA (50 μM) for 1.5 h, Me-β-CD (5 mM) for 1 h, or chlorpromazine (30 μM) for 1 h at 37 °C under a 5% CO_2_ atmosphere. The cells were then washed with PBS (1 mL × 3) and incubated with conjugate **3a-CASP** (10 μM) in medium/DMSO (99:1, *v*/*v*) at 37 °C under a 5% CO_2_ atmosphere for 16 h.

Conjugate **3a-CYNT**: MDA-MB-231 cells were seeded in a 35-mm tissue culture dish and incubated at 37 °C under a 5% CO_2_ atmosphere for 48 h. The culture medium was removed and replaced with conjugate **3a-CYNT** (10 μM) in medium/DMSO (99:1, *v*/*v*) at 37 °C under a 5% CO_2_ atmosphere for 16 h. In the CAIX inhibition experiments, the cells were pretreated with acetazolamide (1 mM) at 37 °C under a 5% CO_2_ atmosphere for 6 h. The cells were then washed with PBS (1 mL × 3) and incubated with conjugate **3a-CYNT** (10 μM) in medium/DMSO (99:1, *v*/*v*) at 37 °C under a 5% CO_2_ atmosphere for 16 h.

Conjugate **3a-CMYI**: MDA-MB-231 cells were seeded in a 35-mm tissue culture dish and incubated at 37 °C under a 5% CO_2_ atmosphere for 48 h. The culture medium was removed and replaced with conjugate **3a-CMYI** (10 μM) in medium/DMSO (99:1, *v*/*v*) at 37 °C under a 5% CO_2_ atmosphere for 16 h. In the EGFR inhibition experiments, the cells were pretreated with gefitinib (50 μM) at 37 °C under a 5% CO_2_ atmosphere for 1 h. The cells were then washed with PBS (1 mL × 3) and incubated with conjugate **3a-CMYI** (10 μM) in medium/DMSO (99:1, *v*/*v*) at 37 °C under a 5% CO_2_ atmosphere for 16 h.

For all the experiments, after incubation with the conjugates, the medium was removed, and the cells were washed with PBS (1 mL × 3). The cells were then trypsinized and harvested with PBS (1 mL). The cell numbers were measured by a Logos Biosystems LUNA-II automated cell counter. The resultant solution was digested with 65% HNO_3_ (1 mL) at 70 °C for 2 h, allowed to cool to room temperature, and analyzed by a NexION 2000 ICP-MS (PerkinElmer SCIEX Instruments).

Additional experimental procedures and data processing methods are detailed in the Supporting Information.

## Supplementary Material



## Notes

Additional experimental procedures, X-ray crystal data, UV–vis absorption spectra, photophysical data, electrochemical data, computational data, ESI-MS data, HPLC chromatograms, reaction kinetics, cellular uptake data, and ^1^H and ^13^C NMR spectra (PDF)
