# Supplementary material for: Leveraging the Aminothiol-Specific Phosphorogenic Response of Iridium(III) Thioester Complexes for the Development of Intracellular Sensors and Cancer Phototherapeutics
Source: JACS Au. 2025 Jun 10;5(6):2825–36. doi: 10.1021/jacsau.5c00413 (PMC12188478; doi:10.1021/jacsau.5c00413)
Supplement: Supplementary file 1 [file au5c00413_si_001.pdf]

Supporting Information

Leveraging the Aminothiol-Specific Phosphorogenic Response of Iridium(III) Thioester Complexes for the Development of Intracellular Sensors and Cancer Phototherapeutics

Eunice Chiu-Lam Mak,[†] Ziyong Chen,[‡] Lawrence Cho-Cheung Lee,[†] Liang-Liang Yan,[Φ] Vivian Wing-Wah Yam,*[,‡] and Kenneth Kam-Wing Lo*[,†,§]

[†]Department of Chemistry, City University of Hong Kong, Kowloon, Hong Kong, P. R. China

[‡]Institute of Molecular Functional Materials and Department of Chemistry, The University of Hong Kong, Pokfulam Road, Hong Kong, P. R. China

[Φ]State Key Laboratory of Structural Chemistry, Fujian Institute of Research on the Structure of Matter, Chinese Academy of Sciences, Fuzhou 350002, P. R. China

[§]State Key Laboratory of Terahertz and Millimeter Waves, City University of Hong Kong, Kowloon, Hong Kong, P. R. China

Table of Contents

**Experimental Section**		S10
**Table S1**	Crystallographic data of complex **1a**.	S33
**Table S2**	Selected bond lengths (Å) and bond angles (°) for complex **1a**.	S35
**Table S3**	Electronic absorption spectral data of the iridium(III) complexes at 298 K.	S36
**Table S4**	Photophysical data of the iridium(III) complexes.	S38
**Table S5**	$^1O_2$ generation quantum yields (Φ_Δ) of the iridium(III) complexes in aerated CH_3CN at 298 K.	S40
**Table S6**	Electrochemical data of the thioester complexes **1a** – **4a** and ligand bpy-COSBn.[a]	S41
**Table S7**	Photophysical data of complexes **1a** – **4a** (10 μM) before and after incubation with L-Cys (100 μM) in aerated potassium phosphate buffer (50 mM, pH 7.0)/CH_3CN (3:2, *v*/*v*) containing TCEP (1 mM) at 298 K for 1 h.	S42
**Table S8**	Photophysical data of the iridium(III) Cys and peptide conjugates in degassed solvents at 298 K.	S43
**Table S9**	$^1O_2$ generation quantum yields (Φ_Δ) of the iridium(III)–Cys conjugates in aerated CH_3CN at 298 K.	S44
**Table S10**	Relative energies (eV) of the structures at the relaxed ^{3}MLCT state, relaxed ^{3}MC state, ^{3}MC/S_0 MECP, relaxed ^{3}IL state, and ^{3}IL/S_0 MECP with respect to that of the ground state (S_0).	S45
**Table S11**	H_{SOC} constants (cm^{-1}) between S_0 and ^{3}IL states computed at optimized ^{3}IL/S_0 MECP of **1a-Me**. The computed rate constants for	S46

^{3}IL → S_0 ISC (k_{ISC}) and ^{3}IL → S_0 phosphorescence (k_r^P) are provided. Here, *i* is the imaginary unit.

Table S12 Emission enhancement factors (I/I_o) of complex **1a** (10 μM) upon incubation with thiols (100 μM) in aerated potassium phosphate buffer (50 mM, pH 7.0)/CH_3CN (3:2, *v*/*v*) containing TCEP (1 mM) at 298 K for 1 h. S47

Table S13 Cellular uptake and (photo)cytotoxicity of complex **3a** in MDA-MB-231 and HEK-293 cells. PI is the ratio $IC_{50,dark}/IC_{50,light}$. S48

Table S14 $^1O_2$ generation quantum yields (Φ_Δ) of the Cys and peptide conjugates of complex **3a** in aerated CH_3CN/H_2O (4:1, *v*/*v*) at 298 K. S49

Table S15 Cellular uptake of the conjugates of complex **3a** in live cells. S50

Table S16 Cartesian coordinates of the optimized S_0 structure of complex **1a-Me**. S51

Table S17 Cartesian coordinates of the optimized ^{3}MLCT structure of complex **1a-Me**. S52

Table S18 Cartesian coordinates of the optimized ^{3}MC structure of complex **1a-Me**. S53

Table S19 Cartesian coordinates of the optimized ^{3}MC/S_0 MECP structure of complex **1a-Me**. S54

Table S20 Cartesian coordinates of the optimized ^{3}IL structure of complex **1a-Me**. S55

Table S21 Cartesian coordinates of the optimized ^{3}IL/S_0 MECP structure of complex **1a-Me**. S56

Table S22 Cartesian coordinates of the optimized S_0 structure of complex **1a-Cys**. S57

**Table S23**	Cartesian coordinates of the optimized ^{3}MLCT structure of complex **1a-Cys**.	S58
**Table S24**	Cartesian coordinates of the optimized ^{3}MC structure of complex **1a-Cys**.	S59
**Table S25**	Cartesian coordinates of the optimized ^{3}MC/S_0 MECP structure of complex **1a-Cys**.	S60
**Table S26**	Cartesian coordinates of the optimized S_0 structure of complex **1b**.	S61
**Table S27**	Cartesian coordinates of the optimized ^{3}MLCT structure of complex **1b**.	S62
**Table S28**	Cartesian coordinates of the optimized ^{3}MC structure of complex **1b**.	S63
**Table S29**	Cartesian coordinates of the optimized ^{3}MC/S_0 MECP structure of complex **1b**.	S64
**Figure S1**	Electronic absorption spectra of complexes **1a** – **4a** in CH_2Cl_2 (black) and CH_3CN (red) at 298 K.	S65
**Figure S2**	Electronic absorption spectra of complexes **1b** – **4b** in CH_2Cl_2 (black) and CH_3CN (red) at 298 K.	S66
**Figure S3**	Normalized emission spectra of complexes **1a** – **4a** in degassed CH_2Cl_2 (black) and CH_3CN (red) at 298 K and alcohol glass at 77 K (blue).	S67
**Figure S4**	Normalized emission spectra of complexes **1b** – **4b** in degassed CH_2Cl_2 (black) and CH_3CN (red) at 298 K and alcohol glass at 77 K (blue).	S68
**Figure S5**	Latimer diagrams showing the excited-state redox potentials of complexes **1a** – **4a** versus SCE.	S69

Figure S6 HPLC chromatograms of the reaction mixtures of complexes **1a** – **4a** (20 μM) without (black) or with L-Cys (25 μM) (red) in aerated potassium phosphate buffer (50 mM, pH 7.0)/DMSO (3:2, *v*/*v*) containing TCEP (250 μM) after incubation at 298 K for 1 h. The absorbance was monitored at 350 nm. S70

Figure S7 ESI mass spectra of the reaction mixtures of complexes **1a** – **4a** (20 μM) with L-Cys (25 μM) in aerated potassium phosphate buffer (50 mM, pH 7.0)/DMSO (3:2, *v*/*v*) containing TCEP (250 μM) after incubation at 298 K for 1 h. S71

Figure S8 ^{1}H NMR spectrum of conjugate **1a-Cys** in CD_3CN at 298 K. S72

Figure S9 ^{1}H–^{1}H COSY NMR spectrum of conjugate **1a-Cys** in CD_3CN at 298 K. S73

Figure S10 Second-order kinetics for the reaction of the thioester complexes **1a** – **4a** (20 μM) and ligand bpy-COSBn (100 μM) with L-Cys (25 μM for the complexes and 250 μM for the ligand) at different time points in aerated potassium phosphate buffer (50 mM, pH 7.0)/DMSO (3:2, *v*/*v*) containing TCEP (250 μM) after incubation at 298 K. The slope of the linear fit corresponds to the k_2 of the reaction. S74

Figure S11 HPLC chromatograms of complex **1a** (20 μM) (black) and the reaction mixtures of complex **1a** (20 μM) upon incubation with L-Lys (2 mM), L-His (2 mM), L-Ser (2 mM), or L-Thr (2 mM) (red) in aerated potassium phosphate buffer (50 mM, pH 7.0)/DMSO (3:2, *v*/*v*) containing TCEP (10 mM) at 298 K for 1 h. The absorbance was monitored at 350 nm. S75

Figure S12 Emission spectra of complex **1a** (10 μM) upon the gradual addition of L-Cys in aerated potassium phosphate buffer (50 mM, pH 7.0)/CH_3CN (3:2, *v*/*v*) containing TCEP (1 mM) at 298 K. S76

Figure S13 Normalized emission spectra of conjugates **1a-Cys** – **4a-Cys** in degassed CH_3CN (black) and potassium phosphate buffer (50 mM, pH 7.4)/MeOH (2:3, *v*/*v*) (red) at 298 K. S77

Figure S14 Emission spectra and ESI mass spectra of the reaction mixtures of complex **1a** (10 μM) with (a) L-Cys, (b) ethanethiol, and (c) GSH (100 μM) in aerated potassium phosphate buffer (50 mM, pH 7.0)/CH_3CN (3:2, *v*/*v*) containing TCEP (1 mM) at 298 K for 1 h. S78

Figure S15 ESI mass spectrum of a CH_2Cl_2 extract of complex **1a** (10 μM) in aerated potassium phosphate buffer (50 mM, pH 7.0)/CH_3CN (3:2, *v*/*v*) containing TCEP (1 mM) after incubation at 37°C for 1 h. S79

Figure S16 Normalized intracellular emission spectra of HeLa cells incubated with (a) complex **1a** (10 μM, 1 h, λ_{ex} = 405 nm) and (b) conjugate **1a-Cys** (20 μM, 6 h, λ_{ex} = 405 nm). S80

Figure S17 Semipreparative HPLC chromatograms of the crude reaction mixtures of complex **3a** (1 mM) and peptides CASP, CYNT, and CMYI (1.5 mM) in aerated potassium phosphate buffer (50 mM, pH 7.0)/DMSO (3:2, *v*/*v*) containing TCEP (10 mM) at 298 K for 12 h. The absorbance was monitored at 350 nm. S81

Figure S18 Analytical HPLC chromatograms of the purified conjugates **3a-Cys**, **3a-CASP**, **3a-CYNT**, and **3a-CMYI**. The absorbance was monitored at 350 nm. S82

Figure S19 ESI mass spectra of the purified conjugates **3a-Cys**, **3a-CASP**, **3a-CYNT**, and **3a-CMYI** in CH_3CN. S83

Figure S20 Normalized emission spectra of the peptide conjugates of complex **3a** in degassed potassium phosphate buffer (50 mM, pH 7.4)/MeOH (2:3, *v*/*v*) at 298 K. S84

Figure S21 Relative amounts of iridium associated with an average MDA-MB-231 cell upon incubation with conjugate **3a-CASP** (10 μM, 4 h) at 37°C without or with preincubation of the cells at 4°C for 1 h. The uptake values at 37°C were taken as the reference. S85

Figure S22 Relative amounts of iridium associated with an average MDA-MB-231 cell upon incubation with conjugate **3a-CASP** (10 μM, 16 h) at 37°C without or with preincubation of the cells with EIPA (50 μM, 1.5 h), Me-β-CD (5 mM, 1 h), or chlorpromazine (30 μM, 1 h). The uptake values at 37°C without pretreatment were taken as the reference. S86

Figure S23 Relative amounts of iridium associated with an average MDA-MB-231 cell upon incubation with conjugate **3a-CYNT** (10 μM, 16 h) at 37°C without or with preincubation of the cells with acetazolamide (1 mM, 6 h). The uptake values at 37°C without pretreatment were taken as the reference. S87

Figure S24 Relative amounts of iridium associated with an average MDA-MB-231 cell upon incubation with conjugate **3a-CMYI** (10 μM, 16 h) at 37°C without or with preincubation of the cells with gefitinib (50 μM, 1 h). The uptake values at 37°C without pretreatment were taken as the reference. S88

Figure S25 LSCM images of live MDA-MB-231 cells incubated with conjugates **3a-Cys**, **3a-CASP**, **3a-CYNT**, and **3a-CMYI** (10 μM, 16 h, λ_{ex} = 488 nm, λ_{em} = 650 – 750 nm), and further incubated with MitoTracker Green (100 nM, 20 min, λ_{ex} = 488 nm, λ_{em} = 505 – 525 nm). PCC = 0.43 (**3a-Cys**), 0.46 (**3a-CASP**), 0.36 (**3a-CYNT**), and 0.28 (**3a-CMYI**). Scale bars = 25 μm. S89

Figure S26 Comparison of the geometric structures of the S_0 state (black), ^{3}MLCT state (red), ^{3}MC state (magenta), ^{3}MC/S_0 MECP (lime), ^{3}IL state (blue), and ^{3}IL/S_0 MECP (orange) for complexes **1a-Me**, **1a-Cys**, and **1b**. The root-mean-square deviations (Å) with respect to the geometric structure of S_0 state are provided. Hydrogen atoms are removed for clarity. S90

Figure S27 Schematic diagrams of potential energy profiles (eV) along the nuclear coordinate (*Q*) for complexes (a) **1a-Me** and (b) **1b**. Relative energies of the optimized S_0, ^{3}MLCT, ^{3}IL, and ^{3}MC structures on the S_0 and T_1 surfaces are provided. S91

Figure S28 ^{1}H NMR spectrum of bpy-COSBn in $(CD_3)_2CO$ at 298 K. S92

Figure S29 ^{1}H NMR spectrum of complex **1a** in $(CD_3)_2CO$ at 298 K. S93

Figure S30 ^{13}C NMR spectrum of complex **1a** in $(CD_3)_2CO$ at 298 K. S94

Figure S31 (a) Experimental and (b) simulated HR-ESI mass spectra of complex **1a** in CH_3CN. S95

Figure S32 ^{1}H NMR spectrum of complex **1b** in $(CD_3)_2CO$ at 298 K. S96

Figure S33 ^{13}C NMR spectrum of complex **1b** in $(CD_3)_2CO$ at 298 K. S97

Figure S34 (a) Experimental and (b) simulated HR-ESI mass spectra of complex **1b** in CH_3CN. S98

Figure S35 ^{1}H NMR spectrum of complex **2a** in $(CD_3)_2CO$ at 298 K. S99

Figure S36 ^{13}C NMR spectrum of complex **2a** in $(CD_3)_2CO$ at 298 K. S100

Figure S37 (a) Experimental and (b) simulated HR-ESI mass spectra of complex **2a** in CH_3CN. S101

Figure S38 ^{1}H NMR spectrum of complex **2b** in $(CD_3)_2CO$ at 298 K. S102

Figure S39 ^{13}C NMR spectrum of complex **2b** in $(CD_3)_2CO$ at 298 K. S103

Figure S40 (a) Experimental and (b) simulated HR-ESI mass spectra of complex **2b** in CH_3CN. S104

Figure S41 ^{1}H NMR spectrum of complex **3a** in $(CD_3)_2CO$ at 298 K. S105

Figure S42 ^{13}C NMR spectrum of complex **3a** in $(CD_3)_2CO$ at 298 K. S106

Figure S43 (a) Experimental and (b) simulated HR-ESI mass spectra of complex **3a** in CH_3CN. S107

Figure S44 ^{1}H NMR spectrum of complex **3b** in $(CD_3)_2CO$ at 298 K. S108

Figure S45 ^{13}C NMR spectrum of complex **3b** in $(CD_3)_2CO$ at 298 K. S109

Figure S46 (a) Experimental and (b) simulated HR-ESI mass spectra of complex **3b** in CH_3CN. S110

Figure S47 ^{1}H NMR spectrum of complex **4a** in $(CD_3)_2CO$ at 298 K. S111

Figure S48 ^{13}C NMR spectrum of complex **4a** in $(CD_3)_2CO$ at 298 K. S112

Figure S49 (a) Experimental and (b) simulated HR-ESI mass spectra of complex **4a** in CH_3CN. S113

Figure S50 ^{1}H NMR spectrum of complex **4b** in $(CD_3)_2CO$ at 298 K. S114

Figure S51 ^{13}C NMR spectrum of complex **4b** in $(CD_3)_2CO$ at 298 K. S115

Figure S52 (a) Experimental and (b) simulated HR-ESI mass spectra of complex **4b** in CH_3CN. S116

References S117

Experimental Section

Materials and Synthesis

All solvents were of analytical reagent grade and purified according to standard procedures.[1] 4-Dimethylaminopyridine (DMAP), $NaHCO_3$, $MgSO_4$, KPF_6, trifluoroacetic acid (TFA), L-histidine (L-His), L-serine (L-Ser), and L-threonine (L-Thr) were purchased from Acros. 4,4'-Dimethyl-2,2'-bipyridine, $IrCl_3{\cdot}3H_2O$, 2-phenylquinoline (Hpq), L-cysteine (L-Cys), ethanethiol, glutathione (GSH), 3-(4,5-dimethylthiazol-2-yl)-2,5-diphenyltetrazolium bromide, *N*-ethylmaleimide, and methyl-β-cyclodextrin (Me-β-CD) were purchased from Aldrich. Benzyl mercaptan was purchased from Macklin. *N*,*N*-Diisopropylethylamine (DIPEA) was purchased from Energy Chemical. Tris(2-carboxyethyl)phosphine hydrochloride (TCEP) was purchased from Aladdin. Acetazolamide was sourced from Thermo Scientific (Alfa Aesar). L-Lysine (L-Lys), chlorpromazine hydrochloride, and gefitinib were sourced from Tokyo Chemical Industry. 5-(*N*-Ethyl-*N*-isopropyl)amiloride (EIPA) was sourced from J&K Scientific. The peptides CSS, SCS, SSC, CASPSGALRSC (CASP), CYNTNHVPLSPKY (CYNT), and CMYIEALDKYAC (CMYI) were sourced from GL Biochem (Shanghai) Ltd. All these chemicals were used without further purification. 4-Succinimidylcarboxy-4'-methyl-2,2'-bipyridine (bpy-NHS),[2] methyl 4'-methyl-2,2'-bipyridine-4-carboxylate (bpy-COOMe),[3] 2-(1-naphthyl)-benzothiazole (Hbsn),[4] 1-(benzo[*b*]thiophen-2-yl)-isoquinoline (Hiqbt),[5] 6-(benzo[*b*]thiophen-2-yl)-phenanthridine (Hbtph),[6] and the iridium(III) dimers $[Ir_2(N^\wedge C)_4Cl_2]$ ($HN^\wedge C$ = Hpq, Hbsn, Hiqbt, Hbtph)[7] were prepared according to literature procedures. All buffer components were of biological grade and used as received. Autoclaved Milli-Q H_2O was used for the preparation of the aqueous solutions. Dulbecco's modified Eagle's medium (DMEM), fetal bovine serum (FBS), phosphate buffer saline (PBS) at pH 7.4, trypsin-EDTA, penicillin/streptomycin, LysoTracker Deep Red, and MitoTracker Green were purchased from

Invitrogen. HeLa, MDA-MB-231, MCF-7, and HEK-293 cells were obtained from American Type Culture Collection.

S-Benzyl 4'-methyl-2,2'-bipyridine-4-carbothioate (bpy-COSBn)

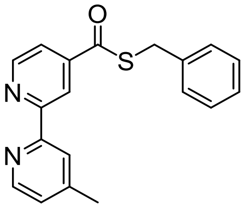

A mixture of bpy-NHS[2] (200 mg, 0.64 mmol), benzyl mercaptan (160 mg, 1.29 mmol), DIPEA (557 μL, 3.20 mmol), and DMAP (158 mg, 1.29 mmol) in dry THF was stirred at 298 K under an inert atmosphere of N_2 in the dark for 18 h. The solvent was removed under reduced pressure and the off-white crude product was purified by column chromatography on silica gel using CH_2Cl_2/CH_3CN (50:1, *v*/*v*) as the eluent. The product was subsequently isolated as a white solid. Yield: 179 mg (87%). ^1H NMR (300 MHz, $(CD_3)_2CO$, 298 K, TMS): δ 8.93 (dd, *J* = 1.7, 0.7 Hz, 1H, H3 of bpy), 8.88 (dd, *J* = 5.0, 0.7 Hz, 1H, H6 of bpy), 8.56 (d, *J* = 4.9 Hz, 1H, H6' of bpy), 8.33 (s, 1H, H3' of bpy), 7.83 (dd, *J* = 5.0, 3.6 Hz, 1H, H4 of benzyl ring), 7.49 – 7.44 (m, 2H, H5 and H5' of bpy), 7.39 – 7.26 (m, 4H, H2, H3, H5, and H6 of benzyl ring), 4.44 (s, 2H, CH_2 of benzyl ring), 2.46 (s, 3H, CH_3 of bpy). IR (KBr) $\tilde{\nu}$/cm^{-1}: 1707 (C=O). MS (ESI, positive mode, *m*/*z*): 321.3 $[M + H^+]^+$.

[Ir(pq)$_2$(bpy-COSBn)](PF$_6$) (**1a**)

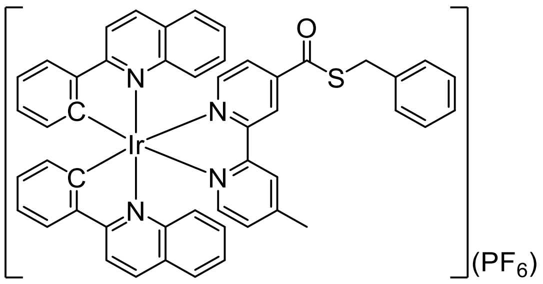

A mixture of [Ir$_2$(pq)$_4$Cl$_2$] (64 mg, 0.07 mmol) and bpy-COSBn (45 mg, 0.14 mmol) in CH$_2$Cl$_2$/MeOH (20 mL) (1:1, *v*/*v*) was stirred at 298 K under an inert atmosphere of N$_2$ in the dark for 18 h. The mixture was further stirred for 2 h after addition of solid KPF$_6$ (65 mg, 0.35 mmol). The solvent was removed under reduced pressure and the crude product was purified by column chromatography on silica gel using CH$_2$Cl$_2$/MeOH (100:1, *v*/*v*) as the eluent. The solvent was removed under reduced pressure to give an orange solid. Subsequent recrystallization of the solid from CH$_2$Cl$_2$/Et$_2$O afforded the complex as orange crystals. Yield: 101 mg (60%). ^{1}H NMR (300 MHz, (CD$_3$)$_2$CO, 298 K, TMS): δ 8.64 (d, *J* = 1.3 Hz, 1H, H3 of bpy), 8.61 – 8.50 (m, 6H, H3' and H6 of bpy and H3 and H4 of quinolinyl ring of pq), 8.25 (d, *J* = 7.2 Hz, 2H, H3 of phenyl ring of pq), 8.20 (d, *J* = 5.7 Hz, 1H, H6' of bpy), 8.07 (dd, *J* = 5.7, 1.8 Hz, 1H, H5 of bpy), 7.94 (dd, *J* = 8.1, 1.1 Hz, 1H, H8 of quinolinyl ring of pq), 7.90 (dd, *J* = 8.1, 1.1 Hz, 1H, H8 of quinolinyl ring of pq), 7.59 (d, *J* = 4.9 Hz, 1H, H5' of bpy), 7.47 – 7.28 (m, 9H, H7 and H6 of quinolinyl ring of pq and H2, H3, H4, H5, and H6 of benzyl ring), 7.22 – 7.08 (m, 4H, H4 of phenyl ring and H5 of quinolinyl ring of pq), 6.87 – 6.79 (m, 2H, H5 of phenyl ring of pq), 6.54 (t, *J* = 7.6 Hz, 2H, H6 of phenyl ring of pq), 4.39 (s, 2H, CH$_2$ of benzyl ring), 2.50 (s, 3H, CH$_3$ of bpy). ^{13}C NMR (150 MHz, (CD$_3$)$_2$CO, 298 K, TMS): δ 188.5, 170.3, 170.2, 157.3, 154.5, 152.4, 150.7, 150.6, 149.6, 147.5, 147.4, 147.3, 146.0, 145.9, 144.6, 140.4, 136.7, 134.4, 134.3, 131.3, 131.0, 130.7, 129.5, 129.4, 129.3, 129.1, 128.7,

128.0, 127.9, 127.6, 127.5, 127.4, 126.9, 126.8, 125.6, 124.9, 124.7, 123.0, 120.2, 118.1, 118.0. IR (KBr) $\tilde{\nu}$/cm^{-1}: 1710 (C=O), 842 (PF_6^-). HR-MS (ESI, positive mode, *m*/*z*): $[M - PF_6^-]^+$ calcd for $IrC_{49}H_{36}N_4OS$ 921.2239, found 921.2213. Anal. calcd for $IrC_{49}H_{36}N_4OSPF_6{\cdot}H_2O$: C 54.29, H 3.53, N 5.17, found: C 54.57, H 3.61, N 5.03%.

[Ir(pq)$_2$(bpy-COOMe)](PF$_6$) (**1b**)

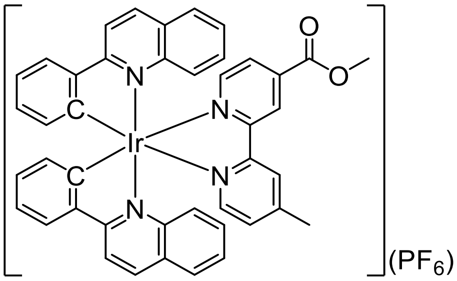

The synthetic procedure was similar to that for the preparation of complex **1a**, except that bpy-COOMe (32 mg, 0.14 mmol) was used instead of bpy-COSBn. Subsequent recrystallization of the solid from CH_2Cl_2/Et_2O afforded the complex as orange crystals. Yield: 85 mg (62%). ^{1}H NMR (300 MHz, $(CD_3)_2CO$, 298 K): δ 8.75 (s, 1H, H3 of bpy), 8.56 – 8.51 (m, 6H, H3' and H6 of bpy and H3 and H4 of quinolinyl ring of pq), 8.25 (d, J = 7.9 Hz, 2H, H3 of phenyl ring of pq), 8.20 (d, J = 5.7 Hz, 1H, H6' of bpy), 8.09 (dd, J = 5.7, 1.5 Hz, 1H, H5 of bpy), 7.93 (t, J = 8.0 Hz, 2H, H8 of quinolinyl ring of pq), 7.59 (d, J = 5.7 Hz, 1H, H5' of bpy), 7.48 – 7.38 (m, 4H, H7 and H6 of quinolinyl ring of pq), 7.22 – 7.09 (m, 4H, H4 of phenyl ring and H5 of quinolinyl ring of pq), 6.84 (t, J = 7.5 Hz, 2H, H5 of phenyl ring of pq), 6.55 (t, J = 6.7 Hz, 2H, H6 of phenyl ring of pq), 3.92 (s, 3H, CH_3 of COOMe), 2.51 (s, 3H, CH_3 of bpy). ^{13}C NMR (150 MHz, $(CD_3)_2CO$, 298 K): δ 170.3, 163.4, 157.0, 154.6, 152.4, 150.8, 150.6, 149.2, 147.5, 147.4, 147.3, 146.0, 145.9, 140.4, 139.9, 134.4, 134.3, 131.2, 131.0, 130.7, 129.4, 129.3, 128.0, 127.9, 127.5, 127.4, 127.1, 126.8, 125.5, 124.7, 123.0, 122.8, 118.1, 118.0, 65.2, 52.8. IR (KBr) $\tilde{\nu}$/cm^{-1}: 1738 (C=O), 846 (PF_6^-). HR-MS (ESI, positive mode, m/z): $[M - PF_6^-]^+$ calcd for $IrC_{43}H_{32}N_4O_2$ 829.2155, found 829.2131.

[Ir(bsn)$_2$(bpy-COSBn)](PF$_6$) (**2a**)

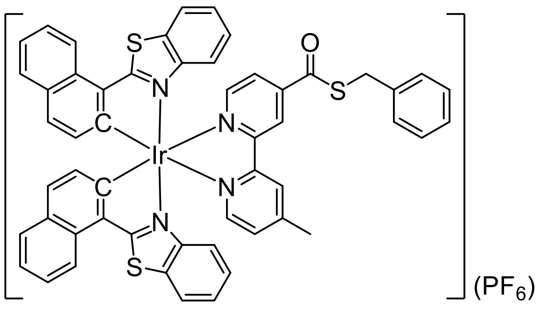

The synthetic procedure was similar to that for the preparation of complex **1a**, except that [Ir$_2$(bsn)$_4$Cl$_2$] (105 mg, 0.07 mmol) was used instead of [Ir$_2$(pq)$_4$Cl$_2$]. Subsequent recrystallization of the solid from CH_2Cl_2/Et_2O afforded the complex as dark orange crystals. Yield: 88 mg (56%). ^{1}H NMR (300 MHz, $(CD_3)_2CO$, 298 K): δ 9.00 (d, *J* = 1.2 Hz, 1H, H3 of bpy), 8.98 (s, 1H, H3' of bpy), 8.72 (d, *J* = 8.5 Hz, 2H, H7 of benzothiazole ring of bsn), 8.51 (d, *J* = 5.7 Hz, 1H, H6 of bpy), 8.32 – 8.24 (m, 2H, H3 of naphthyl ring of bsn), 8.14 (d, *J* = 5.6 Hz, 1H, H6' of bpy), 8.09 (dd, *J* = 5.8, 1.7 Hz, 1H, H5 of bpy), 7.86 (d, *J* = 8.1 Hz, 2H, H4 of benzothiazole ring of bsn), 7.83 – 7.75 (m, 2H, H6 of benzothiazole ring of bsn), 7.63 – 7.28 (m, 12H, H5' of bpy, H5 of benzothiazole ring and H4 and H7 of naphthyl ring of bsn, and H2, H3, H4, H5, and H6 of benzyl ring), 7.25 – 7.17 (m, 2H, H5 of naphthyl ring of bsn), 6.69 (d, *J* = 8.4 Hz, 1H, H8 of naphthyl ring of bsn), 6.66 (d, *J* = 8.4 Hz, 1H, H8 of naphthyl ring of bsn), 6.59 (d, *J* = 8.5 Hz, 1H, H6 of naphthyl ring of bsn), 6.48 (d, *J* = 8.5 Hz, 1H, H6 of naphthyl ring of bsn), 4.43 (s, 2H, CH_2 of benzyl ring), 2.62 (s, 3H, CH_3 of bpy). ^{13}C NMR (150 MHz, $(CD_3)_2CO$, 298 K): δ 188.5, 179.1, 178.9, 159.9, 159.7, 158.0, 155.2, 153.3, 152.2, 150.1, 148.0, 147.9, 145.2, 136.8, 134.3, 134.0, 132.6, 131.9, 131.8, 131.5, 131.4, 131.2, 131.0, 130.9, 130.4, 130.1, 129.1, 128.7, 128.5, 128.3, 127.7, 126.3, 126.0, 125.9, 125.6, 124.5, 123.8, 123.7, 121.6, 120.8, 117.4, 117.3, 65.2. IR (KBr) $\tilde{\nu}$/cm^{-1}: 1707 (C=O), 840 (PF_6^-). HR-MS

(ESI, positive mode, *m*/*z*): $[M - PF_6^-]^+$ calcd for $IrC_{53}H_{36}N_4OS_3$ 1033.1680, found 1033.1640. Anal. calcd for $IrC_{53}H_{36}N_4OS_3PF_6 \cdot 2H_2O$: C 52.42, H 3.32, N 4.61, found: C 52.20, H 3.12, N 4.28%.

$[Ir(bsn)_2(bpy\text{-}COOMe)](PF_6)$ (**2b**)

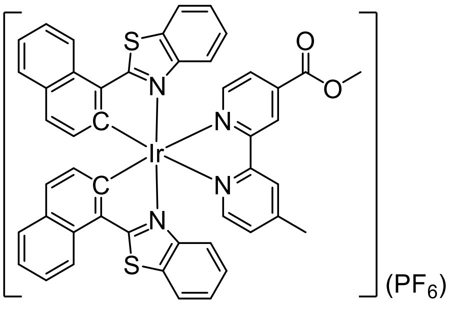

The synthetic procedure was similar to that for the preparation of complex **1b**, except that $[Ir_2(bsn)_4Cl_2]$ (105 mg, 0.07 mmol) was used instead of $[Ir_2(pq)_4Cl_2]$. Subsequent recrystallization of the solid from CH_2Cl_2/Et_2O afforded the complex as dark orange crystals. Yield: 90 mg (59%). ^{1}H NMR (300 MHz, $(CD_3)_2CO$, 298 K): δ 9.14 (s, 1H, H3 of bpy), 8.96 (s, 1H, H3' of bpy), 8.74 (d, J = 8.6 Hz, 2H, H7 of benzothiazole ring of bsn), 8.53 (d, J = 5.6 Hz, 1H, H6 of bpy), 8.31 (t, J = 7.1 Hz, 2H, H3 of naphthyl ring of bsn), 8.16 (d, J = 5.6 Hz, 1H, H6' of bpy), 8.13 (dd, J = 5.7, 1.4 Hz, 1H, H5 of bpy), 7.89 (d, J = 8.1 Hz, 2H, H4 of benzothiazole ring of bsn), 7.81 (t, J = 7.7 Hz, 2H, H6 of benzothiazole ring of bsn), 7.63 (d, J = 5.5 Hz, 1H, H5' of bpy), 7.59 – 7.49 (m, 4H, H5 of benzothiazole ring and H4 of naphthyl ring of bsn), 7.43 (dd, J = 8.4, 2.7 Hz, 2H, H7 of naphthyl ring of bsn), 7.25 (td, J = 7.9, 1.6 Hz, 2H, H5 of naphthyl ring of bsn), 6.71 (t, J = 7.9 Hz, 2H, H8 of naphthyl ring of bsn), 6.57 (d, J = 8.4 Hz, 1H, H6 of naphthyl ring of bsn), 6.51 (d, J = 8.5 Hz, 1H, H6 of naphthyl ring of bsn), 3.98 (s, 3H, CH_3 of COOMe), 2.65 (s, 3H, CH_3 of bpy). ^{13}C NMR (150 MHz, $(CD_3)_2CO$, 298 K): δ 179.1, 163.4, 159.8, 157.7, 155.3, 153.3, 151.9, 150.1, 148.0, 147.9, 140.7, 134.0, 132.6, 132.5, 131.8, 131.5, 131.4, 131.2, 131.0, 130.9, 130.4, 130.1, 128.7, 128.5, 128.3, 127.8, 126.2, 125.9, 124.5, 123.8, 123.7, 123.4, 121.6, 117.3, 117.2, 52.9. IR (KBr) $\tilde{\nu}$/cm^{-1}: 1743

(C=O), 840 (PF_6^-). HR-MS (ESI, positive mode, *m*/*z*): $[M - PF_6^-]^+$ calcd for $IrC_{47}H_{32}N_4O_2S_2$ 941.1596, found 941.1564.

[Ir(iqbt)$_2$(bpy-COSBn)](PF$_6$) (**3a**)

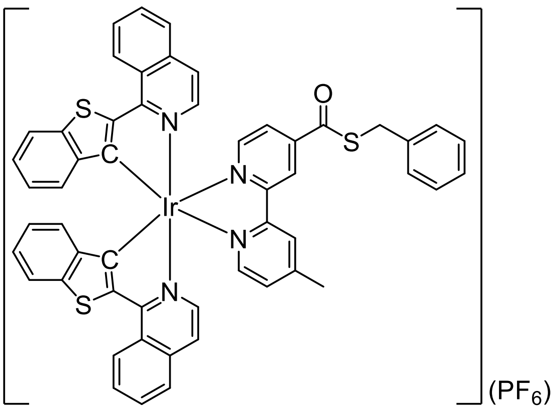

The synthetic procedure was similar to that for the preparation of complex **1a**, except that [Ir$_2$(iqbt)$_4$Cl$_2$] (105 mg, 0.07 mmol) was used instead of [Ir$_2$(pq)$_4$Cl$_2$]. Subsequent recrystallization of the solid from CH$_2$Cl$_2$/Et$_2$O afforded the complex as dark orange crystals. Yield: 85 mg (54%). ^{1}H NMR (400 MHz, (CD$_3$)$_2$CO, 298 K): δ 9.11 (dd, J = 8.0, 1.5 Hz, 2H, H8 of isoquinolinyl ring of iqbt), 9.06 (d, J = 1.0 Hz, 2H, H3 and H3' of bpy), 8.31 (d, J = 5.7 Hz, 1H, H6 of bpy), 8.12 – 8.07 (m, 2H, H6 of isoquinolinyl ring of iqbt), 8.06 – 7.98 (m, 3H, H5 of bpy and H7 of isoquinolinyl ring of iqbt), 7.98 – 7.92 (m, 5H, H5 of isoquinolinyl ring and H4 of benzothienyl ring of iqbt and H6' of bpy), 7.87 (d, J = 6.6 Hz, 1H, H3 of isoquinolinyl ring of iqbt), 7.77 (d, J = 6.5 Hz, 1H, H3 of isoquinolinyl ring of iqbt), 7.56 (dd, J = 5.6, 0.8 Hz, 1H, H5' of bpy), 7.50 (dd, J = 11.5, 6.5 Hz, 2H, H4 of isoquinolinyl ring of iqbt), 7.43 – 7.26 (m, 5H, H2, H3, H4, H5, and H6 of benzyl ring), 7.24 – 7.19 (m, 2H, H5 of benzothienyl ring of iqbt), 6.75 – 6.69 (m, 2H, H6 of benzothienyl ring of iqbt), 6.26 – 6.18 (m, 2H, H7 of benzothienyl ring of iqbt), 4.43 (s, 2H, CH$_2$ of benzyl ring), 2.60 (s, 3H, CH$_3$ of bpy). ^{13}C NMR (150 MHz, (CD$_3$)$_2$CO, 298 K): δ 188.7, 165.4, 165.3, 157.6, 155.6, 155.4, 154.6, 153.3, 152.8, 150.5, 145.3, 144.4, 144.3, 143.7, 143.6, 142.3, 142.0, 137.3, 136.8, 136.0, 135.7, 132.8, 130.4, 129.4, 129.0, 128.7, 127.8, 127.6, 126.8, 126.6, 126.5, 126.0, 125.8, 125.8,

125.0, 124.9, 124.4, 122.5, 121.2, 120.1. IR (KBr) $\tilde{\nu}$/cm^{-1}: 1714 (C=O), 840 (PF_6^-). HR-MS (ESI, positive mode, *m*/*z*): [M – PF_6^-]$^+$ calcd for $IrC_{53}H_{36}N_4OS_3$ 1033.1680, found 1033.1659. Anal. calcd for $IrC_{53}H_{36}N_4OS_3PF_6{\cdot}2H_2O$: C 52.42, H 3.32, N 4.61, found: C 52.20, H 3.12, N 4.28%.

[Ir(iqbt)$_2$(bpy-COOMe)](PF$_6$) (**3b**)

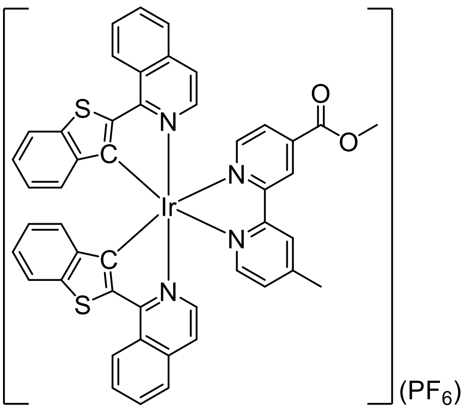

The synthetic procedure was similar to that for the preparation of complex **1b**, except that [Ir$_2$(iqbt)$_4$Cl$_2$] (100 mg, 0.07 mmol) was used instead of [Ir$_2$(pq)$_4$Cl$_2$]. Subsequent recrystallization of the solid from CH$_2$Cl$_2$/Et$_2$O afforded the complex as dark orange crystals. Yield: 84 mg (55%). ^{1}H NMR (300 MHz, (CD$_3$)$_2$CO, 298 K): δ 9.20 (d, J = 1.0 Hz, 1H, H3 of bpy), 9.14 (d, J = 8.5 Hz, 2H, H8 of isoquinolinyl ring of iqbt), 9.03 (s, 1H, H3' of bpy), 8.34 (dd, J = 5.3, 0.5 Hz, 1H, H6 of bpy), 8.15 – 7.95 (m, 10H, H5, H6, and H7 of isoquinolinyl ring and H4 of benzothienyl ring of iqbt and H5 and H6' of bpy), 7.85 (d, J = 6.5 Hz, 1H, H3 of isoquinolinyl ring of iqbt), 7.80 (d, J = 6.5 Hz, 1H, H3 of isoquinolinyl ring of iqbt), 7.58 (dd, J = 5.3, 0.8 Hz, 1H, H5' of bpy), 7.53 (t, J = 6.6 Hz, 2H, H4 of isoquinolinyl ring of iqbt), 7.24 (td, J = 7.3, 1.0 Hz, 2H, H5 of benzothienyl ring of iqbt), 6.74 (td, J = 7.4, 1.1 Hz, 2H, H6 of benzothienyl ring of iqbt), 6.25 (t, J = 7.7 Hz, 2H, H7 of benzothienyl ring of iqbt), 3.99 (s, 3H, CH$_3$ of COOMe), 2.63 (s, 3H, CH$_3$ of bpy). ^{13}C NMR (150 MHz, (CD$_3$)$_2$CO, 298 K): δ 165.4, 165.3, 163.5, 157.3, 155.7, 155.5, 154.7, 153.3, 152.5, 150.5, 144.4, 143.7, 143.6, 142.2, 141.9, 140.6, 137.3, 136.0, 135.7, 132.8, 130.4, 129.5, 128.2, 127.9, 126.8, 126.6, 126.5, 126.5, 125.9, 124.9, 124.4, 123.9, 122.5, 120.1, 52.9. IR (KBr) $\tilde{\nu}$/cm^{-1}: 1738 (C=O), 845 (PF$_6^-$). HR-MS (ESI, positive mode, m/z): [M – PF$_6^-$]$^+$ calcd for IrC$_{47}$H$_{32}$N$_4$O$_2$S$_2$ 941.1596, found 941.1564.

[Ir(btph)$_2$(bpy-COSBn)](PF$_6$) (**4a**)

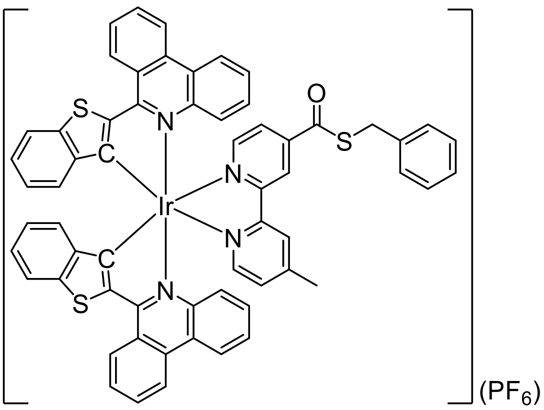

The synthetic procedure was similar to that for the preparation of complex **1a**, except that [Ir$_2$(btph)$_4$Cl$_2$] (100 mg, 0.06 mmol) was used instead of [Ir$_2$(pq)$_4$Cl$_2$]. Subsequent recrystallization of the solid from CH_2Cl_2/Et_2O afforded the complex as deep red crystals. Yield: 78 mg (52%). ^{1}H NMR (400 MHz, $(CD_3)_2CO$, 298 K): δ 9.50 – 9.44 (m, 2H, H10 of phenanthridinyl ring of btph), 9.17 (d, J = 5.9 Hz, 1H, H6 of bpy), 8.91 – 8.87 (m, 1H, H7 of phenanthridinyl ring of btph), 8.86 – 8.82 (m, 1H, H7 of phenanthridinyl ring of btph), 8.77 (d, J = 5.8 Hz, 1H, H6' of bpy), 8.57 (d, J = 8.4 Hz, 1H, H6 of phenanthridinyl ring of btph), 8.54 (d, J = 7.8 Hz, 1H, H6 of phenanthridinyl ring of btph), 8.42 (d, J = 1.4 Hz, 1H, H3 of bpy), 8.37 (s, 1H, H3' of bpy), 8.19 – 8.10 (m, 4H, H8 and H9 of phenanthridinyl ring of btph), 8.08 – 7.99 (m, 3H, H5 of bpy and H4 of benzothienyl ring of btph), 7.60 (d, J = 5.2 Hz, 1H, H5' of bpy), 7.46 – 7.27 (m, 9H, H3 and H5 of phenanthridinyl ring of btph and H2, H3, H4, H5, and H6 of benzyl ring), 7.23 – 7.17 (m, 2H, H5 of benzothienyl ring of btph), 6.92 – 6.80 (m, 4H, H4 of phenanthridinyl ring and H7 of benzothienyl ring of btph), 6.71 – 6.65 (m, 2H, H6 of benzothienyl ring of btph), 4.33 (s, 2H, CH_2 of benzyl ring), 2.37 (s, 3H, CH_3 of bpy). ^{13}C NMR (150 MHz, $(CD_3)_2CO$, 298 K): δ 159.4, 159.0, 157.4, 154.3, 153.6, 150.4, 148.2, 146.2, 146.1, 145.7, 144.3, 144.2, 139.5, 139.3, 137.0, 134.4, 134.3, 134.2, 129.8, 129.7, 129.6, 129.5,

129.1, 128.9, 128.6, 128.5, 128.1, 127.4, 127.3, 126.1, 125.7, 125.3, 125.1, 124.7, 123.6, 123.5, 123.4, 122.8, 120.2. IR (KBr) $\tilde{\nu}$/cm^{-1}: 1707 (C=O), 842 (PF_6^-). HR-MS (ESI, positive mode, *m*/*z*): $[M - PF_6^-]^+$ calcd for $IrC_{61}H_{40}N_4OS_3$ 1133.1993, found 1133.1973. Anal. calcd for $IrC_{61}H_{40}N_4OS_3PF_6{\cdot}H_2O$: C 56.52, H 3.27, N 4.32, found: C 56.74, H 3.07, N 4.08%.

[Ir(btph)$_2$(bpy-COOMe)](PF$_6$) (**4b**)

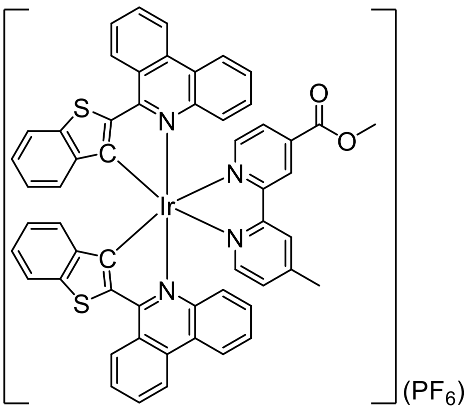

The synthetic procedure was similar to that for the preparation of complex **1b**, except that [Ir$_2$(btph)$_4$Cl$_2$] (100 mg, 0.06 mmol) was used instead of [Ir$_2$(pq)$_4$Cl$_2$]. Subsequent recrystallization of the solid from CH$_2$Cl$_2$/Et$_2$O afforded the complex as deep red crystals. Yield: 83 mg (50%). ^{1}H NMR (300 MHz, (CD$_3$)$_2$CO, 298 K): δ 9.52 – 9.47 (m, 2H, H10 of phenanthridinyl ring of btph), 9.18 (d, J = 5.9 Hz, 1H, H6 of bpy), 8.93 – 8.85 (m, 2H, H7 of phenanthridinyl ring of btph), 8.79 (d, J = 5.8 Hz, 1H, H6' of bpy), 8.62 – 8.53 (m, 3H, H6 of phenanthridinyl ring of btph and H3 of bpy), 8.34 (s, 1H, H3' of bpy), 8.21 – 8.13 (m, 4H, H8 and H9 of phenanthridinyl ring of btph), 8.11 (dd, J = 5.9, 1.7 Hz, 1H, H5 of bpy), 8.04 (d, J = 3.7 Hz, 1H, H4 of benzothienyl ring of btph), 8.02 (d, J = 3.7 Hz, 1H, H4 of benzothienyl ring of btph), 7.62 (dd, J = 5.9, 0.9 Hz, 1H, H5' of bpy), 7.49 – 7.37 (m, 4H, H3 and H5 of phenanthridinyl ring of btph), 7.22 (t, J = 7.6 Hz, 2H, H5 of benzothienyl ring of btph), 6.95 – 6.83 (m, 4H, H4 of phenanthridinyl ring and H7 of benzothienyl ring of btph), 6.74 – 6.66 (m, 2H, H6 of benzothienyl ring of btph), 3.87 (s, 3H, CH$_3$ of COOMe), 2.40 (s, 3H, CH$_3$ of bpy). ^{13}C NMR (150 MHz, (CD$_3$)$_2$CO, 298 K): δ 168.3, 163.1, 159.1, 158.6, 156.6, 154.0, 153.2, 149.6, 147.8, 145.8, 145.6, 143.9, 143.8, 143.7, 140.6, 139.1, 138.8, 134.0, 133.8, 129.3, 129.2, 128.6, 128.5, 128.1, 127.6, 126.9, 126.8, 125.7, 125.0, 124.8, 124.2, 123.2, 123.1, 122.9, 122.3,

52.8. IR (KBr) $\tilde{\nu}$/cm^{-1}: 1736 (C=O), 841 (PF_6^-). MS (ESI, positive mode, *m*/*z*): 1041.4 [M – PF_6^-]$^+$. HR-MS (ESI, positive mode, *m*/*z*): [M – PF_6^-]$^+$ calcd for $IrC_{55}H_{36}N_4O_2S_2$ 1041.1909, found 1041.1876.

Photophysical Studies

Electronic absorption spectra were recorded on an Agilent 8453 diode array spectrophotometer. Steady-state emission spectra were recorded on a HORIBA FluoroMax-4 spectrofluorometer. Unless specified otherwise, all solutions for photophysical studies were degassed with no fewer than four successive freeze-pump-thaw cycles and stored in a 10-cm^3 round bottomed flask equipped with a side-arm 1-cm fluorescence cuvette and sealed from the atmosphere by a Rotaflo HP6/6 quick-release Teflon stopper.

Emission quantum yields were measured by the optically dilute method[8] using an aerated aqueous solution of $[Ru(bpy)_3]Cl_2$ (Φ_{em} = 0.040, λ_{ex} = 455 nm)[9] as the standard solution. The concentrations of the standard and sample solutions were adjusted until the absorbance at 455 nm was ca. 0.1. The quantum yields of the sample were calculated according to the following equation, where the subscripts *s* and *r* refer to the sample and reference solutions, respectively:

$$\Phi_s = \Phi_r \left(\frac{I_r}{I_s}\right)\left(\frac{B_r}{B_s}\right)\left(\frac{n_s}{n_r}\right)^2\left(\frac{D_s}{D_r}\right)$$

where Φ is luminescence quantum yield, I is excitation intensity, B is $1 - 10^{-AL}$, A is absorbance at the excitation wavelength, L is path length in cm, n is refractive index of the solvent, and D is integrated intensity.

The emission lifetimes were measured on an Edinburgh Instruments LP920 laser flash photolysis spectrometer using the third harmonic output (355 nm; 6 – 8 ns fwhm pulse width) of a Spectra-Physics Quanta-Ray Q-switched LAB-150 pulsed Nd:YAG laser (10 Hz) as the excitation source. The lifetime τ_o was determined by a single exponential fitting of the luminescence decay trace with the relationship $I = I_o \exp(-t/\tau_o)$, where I_o and I are the luminescence intensities at time = 0 and t, respectively. Low-temperature alcohol glass

photophysical measurements were carried out with the sample in a quartz tube inside a quartz-walled Dewar filled with liquid nitrogen.

Determination of Singlet Oxygen ($^1O_2$) Generation Quantum Yields (Φ_Δ)

The $^1O_2$ generation quantum yields (Φ_Δ) were measured by the optically dilute method[8] using $[Ru(bpy)_3]Cl_2$ in aerated CH_3CN (Φ_Δ = 0.57) as a reference for $^1O_2$ sensitization.[10] An air-equilibrated CH_3CN solution (2 mL) containing the complex or conjugate was introduced to a quartz cuvette of 1-cm path length. The concentrations of the reference and sample solutions were adjusted until absorbance at the excitation wavelength (450 nm) was ca. 0.15. The solutions were excited at λ = 450 nm and the emission spectra of $^1O_2$ at 1200 – 1350 nm were recorded on an Edinburgh Instruments FLS980 spectrometer equipped with an R5509-73 NIR photomultiplier tube and C9940-02 exclusive coolers. The Φ_Δ value of the complex was determined using the following equation, where the subscripts *s* and *r* refer to the sample and reference solutions, respectively:

$$\Phi_s = \Phi_r \left(\frac{I_r}{I_s}\right)\left(\frac{B_r}{B_s}\right)\left(\frac{n_s}{n_r}\right)^2\left(\frac{D_s}{D_r}\right)$$

where Φ is luminescence quantum yield, *I* is excitation intensity, *B* is $1 - 10^{-AL}$, *A* is absorbance at the excitation wavelength, *L* is path length in cm, *n* is refractive index of the solvent, and *D* is integrated intensity.

Electrochemical Studies

Cyclic voltammetric measurements were performed on a CH Instruments Electrochemical Workstation CHI750A. Electrochemical measurements of the thioester complexes **1a** – **4a** and the ligand bpy-COSBn were performed in CH_3CN solutions (4 mL) with 0.1 M $^nBu_4NPF_6$ as supporting electrolyte at 298 K. A three-electrode system was employed, with a $Ag/AgNO_3$ (1

M KCl), a glassy carbon, and a platinum wire as the reference electrode, the working electrode, and the counter electrode, respectively, in a glass cell. The ferrocenium/ferrocene couple (Fc^+/Fc) was used as the internal reference. All solutions for electrochemical studies were deaerated with N_2 gas prior to measurements.

Kinetics Studies

All reactions were performed on a 100-μL scale. The reaction kinetics of the thioester complexes **1a** – **4a** (20 μM) and ligand bpy-COSBn (100 μM) with L-Cys (25 μM for the complexes and 250 μM for the ligand) in potassium phosphate buffer (50 mM, pH 7.0)/DMSO (3:2, *v*/*v*) containing TCEP (250 μM) at 298 K was measured by reversed-phase high-performance liquid chromatography (RP-HPLC). The reactions at different time points were quenched by the addition of 900 μL of cold H_2O/CH_3CN (1:1, *v*/*v*) and then analyzed by RP-HPLC. The HPLC analysis was carried out on an Agilent analytical column (ZORBAX Eclipse Plus C18 column: 4.6 × 150 mm, 5 μm) using H_2O containing 0.1% (*v*/*v*) TFA (solvent A) and CH_3CN containing 0.1% (*v*/*v*) TFA (solvent B) as the solvents, with a linear gradient of 20 – 60% solvent B over 12 min and a flow rate of 1 mL min^{-1} for the ligand, and 40 – 100% solvent B over 12 min and a flow rate of 1 mL min^{-1} for the complexes. The second-order rate constants (k_2) were determined by fitting the data to the following equation:

$$y = \frac{ln\frac{[A]_o[B]_t}{[A]_t[B]_o}}{([B]_o - [A]_o)} = k_2 t$$

where $[A]_o$ and $[A]_t$ are the concentrations of the thioester-containing compound (ligand or complex) at time = 0 and *t* s, respectively; and $[B]_o$ and $[B]_t$ are the concentrations of L-Cys at time = 0 and *t* s, respectively. All kinetics curves are generated using the OriginPro 8.0 software package.

Computational Studies

The ground state (S_0) structures of complexes **1a-Me**, **1a-Cys**, and **1b** were fully optimized by density functional theory (DFT) with the hybrid Perdew, Burke, and Ernzerhof (PBE0) functional,[11,12] in conjunction with the D3 version of Grimme's empirical dispersion with Becke-Johnson damping (D3BJ).[13] The Stuttgart/Dresden (SDD) effective core potentials (ECPs) and the associated basis set were used to describe iridium,[14] whereas other atoms were described using the 6-31G(d,p) basis set. The bulk solvent effect of the CH_2Cl_2 environment was simulated with the integral equation formalism of the polarizable continuum model (IEFPCM).[15] Unrestricted DFT (UDFT) method was used to optimize the lowest-lying triplet (T_1) state exhibiting metal-to-ligand charge-transfer (MLCT) and structurally distorted metal-centered (MC) characters. Only the ^{3}MC state associated with the dissociation of the Ir–N bond of the N^N ligand is depicted in Figure 4; however, it is noted that a close-lying ^{3}MC state associated with the dissociation of the Ir–N bond of the N^C ligands should also exist. An additional triplet distorted state (^{3}DS) featuring intraligand (IL) character localized on the thioester unit was identified for complex **1a-Me**. Vibrational frequency calculations were performed on the optimized geometries to verify that each was a minimum (NIMAG = 0) on the potential energy surface. All the structural optimization calculations were performed using the Gaussian 16 (Revision A.03) program suite.[16] The minimum energy crossing point (MECP) structures between ^{3}DS and S_0 states (i.e., ^{3}MC/S_0 and ^{3}IL/S_0 MECPs) were optimized by interfacing the sobMECP package[17] with Gaussian 16. Figure S26 shows the comparison of the geometric structures of the S_0 state, ^{3}MLCT state, ^{3}MC state, ^{3}MC/S_0 MECP, ^{3}IL state, and ^{3}IL/S_0 MECP for complexes **1a-Me**, **1a-Cys**, and **1b**. The root-mean-square deviations (Figure S26) and relative energies (Table S10) of these critical excited-state structures with respect to that of the S_0 state were also computed. As compared to the ^{3}MC state, less geometric distortion is required to access the ^{3}IL state localized on the thioester moiety of complex **1a-Me**. Cartesian

coordinates of all the optimized structures for complexes **1a-Me**, **1a-Cys**, and **1b** can be found in Tables S16 – S29. Figure S27 shows the schematic diagrams of potential energy profiles (eV) along the nuclear coordinate (Q) for complexes **1a-Me** and **1b**.

Rate constants for the ^{3}IL → S_0 phosphorescence (k_r^P) and the nonradiative decay through the ^{3}IL → S_0 intersystem crossing (k_{ISC}) process were evaluated to confirm that the ^{3}IL state of complex **1a-Me** is indeed nonemissive (Table S11). To account for large spin-orbit coupling (SOC) and relativistic effects induced by the iridium(III) center, SOC-coupled time-dependent DFT (SOC-TDDFT)[18,19] calculations with the zero-order regular approximation (ZORA)[20] Hamiltonian, in conjunction with segmented all-electron relativistically contracted (SARC) basis sets SARC-ZORA-TZVP[23] for iridium and ZORA-def2-TZVP[21,22] for all other atoms, were conducted using the ORCA (release 5.0.3) program package.[23] The solvent effect induced by the CH_2Cl_2 environment was simulated with the conductor-like polarizable continuum model (CPCM).[24,25] SOC-TDDFT/PBE0 calculations included 25 lowest spin pure singlet and triplet states, leading to a total of 100 SOC states. Due to the SOC effect, the ^{3}IL state is split into three sublevels. The radiative rate constant k_r^i from sublevel ^3IL_i (i = 1, 2, 3) to S_0 state is given by:[26,27]

$$k_{\mathrm{r}}^{i} = \frac{e^2}{2\pi\hbar^2 m_e c^3 \varepsilon_0} \Delta E^2_{\mathrm{S_0}-{}^3\mathrm{IL}_i} f_{\mathrm{S_0}-{}^3\mathrm{IL}_i}$$

where e and m_e are the charge and mass of an electron, respectively, c is the speed of light, ε_0 is the absolute dielectric permittivity of vacuum, and $\Delta E_{\mathrm{S_0}-{}^3\mathrm{IL}_i}$ and $f_{\mathrm{S_0}-{}^3\mathrm{IL}_i}$ are the transition energy and oscillator strength of the ^3IL_i → S_0 phosphorescence, respectively. SOC-TDDFT calculation was performed at the relaxed ^{3}IL structure to evaluate $\Delta E_{\mathrm{S_0}-{}^3\mathrm{IL}_i}$ and $f_{\mathrm{S_0}-{}^3\mathrm{IL}_i}$. Based on the Stickler-Berg relationship,[28] the radiative rate k_{r}^{i} was multiplied by the squared

refractive index of CH_2Cl_2 (n = 1.4244). Since the computed zero-field splitting is negligible (< 1 cm^{-1}), the overall observed ^{3}IL → S_0 phosphorescence rate constant is given by:

$$k_\mathrm{r}^\mathrm{P} = \frac{1}{3}\sum_{i=1}^{3} k_\mathrm{r}^{i}$$

Rate constant of the ^{3}IL→S_0 ISC was evaluated using the Marcus theory:

$$k_\mathrm{ISC} = \frac{2\pi}{\hbar}\frac{|H_\mathrm{SOC}|^2}{\sqrt{4\pi\lambda k_\mathrm{B}T}}\exp\left(-\frac{(\Delta E + \lambda)^2}{4\lambda k_\mathrm{B}T}\right)$$

where H_SOC is the SOC matrix element between ^{3}IL and S_0 states, k_B is the Boltzmann constant, T is the temperature, ΔE is the ^{3}IL → S_0 adiabatic energy gap, and $\lambda = (\lambda_\mathrm{i} + \lambda_\mathrm{o})$ is the total reorganization energy. The innersphere reorganization energy (λ_i) is defined as:[29]

$$\lambda_\mathrm{i} = E_{\mathrm{S}_0}^{3\mathrm{IL}} - E_{\mathrm{S}_0}^{\mathrm{S}_0}$$

where $E_{\mathrm{S}_0}^{3\mathrm{IL}}$ and $E_{\mathrm{S}_0}^{\mathrm{S}_0}$ are the energies of the S_0 state at the relaxed ^{3}IL and S_0 geometries, respectively. The outersphere solvent reorganization energy (λ_o) induced by the CH_2Cl_2 medium is typically small, and was set to 0.2 eV in this work.[30,31] H_SOC was computed at the ^{3}IL/S_0 MECP structure of complex **1a-Me**.

Table S1. Crystallographic data of complex **1a**.

**Formula**	**$C_{49}H_{36}F_6IrN_4OPS$**
Formula weight	1066.05
Space group	P-1
Crystal system	Triclinic
a/Å	13.0807(3)
b/Å	13.4228(3)
c/Å	15.7148(3)
α/°	79.726(2)
β/°	66.571(2)
γ/°	68.649(2)
Volume/Å^3	2356.13(10)
Z	2
Density/g cm^{-3}	1.503
*F*000	1056.0
Crystal size/mm^3	0.05 × 0.04 × 0.03
Radiation	Cu/Kα (λ = 1.54184 Å)
2θ range for data collection/°	6.134 to 151.322
Index ranges	$-16 \le h \le 16$, $-16 \le k \le 16$, $-19 \le l \le 18$
Reflections collected	28993
Independent reflections	9207
Data/restraints/parameters	9207/76/638
Goodness-of-fit on F^2	1.044

Final R indices ($I \geq 2\sigma(I)$)	R_1 = 0.0526, wR_2 = 0.1456
Final R indices (all data)	R_1 = 0.0548, wR_2 = 0.1477

Table S2. Selected bond lengths (Å) and bond angles (°) for complex **1a**.

Ir(1)–N(1)	2.094(5)	Ir(1)–N(2)	2.119(5)
Ir(1)–N(3)	2.179(4)	Ir(1)–N(4)	2.169(5)
Ir(1)–C(2)	2.000(6)	Ir(1)–C(15)	2.011(6)
N(1)–Ir(1)–N(2)	170.76(19)	N(1)–Ir(1)–N(3)	81.83(19)
N(1)–Ir(1)–N(4)	104.12(18)	N(1)–Ir(1)–C(2)	94.4(2)
N(1)–Ir(1)–C(15)	79.5(2)	N(2)–Ir(1)–N(3)	105.36(18)
N(2)–Ir(1)–N(4)	83.60(19)	N(2)–Ir(1)–C(2)	79.2(2)
N(2)–Ir(1)–C(15)	93.7(2)	N(3)–Ir(1)–N(4)	75.06(17)
N(3)–Ir(1)–C(2)	172.1(2)	N(3)–Ir(1)–C(15)	97.3(2)
N(4)–Ir(1)–C(2)	99.4(2)	N(4)–Ir(1)–C(15)	170.7(2)
C(2)–Ir(1)–C(15)	88.8(2)		

Table S3. Electronic absorption spectral data of the iridium(III) complexes at 298 K.

Complex	Solvent	λ_{abs}/nm (ε/dm^3 mol^{-1} cm^{-1})
**1a**	CH_2Cl_2	263 (57,475), 282 (58,505), 315 sh (30,715), 330 (29,930), 350 sh (25,170), 438 sh (6,890)
	CH_3CN	260 (58,255), 284 sh (52,980), 323 (28,990), 349 (23,660), 394 (7,770), 443 (5,625)
**1b**	CH_2Cl_2	262 (58,665), 282 (60,450), 324 (29,785), 350 sh (26,330), 396 sh (7,615), 436 (6,870)
	CH_3CN	259 (60,560), 280 (59,760), 321 (30,010), 348 sh (25,865), 399 sh (6,690), 433 (6,635)
**2a**	CH_2Cl_2	274 (59,310), 350 (37,375), 380 sh (25,220), 403 sh (16,980), 445 (12,525), 465 (12,230)
	CH_3CN	272 (53,185), 293 sh (37,585), 346 (33,665), 380 (19,020), 402 (13,575), 444 (9,880), 472 (8,745)
**2b**	CH_2Cl_2	275 (55,295), 323 (25,865), 351 (35,345), 379 sh (23,005), 402 (15,870), 446 (11,985), 464 (11,765)
	CH_3CN	272 (53,815), 319 (26,810), 346 (34,650), 382 sh (18,675), 403 (14,215), 444 (10,620), 479 sh (7,640)
**3a**	CH_2Cl_2	287 (42,045), 308 (47,490), 368 (26,220), 400 sh (19,450), 492 (13,815)
	CH_3CN	285 (41,210), 307 (47,510), 361 (26,655), 394 sh (18,850), 484 (12,440)
**3b**	CH_2Cl_2	290 (41,700), 308 (46,640), 323 sh (33,655), 369 (26,745), 407 sh (16,590), 492 (14,560)

	CH_3CN	285 (39,415), 308 (46,570), 323 sh (34,230), 362 (26,745), 399 sh (17,025), 482 (13,190)
**4a**	CH_2Cl_2	303 (31,505), 322 (26,345), 334 (26,605), 385 (21,250), 505 (9,925), 536 sh (8,070)
	CH_3CN	287 (32,605), 320 sh (25,335), 332 (25,805), 375 (19,895), 499 (9,310), 530 sh (7,400)
**4b**	CH_2Cl_2	307 (32,200), 323 (27,095), 334 (27,360), 383 (23,305), 423 sh (13,695), 504 (11,180), 535 (9,380)
	CH_3CN	288 (32,995), 306 sh (29,180), 321 (26,395), 334 (26,175), 374 (21,350), 405 sh (16,530), 499 (10,300), 529 (8,480)

Table S4. Photophysical data of the iridium(III) complexes.

Complex	Medium (*T*/K)	λ_{em}/nm[a]	τ_o/μs[b]	Φ_{em}[c]
**1a**	CH_2Cl_2 (298)	648	0.059	0.008
	CH_3CN (298)	562 sh, 657	0.41	0.002
	Buffer[d] (298)	560	0.35	0.004
	Glass[e] (77)	543 (max), 585	5.07	
**1b**	CH_2Cl_2 (298)	613	0.26	0.055
	CH_3CN (298)	625	0.10	0.015
	Glass[e] (77)	544 (max), 583	4.62	
**2a**	CH_2Cl_2 (298)	599 (max), 646, 710 sh	0.86	0.060
	CH_3CN (298)	597 (max), 647, 710 sh	0.36	0.025
	Buffer[d] (298)	596 (max), 646, 710 sh	0.36	0.014
	Glass[e] (77)	590 (max), 608, 640, 664 sh, 700	5.42	
**2b**	CH_2Cl_2 (298)	596 (max), 645, 703 sh	4.46	0.15
	CH_3CN (298)	595 (max), 646, 706 sh	3.79	0.13
	Glass[e] (77)	589 (max), 608, 641, 664 sh, 701	5.49	
**3a**	CH_2Cl_2 (298)	678, 735 sh	0.72	0.002
	CH_3CN (298)	674, 735 sh	1.33	0.002
	Buffer[d] (298)	676, 734 sh	1.69	0.002
	Glass[e] (77)	670 (max), 734	2.93	

**3b**	CH_2Cl_2 (298)	679, 739 sh	0.68	0.007
	CH_3CN (298)	676, 742 sh	1.28	0.010
	Glass[e] (77)	670 (max), 735	2.88	
**4a**	CH_2Cl_2 (298)	702, 783 sh	1.63	0.025
	CH_3CN (298)	702, 783 sh	1.02	0.013
	Buffer[d] (298)	701, 783 sh	1.09	0.012
	Glass[e] (77)	695 (max), 766	3.17	
**4b**	CH_2Cl_2 (298)	704, 778 sh	2.48	0.029
	CH_3CN (298)	704, 781 sh	1.55	0.021
	Glass[e] (77)	693 (max), 765	3.22	

[a] λ_{ex} = 350 nm.

[b] The lifetimes were measured at the emission maxima.

[c] The emission quantum yields were determined using $[Ru(bpy)_3]Cl_2$ (Φ_{em} = 0.040 in aerated H_2O, λ_{ex} = 455 nm)[9] as a reference.

[d] Potassium phosphate buffer (50 mM, pH 7.4)/MeOH (1:1, *v*/*v*).

[e] EtOH/MeOH (4:1, *v*/*v*).

Table S5. $^1O_2$ generation quantum yields (Φ_Δ) of the iridium(III) complexes in aerated CH_3CN at 298 K.

Complex	$\Phi_\Delta{}^a$
**1a**	0.33
**1b**	0.51
**2a**	0.60
**2b**	0.82
**3a**	0.13
**3b**	0.82
**4a**	0.80
**4b**	0.98

^{a} λ_{ex} = 450 nm and $[Ru(bpy)_3]Cl_2$ in aerated CH_3CN (Φ_Δ = 0.57) was adopted as the reference.[10]

Table S6. Electrochemical data of the thioester complexes **1a** – **4a** and ligand bpy-COSBn.[a]

Compound	Oxidation, $E_{1/2}$/V versus SCE[b] (ΔE_p/mV)	Reduction, $E_{1/2}$/V versus SCE[b] (ΔE_p/mV) [E_{pc}/V versus SCE][c]
**1a**	+1.32 (72)	–0.99 (59), [–1.65], –1.75 (69), –1.99 (75)
**2a**	+1.38 (78)	–0.93 (61), [–1.59], –1.67 (68), [–2.00]
**3a**	+1.12 (57), +1.49 (80)	–0.92 (61), [–1.56], –1.64 (77), –1.87 (66)
**4a**	+1.16 (67), [+1.63]	–0.94 (61), [–1.44], [–1.68], –1.81 (56)
bpy-COSBn		–1.00 (99)

[a] In CH_3CN solution with 0.1 M $^nBu_4NPF_6$ as supporting electrolyte at 298 K; scan rate = 100 mV s^{-1}; $E^o(Fc^+/Fc)$ = +0.40 V versus SCE.

[b] $E_{1/2} = (E_{pa} + E_{pc})/2$; $\Delta E_p = E_{pa} - E_{pc}$; E_{pa} and E_{pc} are peak anodic and peak cathodic potentials, respectively.

[c] Irreversible reduction wave.

Table S7. Photophysical data of complexes **1a** – **4a** (10 μM) before and after incubation with L-Cys (100 μM) in aerated potassium phosphate buffer (50 mM, pH 7.0)/CH_3CN (3:2, *v*/*v*) containing TCEP (1 mM) at 298 K for 1 h.

	Complex only		Complex + L-Cys (100 μM)		
Complex	λ_{em}/nm	τ/μs	λ_{em}/nm	I/I_o[a]	τ/μs
**1a**	556	0.26	606	14.3	0.04
**2a**	595	0.51	597	31.8	0.54
**3a**	676	0.23	676	17.0	0.35
**4a**	702	0.33	702	10.7	0.49

[a] I_o and I are the emission intensities of the complexes (10 μM) in the absence and presence of L-Cys, respectively.

Table S8. Photophysical data of the iridium(III) Cys and peptide conjugates in degassed solvents at 298 K.

Conjugate	Medium (*T*/K)	λ_{em}/nm[a]	τ_o/μs[b]	Φ_{em}[c]
**1a-Cys**	CH_3CN	554 sh, 596	0.21	0.055
	Buffer[d]	554 sh, 609	0.075	0.010
**2a-Cys**	CH_3CN	596 (max), 647, 708 sh	3.78	0.12
	Buffer[d]	595 (max), 647, 708 sh	3.41	0.12
**3a-Cys**	CH_3CN	675, 732 sh	1.92	0.016
	Buffer[d]	675, 732 sh	1.38	0.009
**4a-Cys**	CH_3CN	703, 777 sh	2.21	0.021
	Buffer[d]	701, 777 sh	1.77	0.017
**3a-CASP**	Buffer[d]	675, 732 sh	0.70	0.006
**3a-CYNT**	Buffer[d]	679, 732 sh	0.86	0.007
**3a-CMYI**	Buffer[d]	676, 732 sh	0.86	0.006

[a] λ_{ex} = 350 nm.

[b] The lifetimes were measured at the emission maxima.

[c] The emission quantum yields were determined using $[Ru(bpy)_3]Cl_2$ (Φ_{em} = 0.040 in aerated H_2O, λ_{ex} = 455 nm)[9] as a reference.

[d] Potassium phosphate buffer (50 mM, pH 7.4)/MeOH (2:3, *v*/*v*).

Table S9. $^1O_2$ generation quantum yields (Φ_Δ) of the iridium(III)–Cys conjugates in aerated CH_3CN at 298 K.

Conjugate	Φ_Δ [a]
**1a-Cys**	0.53
**2a-Cys**	0.77
**3a-Cys**	0.92
**4a-Cys**	0.98

[a] λ_{ex} = 450 nm and $[Ru(bpy)_3]Cl_2$ in aerated CH_3CN (Φ_Δ = 0.57) was adopted as the reference.[10]

Table S10. Relative energies (eV) of the structures at the relaxed ^{3}MLCT state, relaxed ^{3}MC state, ^{3}MC/S_0 MECP, relaxed ^{3}IL state, and ^{3}IL/S_0 MECP with respect to that of the ground state (S_0).

	**1a-Me**		**1a-Cys**		**1b**	
	S_0 surface	T_1 surface	S_0 surface	T_1 surface	S_0 surface	T_1 surface
S_0	0.00	2.34	0.00	2.48	0.00	2.44
^{3}MLCT	0.27	2.09	0.27	2.23	0.28	2.20
^{3}MC	2.48	2.82	2.42	2.80	2.50	2.83
^{3}MC/S_0 MECP	2.83	2.83	2.82	2.82	2.85	2.85
^{3}IL	1.36	2.60				
^{3}IL/S_0 MECP	2.84	2.84				

Table S11. H_{SOC} constants (cm^{-1}) between S_0 and ^{3}IL states computed at optimized ^{3}IL/S_0 MECP of **1a-Me**. The computed rate constants for $^3\mathrm{IL} \rightarrow S_0$ ISC (k_{ISC}) and $^3\mathrm{IL} \rightarrow S_0$ phosphorescence ($k_{\mathrm{r}}^{\mathrm{P}}$) are provided. Here, i is the imaginary unit.

	$H_{\mathrm{SOC},x}$	$H_{\mathrm{SOC},y}$	$H_{\mathrm{SOC},z}$	$H_{\mathrm{SOC}}{}^{a}$
	$-20.93i$	$38.63i$	$11.69i$	26.25
k_{ISC}	$1.65 \times 10^{8}\ \mathrm{s}^{-1}$			
$k_{\mathrm{r}}^{\mathrm{P}}$	$3.41 \times 10^{2}\ \mathrm{s}^{-1}$			

^{a} $H_{\mathrm{SOC}} = \sqrt{\frac{1}{3}\left(\left|H_{\mathrm{SOC},x}\right|^2 + \left|H_{\mathrm{SOC},y}\right|^2 + \left|H_{\mathrm{SOC},z}\right|^2\right)}$.

Table S12. Emission enhancement factors (I/I_o) of complex **1a** (10 μM) upon incubation with thiols (100 μM) in aerated potassium phosphate buffer (50 mM, pH 7.0)/CH_3CN (3:2, *v*/*v*) containing TCEP (1 mM) at 298 K for 1 h.

Thiol	I/I_o
L-Cys	14.3
Ethanethiol	1.19
GSH	1.92

[a] I_o and I are the emission intensities of complex **1a** (10 μM) in the absence and presence of the thiol, respectively.

Table S13. Cellular uptake and (photo)cytotoxicity of complex **3a** in MDA-MB-231 and HEK-293 cells. PI is the ratio $IC_{50,dark}/IC_{50,light}$.[a]

Cell line	Amount of iridium/fmol[b]	$IC_{50,dark}$/μM	$IC_{50,light}$/μM	PI
MDA-MB-231	0.18 ± 0.01	> 50	0.57 ± 0.03	> 88
HEK-293	0.087 ± 0.008	> 50	1.7 ± 0.1	> 29

[a] The cells were first incubated with complex **3a** in the dark for 2 h, replaced with fresh medium, and then incubated in the dark or irradiated at 450 nm (light dosage =14.6 mW cm^{-2}) for 10 min, and subsequently incubated in the dark for 24 h.

[b] Amount of iridium associated with an average cell upon incubation with complex **3a** (10 μM) at 37°C for 2 h, as determined by ICP-MS.

Table S14. $^1O_2$ generation quantum yields (Φ_Δ) of the Cys and peptide conjugates of complex **3a** in aerated CH_3CN/H_2O (4:1, *v*/*v*) at 298 K.

Conjugate	Φ_Δ [a]
**3a-Cys**	0.26
**3a-CASP**	0.24
**3a-CYNT**	0.28
**3a-CMYI**	0.20

[a] λ_{ex} = 450 nm and $[Ru(bpy)_3]Cl_2$ in aerated CH_3CN (Φ_Δ = 0.57) was adopted as the reference.[12]

Table S15. Cellular uptake of the conjugates of complex **3a** in live cells.

Conjugate	Amount of iridium/fmol[a]		
	MDA-MB-231	MCF-7	HEK-293
**3a-Cys**	0.30 ± 0.01	0.13 ± 0.02	0.040 ± 0.001
**3a-CASP**	1.6 ± 0.1	0.10 ± 0.01	0.074 ± 0.007
**3a-CYNT**	1.8 ± 0.1	0.10 ± 0.02	0.10 ± 0.01
**3a-CMYI**	0.21 ± 0.01	0.074 ± 0.002	0.033 ± 0.002

[a] Amount of iridium associated with an average cell upon incubation with the conjugates (10 μM) at 37°C for 16 h, as determined by ICP-MS.

Table S16. Cartesian coordinates of the optimized S_0 structure of complex **1a-Me**.

**1**	Ir	0.903233	-0.321616	-0.209833	**42**	H	2.335234	-3.109427	0.493460
**2**	S	-6.399831	0.669020	-1.439525	**43**	C	-0.469646	3.204997	5.036250
**3**	N	0.466143	0.673829	1.692504	**44**	H	-1.386527	3.773467	4.869691
**4**	N	2.031415	1.418990	-0.498629	**45**	H	0.346157	3.902209	5.244204
**5**	O	-5.810122	1.581206	0.955477	**46**	H	-0.609771	2.591541	5.933045
**6**	N	-1.180759	0.321048	-0.366735	**47**	C	-0.649594	-2.851952	3.500260
**7**	N	-0.015747	-2.199150	-0.061556	**48**	H	-0.359938	-2.449580	4.466162
**8**	C	2.719321	-0.999876	0.253605	**49**	C	-1.004250	-3.969972	-1.357520
**9**	C	-0.142357	2.333281	3.865954	**50**	H	-1.162476	-4.413196	-2.332439
**10**	C	-1.708918	0.898745	0.733526	**51**	C	0.433133	-2.695204	-3.686383
**11**	C	-0.759222	1.223335	1.809625	**52**	H	-0.097561	-3.614716	-3.914240
**12**	C	-1.084934	2.053970	2.878141	**53**	C	-0.138436	3.897368	-2.130742
**13**	H	-2.069430	2.503714	2.936714	**54**	H	-1.095726	3.931049	-2.641847
**14**	C	-1.987008	-0.020030	-1.379737	**55**	C	-1.580530	-4.511898	-0.245719
**15**	H	-1.505782	-0.478901	-2.237182	**56**	H	-2.215001	-5.390358	-0.318263
**16**	C	-3.074559	1.145497	0.828417	**57**	C	1.120394	-2.040108	-4.696781
**17**	H	-3.513922	1.586968	1.714515	**58**	H	1.120415	-2.443203	-5.704502
**18**	C	-0.498251	-2.783419	1.085995	**59**	C	4.126382	2.469166	0.048122
**19**	C	1.121501	1.754249	3.720544	**60**	H	5.126732	2.364075	0.448434
**20**	H	1.903981	1.935104	4.450037	**61**	C	3.657662	3.690345	-0.340504
**21**	C	-3.353852	0.202964	-1.356464	**62**	H	4.274508	4.579489	-0.248690
**22**	H	-3.952909	-0.093133	-2.210376	**63**	C	-1.516872	-3.960510	3.436582
**23**	C	3.295036	1.327709	-0.044188	**64**	H	-1.909279	-4.396046	4.349620
**24**	C	-3.911432	0.799327	-0.226877	**65**	C	1.818543	-0.864459	-4.407436
**25**	C	1.128365	-0.972568	-2.080896	**66**	H	2.365328	-0.355738	-5.197087
**26**	C	3.723753	-0.008934	0.341077	**67**	C	1.851790	5.021528	-1.383167
**27**	C	-0.210581	-2.803427	-1.248689	**68**	H	2.465061	5.912631	-1.285748
**28**	C	1.571875	2.616158	-0.994410	**69**	C	5.027928	-0.326755	0.747478
**29**	C	-5.372226	1.087706	-0.063557	**70**	H	5.795006	0.439109	0.811921
**30**	C	-0.151116	-2.274985	2.354323	**71**	C	0.616049	5.077924	-1.981683
**31**	H	0.537085	-1.446254	2.408110	**72**	H	0.230213	6.019787	-2.357637
**32**	C	2.362664	3.796680	-0.897916	**73**	C	-1.838552	-4.499951	2.214293
**33**	C	1.383528	0.943509	2.630449	**74**	H	-2.479567	-5.373715	2.141582
**34**	H	2.354558	0.478772	2.492196	**75**	C	4.373032	-2.632494	0.960173
**35**	C	0.443162	-2.171154	-2.385329	**76**	H	4.627858	-3.662742	1.194614
**36**	C	-1.325536	-3.940996	1.022272	**77**	C	5.350954	-1.638892	1.058001
**37**	C	0.325579	2.694431	-1.648979	**78**	H	6.359721	-1.891788	1.368527
**38**	H	-0.238847	1.787133	-1.795816	**79**	C	-7.987303	1.185476	-0.756533
**39**	C	1.823911	-0.338410	-3.118306	**80**	H	-7.968864	2.252164	-0.531580
**40**	H	2.374298	0.576413	-2.919902	**81**	H	-8.735010	0.975706	-1.522159
**41**	C	3.076174	-2.318624	0.562186	**82**	H	-8.206911	0.618475	0.148532

Table S17. Cartesian coordinates of the optimized ^{3}MLCT structure of complex **1a-Me**.

**1**	Ir	0.930816	-0.301194	-0.262486	**42**	H	2.425150	-3.008773	0.546796
**2**	S	-6.396319	0.628387	-1.350559	**43**	C	-0.495334	2.645461	5.336970
**3**	N	0.495417	0.556053	1.715969	**44**	H	-1.536872	2.971918	5.348944
**4**	N	1.944150	1.529141	-0.454505	**45**	H	0.146103	3.524411	5.458357
**5**	O	-5.816596	1.523019	1.068834	**46**	H	-0.324415	2.001511	6.206003
**6**	N	-1.158774	0.226475	-0.323758	**47**	C	-0.558649	-3.081988	3.313142
**7**	N	0.095239	-2.229320	-0.201569	**48**	H	-0.310405	-2.711011	4.302545
**8**	C	2.752269	-0.893101	0.248562	**49**	C	-0.816583	-3.960946	-1.602163
**9**	C	-0.159398	1.919449	4.073020	**50**	H	-0.959075	-4.351853	-2.601194
**10**	C	-1.700108	0.768545	0.828917	**51**	C	0.523522	-2.459730	-3.848378
**11**	C	-0.768905	1.016020	1.904173	**52**	H	0.097273	-3.416192	-4.131842
**12**	C	-1.101814	1.699593	3.086109	**53**	C	-0.424534	3.999625	-1.793844
**13**	H	-2.110231	2.073755	3.219035	**54**	H	-1.424285	4.025940	-2.215720
**14**	C	-2.008082	-0.079471	-1.341749	**55**	C	-1.363517	-4.592787	-0.521451
**15**	H	-1.550329	-0.503399	-2.228439	**56**	H	-1.953947	-5.495580	-0.644729
**16**	C	-3.057566	1.025223	0.923598	**57**	C	1.112016	-1.662117	-4.826477
**17**	H	-3.482441	1.441828	1.829576	**58**	H	1.127883	-2.002795	-5.856665
**18**	C	-0.372381	-2.885377	0.910335	**59**	C	4.018027	2.636716	0.054183
**19**	C	1.144349	1.437131	3.843880	**60**	H	5.037924	2.556940	0.407564
**20**	H	1.930677	1.576929	4.577654	**61**	C	3.488252	3.852076	-0.276631
**21**	C	-3.353072	0.154543	-1.303524	**62**	H	4.079058	4.758878	-0.187875
**22**	H	-3.954944	-0.104018	-2.167346	**63**	C	-1.365249	-4.230206	3.179438
**23**	C	3.223863	1.474936	-0.043931	**64**	H	-1.748074	-4.729578	4.063205
**24**	C	-3.928949	0.735383	-0.129649	**65**	C	1.680254	-0.428158	-4.490955
**25**	C	1.084001	-0.774733	-2.166359	**66**	H	2.135843	0.183058	-5.263878
**26**	C	3.716643	0.146438	0.306473	**67**	C	1.585285	5.156901	-1.154448
**27**	C	-0.084940	-2.768570	-1.420100	**68**	H	2.177472	6.062130	-1.061018
**28**	C	1.403528	2.726856	-0.855362	**69**	C	5.039340	-0.131550	0.653416
**29**	C	-5.337765	1.031645	0.051351	**70**	H	5.790610	0.650599	0.679076
**30**	C	-0.071180	-2.423752	2.208329	**71**	C	0.308903	5.195962	-1.659232
**31**	H	0.559768	-1.557068	2.323715	**72**	H	-0.131108	6.138779	-1.966738
**32**	C	2.163588	3.930445	-0.755952	**73**	C	-1.643434	-4.722486	1.927981
**33**	C	1.420774	0.778028	2.668655	**74**	H	-2.240498	-5.620427	1.800913
**34**	H	2.412244	0.385815	2.472069	**75**	C	4.465251	-2.468882	0.925093
**35**	C	0.501413	-2.020506	-2.525360	**76**	H	4.765804	-3.483637	1.167504
**36**	C	-1.143756	-4.077848	0.773959	**77**	C	5.408438	-1.437331	0.963715
**37**	C	0.106286	2.791742	-1.405009	**78**	H	6.436249	-1.652697	1.237355
**38**	H	-0.462350	1.881325	-1.515321	**79**	C	-7.972247	1.164365	-0.654836
**39**	C	1.665759	0.014405	-3.175933	**80**	H	-7.944650	2.231316	-0.430946
**40**	H	2.110601	0.971750	-2.927113	**81**	H	-8.734001	0.960737	-1.408942
**41**	C	3.148201	-2.200510	0.574558	**82**	H	-8.191213	0.607922	0.257110

Table S18. Cartesian coordinates of the optimized ^{3}MC structure of complex **1a-Me**.

**1**	Ir	1.099657	0.084291	-0.430270	**42**	H	3.949695	-1.444393	-0.682777
**2**	S	-6.497214	-1.437106	-0.325791	**43**	C	-0.559468	1.815458	5.625036
**3**	N	0.435375	0.430382	1.672327	**44**	H	-1.639444	1.838760	5.783967
**4**	N	0.975889	2.153707	-0.604950	**45**	H	-0.148980	2.809210	5.819082
**5**	O	-5.449155	0.866993	0.387052	**46**	H	-0.127623	1.131424	6.364371
**6**	N	-1.321332	-1.878012	0.972865	**47**	C	3.153325	-2.686895	2.730632
**7**	N	1.290797	-1.969702	-0.353334	**48**	H	3.669986	-2.145539	3.517075
**8**	C	3.057405	0.523887	-0.676143	**49**	C	0.665610	-4.041975	-1.396371
**9**	C	-0.219324	1.352010	4.245273	**50**	H	0.173460	-4.543178	-2.220582
**10**	C	-1.723825	-0.700671	1.459756	**51**	C	-0.511740	-2.264967	-3.540152
**11**	C	-0.745638	0.105873	2.233412	**52**	H	-0.501142	-3.325985	-3.769894
**12**	C	-1.091026	0.555237	3.504876	**53**	C	-2.587507	2.957812	-0.220261
**13**	H	-2.053032	0.266193	3.915977	**54**	H	-3.539062	2.438069	-0.144232
**14**	C	-2.195735	-2.584335	0.256866	**55**	C	1.228531	-4.761864	-0.380493
**15**	H	-1.843708	-3.540888	-0.119498	**56**	H	1.194951	-5.847267	-0.382593
**16**	C	-3.008066	-0.196738	1.265714	**57**	C	-1.080704	-1.380126	-4.447949
**17**	H	-3.303523	0.763451	1.672888	**58**	H	-1.519862	-1.757506	-5.365953
**18**	C	1.897742	-2.664810	0.662717	**59**	C	2.234192	4.193890	-0.776259
**19**	C	0.997745	1.678197	3.642245	**60**	H	3.199193	4.670770	-0.893099
**20**	H	1.725970	2.300965	4.150769	**61**	C	1.103158	4.943683	-0.611702
**21**	C	-3.488576	-2.153267	-0.028754	**62**	H	1.152769	6.028406	-0.598882
**22**	H	-4.136105	-2.758701	-0.654026	**63**	C	3.095621	-4.094575	2.772963
**23**	C	2.153449	2.783675	-0.762949	**64**	H	3.557186	-4.631097	3.595443
**24**	C	-3.902923	-0.927743	0.487416	**65**	C	-1.074897	-0.006493	-4.186416
**25**	C	0.047439	-0.390888	-2.068232	**66**	H	-1.512988	0.681742	-4.903778
**26**	C	3.310238	1.904064	-0.885904	**67**	C	-1.352640	5.026110	-0.297944
**27**	C	0.691684	-2.630183	-1.358573	**68**	H	-1.307851	6.111124	-0.280787
**28**	C	-0.182256	2.881215	-0.474322	**69**	C	4.596398	2.350939	-1.205192
**29**	C	-5.241258	-0.319639	0.221712	**70**	H	4.795436	3.404329	-1.376051
**30**	C	2.573304	-1.985607	1.698633	**71**	C	-2.551947	4.366989	-0.174133
**31**	H	2.647159	-0.907296	1.658452	**72**	H	-3.473949	4.926414	-0.053873
**32**	C	-0.147663	4.304149	-0.457652	**73**	C	2.468347	-4.780461	1.761013
**33**	C	1.279394	1.207558	2.374261	**74**	H	2.427383	-5.865749	1.765534
**34**	H	2.214824	1.459632	1.883524	**75**	C	5.395864	0.071794	-1.138891
**35**	C	0.053060	-1.782972	-2.355147	**76**	H	6.211155	-0.638718	-1.242099
**36**	C	1.864735	-4.087752	0.687158	**77**	C	5.635186	1.435509	-1.327088
**37**	C	-1.428849	2.229799	-0.368448	**78**	H	6.632221	1.783194	-1.578256
**38**	H	-1.457468	1.148726	-0.433701	**79**	C	-7.838053	-0.250939	-0.549261
**39**	C	-0.512249	0.484426	-3.014214	**80**	H	-7.564218	0.486834	-1.304083
**40**	H	-0.513689	1.554238	-2.831157	**81**	H	-8.705413	-0.822670	-0.881128
**41**	C	4.119108	-0.381058	-0.821752	**82**	H	-8.056515	0.248322	0.395190

Table S19. Cartesian coordinates of the optimized $^3MC/S_0$ MECP structure of complex **1a-Me**.

**1**	Ir	1.113426	0.085897	-0.436538	**42**	H	3.980399	-1.431430	-0.646836
**2**	S	-6.505735	-1.439678	-0.329780	**43**	C	-0.554275	1.810382	5.612757
**3**	N	0.423080	0.431825	1.653008	**44**	H	-1.633693	1.834797	5.773769
**4**	N	0.990888	2.157162	-0.606419	**45**	H	-0.142529	2.804103	5.804696
**5**	O	-5.455623	0.863666	0.382043	**46**	H	-0.121254	1.126317	6.350876
**6**	N	-1.330760	-1.884304	0.968167	**47**	C	3.154750	-2.687213	2.730401
**7**	N	1.290367	-1.970646	-0.351263	**48**	H	3.669510	-2.145337	3.517690
**8**	C	3.081661	0.533341	-0.648140	**49**	C	0.651865	-4.042443	-1.386962
**9**	C	-0.218605	1.345452	4.232275	**50**	H	0.152671	-4.542991	-2.207321
**10**	C	-1.731676	-0.704481	1.450004	**51**	C	-0.521113	-2.263710	-3.528411
**11**	C	-0.753030	0.102863	2.220256	**52**	H	-0.492247	-3.322688	-3.765433
**12**	C	-1.092703	0.549348	3.494643	**53**	C	-2.574617	2.954271	-0.219825
**13**	H	-2.052301	0.259604	3.910423	**54**	H	-3.525046	2.432381	-0.144339
**14**	C	-2.205577	-2.590837	0.253343	**55**	C	1.229101	-4.762977	-0.379487
**15**	H	-1.854341	-3.548601	-0.120529	**56**	H	1.203571	-5.848501	-0.386532
**16**	C	-3.015591	-0.200233	1.256511	**57**	C	-1.077957	-1.379075	-4.443817
**17**	H	-3.311651	0.760160	1.663069	**58**	H	-1.488231	-1.754104	-5.375713
**18**	C	1.900578	-2.665997	0.662177	**59**	C	2.244929	4.199769	-0.771502
**19**	C	0.996381	1.672023	3.624744	**60**	H	3.209287	4.679432	-0.883846
**20**	H	1.727811	2.292397	4.131573	**61**	C	1.111279	4.947133	-0.613228
**21**	C	-3.497322	-2.158321	-0.034889	**62**	H	1.157501	6.032100	-0.603760
**22**	H	-4.144823	-2.764018	-0.659798	**63**	C	3.096717	-4.095044	2.773076
**23**	C	2.167672	2.789394	-0.757432	**64**	H	3.555523	-4.630982	3.597432
**24**	C	-3.910743	-0.931907	0.479532	**65**	C	-1.091601	-0.008543	-4.172048
**25**	C	-0.016536	-0.395234	-2.027991	**66**	H	-1.511036	0.682240	-4.897790
**26**	C	3.328075	1.911825	-0.873757	**67**	C	-1.344472	5.024577	-0.299888
**27**	C	0.673590	-2.630579	-1.345212	**68**	H	-1.301324	6.109670	-0.282441
**28**	C	-0.169219	2.882119	-0.474833	**69**	C	4.610155	2.361025	-1.206147
**29**	C	-5.248535	-0.323107	0.215585	**70**	H	4.805111	3.414332	-1.383220
**30**	C	2.576255	-1.986233	1.697451	**71**	C	-2.542445	4.363744	-0.175544
**31**	H	2.648894	-0.907815	1.656243	**72**	H	-3.465662	4.921021	-0.055512
**32**	C	-0.137936	4.304970	-0.459670	**73**	C	2.470913	-4.781403	1.760837
**33**	C	1.270851	1.207100	2.353612	**74**	H	2.428136	-5.866705	1.766399
**34**	H	2.202200	1.462309	1.857732	**75**	C	5.416226	0.084383	-1.135833
**35**	C	0.016841	-1.783471	-2.330448	**76**	H	6.231440	-0.625389	-1.246505
**36**	C	1.867307	-4.088871	0.686585	**77**	C	5.650016	1.447184	-1.334347
**37**	C	-1.414668	2.228379	-0.365743	**78**	H	6.643038	1.795834	-1.599508
**38**	H	-1.440217	1.147478	-0.427463	**79**	C	-7.843910	-0.250500	-0.551018
**39**	C	-0.567991	0.478631	-2.979557	**80**	H	-7.567902	0.487393	-1.304898
**40**	H	-0.586826	1.547040	-2.789911	**81**	H	-8.712897	-0.819522	-0.882956
**41**	C	4.144401	-0.368834	-0.797899	**82**	H	-8.059769	0.248339	0.394229

Table S20. Cartesian coordinates of the optimized ^{3}IL structure of complex **1a-Me**.

**1**	Ir	0.852032	-0.395789	-0.195180	**42**	H	1.928628	-3.319184	0.595359
**2**	S	-6.438160	1.564666	-1.369295	**43**	C	-0.051749	3.450099	4.922093
**3**	N	0.535158	0.697861	1.676733	**44**	H	-0.848217	4.165158	4.707044
**4**	N	2.187949	1.183723	-0.522289	**45**	H	0.866008	3.999631	5.146409
**5**	O	-5.739106	2.389299	0.762467	**46**	H	-0.329976	2.897822	5.826628
**6**	N	-1.126271	0.501782	-0.388658	**47**	C	-1.022496	-2.608264	3.567915
**7**	N	-0.298192	-2.136014	-0.004537	**48**	H	-0.686741	-2.220919	4.525055
**8**	C	2.569960	-1.280488	0.298423	**49**	C	-1.502819	-3.798044	-1.261645
**9**	C	0.155701	2.499571	3.785785	**50**	H	-1.713898	-4.241219	-2.226700
**10**	C	-1.590931	1.176619	0.694340	**51**	C	0.100738	-2.785690	-3.610263
**11**	C	-0.605913	1.410460	1.764937	**52**	H	-0.538980	-3.638761	-3.816017
**12**	C	-0.812873	2.317717	2.801378	**53**	C	0.344550	3.867908	-2.228487
**13**	H	-1.724379	2.903543	2.833937	**54**	H	-0.604728	4.008361	-2.736429
**14**	C	-1.982733	0.239793	-1.395985	**55**	C	-2.147858	-4.232707	-0.140737
**15**	H	-1.565436	-0.300540	-2.239508	**56**	H	-2.889540	-5.024393	-0.195092
**16**	C	-2.903407	1.595463	0.787979	**57**	C	0.868104	-2.250068	-4.633249
**17**	H	-3.260979	2.107589	1.672763	**58**	H	0.822456	-2.678445	-5.629504
**18**	C	-0.856935	-2.622827	1.153372	**59**	C	4.400257	1.977657	-0.003551
**19**	C	1.328063	1.746264	3.672766	**60**	H	5.379970	1.760040	0.402574
**20**	H	2.125698	1.844016	4.401744	**61**	C	4.090673	3.234983	-0.433544
**21**	C	-3.300402	0.628960	-1.390457	**62**	H	4.816015	4.040973	-0.370219
**22**	H	-3.934694	0.393634	-2.238324	**63**	C	-2.023581	-3.598985	3.527252
**23**	C	3.430947	0.947865	-0.062050	**64**	H	-2.470534	-3.957600	4.448695
**24**	C	-3.805195	1.332397	-0.271085	**65**	C	1.705620	-1.162047	-4.370792
**25**	C	0.999367	-1.119048	-2.046081	**66**	H	2.314051	-0.747085	-5.170252
**26**	C	3.690280	-0.420089	0.363355	**67**	C	2.466798	4.750601	-1.521510
**27**	C	-0.564485	-2.741587	-1.176682	**68**	H	3.189954	5.558133	-1.453149
**28**	C	1.882962	2.414337	-1.054657	**69**	C	4.944928	-0.885466	0.784018
**29**	C	-5.142207	1.756378	-0.173339	**70**	H	5.801276	-0.219429	0.831064
**30**	C	-0.451780	-2.129426	2.410600	**71**	C	1.245652	4.945737	-2.120834
**31**	H	0.335254	-1.392654	2.445730	**72**	H	0.982148	5.916761	-2.527214
**32**	C	2.818424	3.486826	-0.996374	**73**	C	-2.408358	-4.124383	2.317086
**33**	C	1.478544	0.871444	2.612568	**74**	H	-3.155101	-4.911306	2.263078
**34**	H	2.377867	0.275154	2.495137	**75**	C	4.009256	-3.085535	1.057977
**35**	C	0.170239	-2.230119	-2.324192	**76**	H	4.134635	-4.132429	1.322238
**36**	C	-1.825291	-3.666529	1.114158	**77**	C	5.102827	-2.218158	1.132524
**37**	C	0.652640	2.631256	-1.708658	**78**	H	6.072264	-2.585297	1.454281
**38**	H	-0.028192	1.801281	-1.814461	**79**	C	-7.227983	0.039938	-0.800947
**39**	C	1.771785	-0.605505	-3.096467	**80**	H	-7.481329	0.146134	0.255379
**40**	H	2.430457	0.239560	-2.918796	**81**	H	-8.140627	-0.082770	-1.386383
**41**	C	2.762070	-2.624521	0.645523	**82**	H	-6.557203	-0.807284	-0.950741

Table S21. Cartesian coordinates of the optimized ^{3}IL/S_0 MECP structure of complex **1a-Me**.

**1**	Ir	0.860799	-0.399560	-0.194460	**42**	H	1.940690	-3.319496	0.605289
**2**	S	-6.518112	1.617648	-1.273591	**43**	C	-0.075130	3.448701	4.908760
**3**	N	0.528441	0.689826	1.673960	**44**	H	-0.875782	4.157033	4.687061
**4**	N	2.194573	1.183815	-0.518360	**45**	H	0.838952	4.006971	5.126392
**5**	O	-5.861181	2.350582	0.665831	**46**	H	-0.349350	2.903889	5.818753
**6**	N	-1.114108	0.498821	-0.404722	**47**	C	-1.017008	-2.611825	3.569030
**7**	N	-0.290572	-2.138687	-0.003137	**48**	H	-0.680565	-2.225931	4.526591
**8**	C	2.578941	-1.280268	0.305532	**49**	C	-1.497854	-3.798278	-1.261075
**9**	C	0.137180	2.490371	3.779903	**50**	H	-1.709815	-4.240509	-2.226297
**10**	C	-1.596403	1.160423	0.681355	**51**	C	0.107327	-2.787210	-3.609387
**11**	C	-0.618161	1.393445	1.759866	**52**	H	-0.535509	-3.637890	-3.816359
**12**	C	-0.830403	2.299239	2.796658	**53**	C	0.351975	3.871003	-2.223136
**13**	H	-1.745546	2.879555	2.827674	**54**	H	-0.599437	4.013113	-2.726619
**14**	C	-1.962236	0.254598	-1.427861	**55**	C	-2.144485	-4.232089	-0.140613
**15**	H	-1.532725	-0.269926	-2.275442	**56**	H	-2.887729	-5.022297	-0.195379
**16**	C	-2.907072	1.573672	0.771245	**57**	C	0.875504	-2.252508	-4.632254
**17**	H	-3.268243	2.070943	1.662742	**58**	H	0.827953	-2.679101	-5.629126
**18**	C	-0.850371	-2.625381	1.154306	**59**	C	4.407175	1.978494	0.001013
**19**	C	1.313899	1.743282	3.670267	**60**	H	5.385959	1.761143	0.409402
**20**	H	2.111759	1.847582	4.397822	**61**	C	4.099635	3.234709	-0.433498
**21**	C	-3.276646	0.639058	-1.435383	**62**	H	4.825688	4.040045	-0.372224
**22**	H	-3.894611	0.420513	-2.299570	**63**	C	-2.022073	-3.598795	3.527555
**23**	C	3.437391	0.948720	-0.056596	**64**	H	-2.471631	-3.955283	4.448527
**24**	C	-3.808104	1.324705	-0.304591	**65**	C	1.717082	-1.167583	-4.368809
**25**	C	1.011675	-1.124989	-2.043426	**66**	H	2.326007	-0.753152	-5.168134
**26**	C	3.698327	-0.418468	0.370294	**67**	C	2.478367	4.749345	-1.524845
**27**	C	-0.557608	-2.743318	-1.175397	**68**	H	3.203943	5.555076	-1.459608
**28**	C	1.890533	2.414826	-1.051522	**69**	C	4.953971	-0.882305	0.790278
**29**	C	-5.122064	1.737712	-0.229750	**70**	H	5.809476	-0.214951	0.837889
**30**	C	-0.443730	-2.134837	2.412322	**71**	C	1.256408	4.946690	-2.121620
**31**	H	0.346454	-1.401241	2.448308	**72**	H	0.994475	5.917493	-2.529716
**32**	C	2.827804	3.486148	-0.997141	**73**	C	-2.407058	-4.123092	2.316999
**33**	C	1.469389	0.868515	2.611316	**74**	H	-3.156889	-4.907314	2.262191
**34**	H	2.371758	0.276951	2.493494	**75**	C	4.022203	-3.084022	1.063603
**35**	C	0.179023	-2.233566	-2.322574	**76**	H	4.149668	-4.130904	1.326563
**36**	C	-1.821433	-3.666818	1.114601	**77**	C	5.114799	-2.215019	1.136939
**37**	C	0.658744	2.634677	-1.701848	**78**	H	6.085328	-2.581106	1.456457
**38**	H	-0.026986	1.807806	-1.798919	**79**	C	-7.272145	0.068869	-0.728794
**39**	C	1.785400	-0.613226	-3.093755	**80**	H	-7.296719	0.064294	0.361349
**40**	H	2.446114	0.230100	-2.914765	**81**	H	-8.285710	0.038440	-1.130949
**41**	C	2.773935	-2.623979	0.653650	**82**	H	-6.680589	-0.767128	-1.104500

Table S22. Cartesian coordinates of the optimized S_0 structure of complex **1a-Cys**.

**1**	Ir	1.687263	-0.296116	-0.228146	**46**	C	0.419926	-2.687795	3.676774
**2**	N	1.359068	0.759245	1.660236	**47**	H	0.779004	-2.252164	4.604149
**3**	N	2.769830	1.442736	-0.665980	**48**	C	-0.269483	-3.992525	-1.097643
**4**	O	-4.969255	1.609624	1.280675	**49**	H	-0.494661	-4.472936	-2.041357
**5**	N	-0.422048	0.297074	-0.262387	**50**	C	1.015654	-2.817409	-3.565184
**6**	N	0.804058	-2.174885	0.057287	**51**	H	0.483492	-3.751493	-3.718119
**7**	C	3.538788	-0.935202	0.135893	**52**	C	0.448977	3.837101	-2.214289
**8**	C	0.851203	2.460438	3.826674	**53**	H	-0.552203	3.845687	-2.634162
**9**	C	-0.890298	0.868151	0.868187	**54**	C	-0.771372	-4.486820	0.071145
**10**	C	0.124565	1.262323	1.857906	**55**	H	-1.410726	-5.364762	0.076319
**11**	C	-0.152528	2.112361	2.925264	**56**	C	1.628804	-2.200774	-4.645216
**12**	H	-1.147943	2.523402	3.048374	**57**	H	1.569138	-2.647672	-5.632500
**13**	C	-1.287954	-0.087403	-1.207421	**58**	C	4.896248	2.525174	-0.348911
**14**	H	-0.853961	-0.525311	-2.100462	**59**	H	5.927272	2.442531	-0.029275
**15**	C	-2.254527	1.046278	1.070352	**60**	C	4.390274	3.723182	-0.761633
**16**	H	-2.644517	1.488566	1.978846	**61**	H	5.007989	4.616444	-0.770722
**17**	C	0.402436	-2.712825	1.257185	**62**	C	-0.456958	-3.790043	3.716069
**18**	C	2.123440	1.926860	3.599472	**63**	H	-0.788453	-4.186284	4.670241
**19**	H	2.951067	2.159846	4.261338	**64**	C	2.329780	-1.007139	-4.452472
**20**	C	-2.659052	0.064425	-1.074196	**65**	H	2.818129	-0.528074	-5.297077
**21**	H	-3.300404	-0.244150	-1.892018	**66**	C	2.494496	4.996637	-1.710868
**22**	C	4.067346	1.378780	-0.313046	**67**	H	3.110609	5.891082	-1.714427
**23**	C	-3.156637	0.631442	0.097943	**68**	C	5.870751	-0.224970	0.424673
**24**	C	1.794321	-1.021254	-2.082944	**69**	H	6.633061	0.547775	0.395853
**25**	C	4.536982	0.065069	0.101596	**70**	C	1.209654	5.022869	-2.197574
**26**	C	0.529970	-2.824817	-1.089181	**71**	H	0.787739	5.944283	-2.585237
**27**	C	2.261592	2.614042	-1.176216	**72**	C	-0.864340	-4.374606	2.540968
**28**	C	-4.613664	0.842590	0.390735	**73**	H	-1.513779	-5.245152	2.546907
**29**	C	0.840831	-2.159207	2.477633	**74**	C	5.255703	-2.521530	0.795772
**30**	H	1.537638	-1.335988	2.451135	**75**	H	5.537087	-3.537578	1.059971
**31**	C	3.051693	3.798576	-1.210197	**76**	C	6.228577	-1.518399	0.772801
**32**	C	2.334352	1.093700	2.515566	**77**	H	7.259944	-1.749713	1.019313
**33**	H	3.309591	0.661263	2.315832	**78**	N	-5.483989	0.149481	-0.371329
**34**	C	1.103244	-2.237052	-2.291341	**79**	H	-5.194694	-0.621862	-0.961471
**35**	C	-0.430819	-3.867124	1.295528	**80**	C	-6.897801	0.199833	-0.106225
**36**	C	0.959977	2.660236	-1.715893	**81**	H	-7.065862	0.145873	0.978796
**37**	H	0.386196	1.748120	-1.758317	**82**	C	-7.487911	1.526820	-0.614545
**38**	C	2.412934	-0.426055	-3.189834	**83**	C	-7.510471	-1.042152	-0.751128
**39**	H	2.964270	0.501459	-3.066873	**84**	H	-6.874017	2.330125	-0.206324
**40**	C	3.930182	-2.235295	0.480418	**85**	H	-7.454486	1.577459	-1.703857
**41**	H	3.193806	-3.032950	0.504402	**86**	S	-9.203864	1.801883	-0.039077
**42**	C	0.583886	3.363197	4.988705	**87**	O	-6.834646	-1.849357	-1.350223
**43**	H	-0.423970	3.780337	4.950552	**88**	H	-9.793338	1.814074	-1.244972
**44**	H	1.303624	4.186487	5.012583	**89**	O	-8.817182	-1.203137	-0.610325
**45**	H	0.691933	2.811917	5.928688	**90**	H	-9.212406	-0.420588	-0.167295

Table S23. Cartesian coordinates of the optimized ^{3}MLCT structure of complex **1a-Cys**.

**1**	Ir	1.713580	-0.274546	-0.272768	**46**	C	0.410616	-2.886198	3.498172
**2**	N	1.394111	0.648165	1.675994	**47**	H	0.728652	-2.482615	4.454298
**3**	N	2.740894	1.530035	-0.608232	**48**	C	-0.176357	-3.948517	-1.352244
**4**	O	-4.982311	1.328055	1.495268	**49**	H	-0.390475	-4.374425	-2.323892
**5**	N	-0.389162	0.248416	-0.245017	**50**	C	1.046950	-2.569153	-3.736843
**6**	N	0.856558	-2.185398	-0.082144	**51**	H	0.605103	-3.537164	-3.948210
**7**	C	3.555887	-0.876741	0.126780	**52**	C	0.301057	4.019245	-1.774482
**8**	C	0.839403	1.981519	4.077187	**53**	H	-0.742892	4.061857	-2.068854
**9**	C	-0.867151	0.746465	0.959646	**54**	C	-0.666278	-4.525621	-0.214837
**10**	C	0.115010	1.038003	1.957413	**55**	H	-1.280166	-5.419712	-0.264633
**11**	C	-0.161411	1.701451	3.172444	**56**	C	1.568932	-1.817411	-4.786138
**12**	H	-1.177371	2.013103	3.386857	**57**	H	1.518052	-2.203882	-5.798939
**13**	C	-1.287860	-0.081288	-1.204317	**58**	C	4.885666	2.596921	-0.373291
**14**	H	-0.870218	-0.443252	-2.137563	**59**	H	5.934575	2.501610	-0.124325
**15**	C	-2.241471	0.914325	1.151516	**60**	C	4.359139	3.809621	-0.716805
**16**	H	-2.628593	1.294804	2.090092	**61**	H	4.982242	4.698469	-0.745464
**17**	C	0.447515	-2.786961	1.082594	**62**	C	-0.428471	-4.018181	3.461415
**18**	C	2.155794	1.586315	3.741570	**63**	H	-0.766022	-4.472848	4.386833
**19**	H	2.987448	1.781221	4.409661	**64**	C	2.154230	-0.570126	-4.543782
**20**	C	-2.641289	0.059184	-1.061206	**65**	H	2.555223	0.006892	-5.371389
**21**	H	-3.278868	-0.179345	-1.904463	**66**	C	2.407369	5.132648	-1.445277
**22**	C	4.054277	1.457915	-0.326022	**67**	H	3.032810	6.019899	-1.469125
**23**	C	-3.154435	0.569371	0.173157	**68**	C	5.886861	-0.159773	0.298246
**24**	C	1.719798	-0.815390	-2.170914	**69**	H	6.656302	0.600877	0.219626
**25**	C	4.546570	0.136366	0.047451	**70**	C	1.081786	5.192638	-1.798343
**26**	C	0.588238	-2.766127	-1.264910	**71**	H	0.636843	6.134068	-2.103069
**27**	C	2.191607	2.727496	-0.999361	**72**	C	-0.796273	-4.551852	2.250640
**28**	C	-4.585712	0.737115	0.485161	**73**	H	-1.420543	-5.438726	2.197232
**29**	C	0.841414	-2.283015	2.339564	**74**	C	5.282619	-2.462383	0.745809
**30**	H	1.495712	-1.426461	2.380143	**75**	H	5.578577	-3.470630	1.019085
**31**	C	2.991959	3.907917	-1.050713	**76**	C	6.249904	-1.457069	0.648700
**32**	C	2.377287	0.939453	2.549678	**77**	H	7.291753	-1.686397	0.847796
**33**	H	3.372338	0.606863	2.275228	**78**	N	-5.466079	0.209598	-0.411631
**34**	C	1.109737	-2.070733	-2.435902	**79**	H	-5.173928	-0.475174	-1.096731
**35**	C	-0.356055	-3.965251	1.042663	**80**	C	-6.872572	0.206714	-0.123735
**36**	C	0.838543	2.813910	-1.386804	**81**	H	-7.028198	0.008052	0.946655
**37**	H	0.232749	1.920688	-1.368557	**82**	C	-7.467381	1.592570	-0.432947
**38**	C	2.227007	-0.070715	-3.250571	**83**	C	-7.500736	-0.934302	-0.917133
**39**	H	2.683155	0.897502	-3.074936	**84**	H	-6.859978	2.319017	0.107990
**40**	C	3.948015	-2.176579	0.489579	**85**	H	-7.410071	1.813134	-1.500080
**41**	H	3.206786	-2.965076	0.564674	**86**	S	-9.193020	1.799453	0.143774
**42**	C	0.564006	2.687451	5.367359	**87**	O	-6.849942	-1.651231	-1.644972
**43**	H	-0.496082	2.924058	5.478569	**88**	H	-9.776496	1.875945	-1.063039
**44**	H	1.135299	3.620029	5.426966	**89**	O	-8.804756	-1.121668	-0.756250
**45**	H	0.871911	2.069770	6.217832	**90**	H	-9.183618	-0.407653	-0.199680

Table S24. Cartesian coordinates of the optimized ^{3}MC structure of complex **1a-Cys**.

**1**	Ir	1.861173	0.076351	-0.462333	**46**	C	1.237853	-3.961144	-1.668565
**2**	N	1.322370	0.319157	1.691017	**47**	H	0.694253	-4.390329	-2.500889
**3**	N	1.790425	2.154529	-0.501367	**48**	C	0.053183	-2.009303	-3.650286
**4**	N	-0.573856	-1.811302	0.884915	**49**	H	0.017042	-3.052628	-3.948701
**5**	N	1.986054	-1.983721	-0.526630	**50**	C	-1.727152	3.018572	0.111987
**6**	C	3.818914	0.479877	-0.766274	**51**	H	-2.687210	2.516978	0.209655
**7**	C	0.870401	1.072313	4.361408	**52**	C	1.811295	-4.764777	-0.723892
**8**	C	-0.899541	-0.690024	1.534475	**53**	H	1.733926	-5.845871	-0.790939
**9**	C	0.163695	0.005353	2.301606	**54**	C	-0.516908	-1.048391	-4.476348
**10**	C	-0.079964	0.370678	3.623637	**55**	H	-1.002031	-1.350062	-5.399246
**11**	H	-1.026119	0.090156	4.074922	**56**	C	3.096612	4.168670	-0.602566
**12**	C	-1.523020	-2.406894	0.163930	**57**	H	4.068418	4.627184	-0.735333
**13**	H	-1.231453	-3.318216	-0.351183	**58**	C	1.995806	4.934551	-0.337032
**14**	C	-2.177627	-0.136024	1.501353	**59**	H	2.076647	6.014610	-0.258314
**15**	H	-2.407823	0.784968	2.024806	**60**	C	3.835107	-4.369597	2.377968
**16**	C	2.606922	-2.764495	0.415972	**61**	H	4.309170	-4.974227	3.144168
**17**	C	2.061046	1.395042	3.704500	**62**	C	-0.453424	0.304221	-4.126956
**18**	H	2.847354	1.947724	4.207724	**63**	H	-0.893242	1.052208	-4.780667
**19**	C	-2.819962	-1.915649	0.037176	**64**	C	-0.439191	5.057223	0.104595
**20**	H	-3.527009	-2.423451	-0.610203	**65**	H	-0.363427	6.137304	0.190305
**21**	C	2.977044	2.763024	-0.673158	**66**	C	5.386000	2.296217	-1.248257
**22**	C	-3.154603	-0.750845	0.723823	**67**	H	5.607855	3.352894	-1.361348
**23**	C	0.732754	-0.255407	-2.083801	**68**	C	-1.648467	4.420480	0.248009
**24**	C	4.102116	1.863491	-0.901721	**69**	H	-2.546942	4.993359	0.453574
**25**	C	1.321966	-2.556545	-1.544875	**70**	C	3.137497	-4.965619	1.355207
**26**	C	0.660273	2.900672	-0.269908	**71**	H	3.052112	-6.046626	1.294334
**27**	C	3.354051	-2.178686	1.459740	**72**	C	6.122157	-0.002681	-1.357287
**28**	H	3.470948	-1.103815	1.481724	**73**	H	6.911843	-0.726164	-1.539352
**29**	C	0.736033	4.318141	-0.162832	**74**	C	6.392250	1.363544	-1.471190
**30**	C	2.241666	1.009353	2.390875	**75**	H	7.387591	1.700370	-1.743105
**31**	H	3.155573	1.259650	1.860145	**76**	C	-4.486874	-0.075710	0.625442
**32**	C	0.678021	-1.625149	-2.459540	**77**	O	-4.598729	1.136082	0.806487
**33**	C	2.517663	-4.183936	0.354204	**78**	N	-5.535776	-0.865275	0.317806
**34**	C	-0.597629	2.275001	-0.145253	**79**	H	-5.479592	-1.876188	0.364331
**35**	H	-0.659173	1.201764	-0.280190	**80**	C	-6.871302	-0.332239	0.263709
**36**	C	0.168680	0.698116	-2.948314	**81**	H	-7.017657	0.363877	1.101403
**37**	H	0.210631	1.752887	-2.695938	**82**	C	-7.073491	0.449883	-1.046446
**38**	C	4.847283	-0.441350	-1.013477	**83**	C	-7.827154	-1.509330	0.442533
**39**	H	4.653480	-1.506622	-0.932618	**84**	H	-6.259132	1.171899	-1.116741
**40**	C	0.638702	1.451185	5.788744	**85**	H	-7.025228	-0.215388	-1.910155
**41**	H	-0.407283	1.323292	6.073124	**86**	S	-8.638403	1.397277	-1.085523
**42**	H	0.929907	2.489848	5.967851	**87**	O	-7.427475	-2.645044	0.571315
**43**	H	1.249630	0.824613	6.447698	**88**	O	-9.121231	-1.225064	0.466173
**44**	C	3.948877	-2.965302	2.419289	**89**	H	-9.244954	0.697935	-2.057668
**45**	H	4.521383	-2.494911	3.212652	**90**	H	-9.265753	-0.275041	0.266936

Table S25. Cartesian coordinates of the optimized ^{3}MC/S_0 MECP structure of complex **1a-Cys**.

**1**	Ir	1.885675	0.079275	-0.472967	**46**	C	1.218603	-3.960238	-1.648748
**2**	N	1.312257	0.314809	1.666259	**47**	H	0.655193	-4.389347	-2.468015
**3**	N	1.814531	2.159357	-0.503057	**48**	C	0.052957	-2.007157	-3.640255
**4**	N	-0.587331	-1.826939	0.870041	**49**	H	0.053548	-3.045783	-3.956799
**5**	N	1.997853	-1.983248	-0.527161	**50**	C	-1.703756	3.017296	0.122303
**6**	C	3.855868	0.489589	-0.742234	**51**	H	-2.662143	2.513104	0.223995
**7**	C	0.865678	1.064535	4.338148	**52**	C	1.804755	-4.763165	-0.711652
**8**	C	-0.907030	-0.700663	1.513631	**53**	H	1.721826	-5.844167	-0.773478
**9**	C	0.157116	-0.005101	2.279557	**54**	C	-0.527168	-1.049126	-4.462604
**10**	C	-0.083548	0.357979	3.603202	**55**	H	-0.982809	-1.348381	-5.401158
**11**	H	-1.027670	0.073319	4.056351	**56**	C	3.117089	4.175510	-0.599044
**12**	C	-1.541366	-2.421819	0.154782	**57**	H	4.088846	4.635291	-0.727780
**13**	H	-1.256042	-3.337937	-0.355241	**58**	C	2.014429	4.939871	-0.337248
**14**	C	-2.183170	-0.141671	1.480795	**59**	H	2.093398	6.020064	-0.258989
**15**	H	-2.407980	0.781560	2.002909	**60**	C	3.845023	-4.370110	2.378836
**16**	C	2.619832	-2.763508	0.415181	**61**	H	4.316884	-4.974567	3.146473
**17**	C	2.053140	1.391906	3.678011	**62**	C	-0.504469	0.297919	-4.091403
**18**	H	2.837608	1.949095	4.179382	**63**	H	-0.949001	1.045356	-4.743222
**19**	C	-2.836798	-1.925829	0.028594	**64**	C	-0.421814	5.058880	0.098638
**20**	H	-3.547170	-2.434282	-0.614301	**65**	H	-0.348562	6.139794	0.176718
**21**	C	3.000199	2.769725	-0.671662	**66**	C	5.406922	2.311251	-1.258078
**22**	C	-3.165688	-0.756472	0.710248	**67**	H	5.619383	3.367563	-1.389463
**23**	C	0.668596	-0.260496	-2.037519	**68**	C	-1.629555	4.420546	0.244785
**24**	C	4.129272	1.872929	-0.896225	**69**	H	-2.530349	4.992575	0.442713
**25**	C	1.312660	-2.555781	-1.530751	**70**	C	3.147962	-4.965173	1.355728
**26**	C	0.683649	2.903259	-0.265975	**71**	H	3.060695	-6.046100	1.295167
**27**	C	3.366764	-2.178405	1.459542	**72**	C	6.159260	0.017917	-1.343815
**28**	H	3.482105	-1.103402	1.484093	**73**	H	6.952121	-0.701928	-1.524028
**29**	C	0.755665	4.321217	-0.164068	**74**	C	6.418819	1.383764	-1.477112
**30**	C	2.232182	1.006485	2.364871	**75**	H	7.409337	1.724401	-1.761689
**31**	H	3.144394	1.259875	1.832378	**76**	C	-4.497514	-0.078703	0.615063
**32**	C	0.650759	-1.624853	-2.434650	**77**	O	-4.605908	1.133964	0.792190
**33**	C	2.525858	-4.182904	0.356429	**78**	N	-5.549517	-0.867308	0.314389
**34**	C	-0.571943	2.275381	-0.129065	**79**	H	-5.494670	-1.878008	0.361533
**35**	H	-0.630094	1.200817	-0.250674	**80**	C	-6.884858	-0.332491	0.260952
**36**	C	0.090394	0.688844	-2.896733	**81**	H	-7.030733	0.363409	1.098832
**37**	H	0.105472	1.741428	-2.633385	**82**	C	-7.086679	0.450109	-1.049091
**38**	C	4.889242	-0.425784	-0.985949	**83**	C	-7.842814	-1.508215	0.439760
**39**	H	4.704459	-1.492007	-0.895464	**84**	H	-6.272716	1.172614	-1.118168
**40**	C	0.641682	1.442030	5.767169	**85**	H	-7.037408	-0.214694	-1.913096
**41**	H	-0.401877	1.314953	6.060638	**86**	S	-8.651822	1.397146	-1.089540
**42**	H	0.935618	2.479966	5.945700	**87**	O	-7.445315	-2.644706	0.568108
**43**	H	1.257947	0.813940	6.419675	**88**	O	-9.136476	-1.221752	0.464144
**44**	C	3.957932	-2.965532	2.420813	**89**	H	-9.257580	0.697646	-2.062110
**45**	H	4.525711	-2.495332	3.217747	**90**	H	-9.279978	-0.271705	0.264016

Table S26. Cartesian coordinates of the optimized S_0 structure of complex **1b**.

**1**	Ir	0.747779	-0.359914	-0.190317	**42**	C	-0.618700	3.369943	4.913412
**2**	N	0.289484	0.699777	1.671739	**43**	H	-1.505489	3.971720	4.706199
**3**	N	1.992432	1.303190	-0.455914	**44**	H	0.221338	4.038532	5.119975
**4**	O	-5.906759	2.039694	0.546229	**45**	H	-0.805551	2.792291	5.825228
**5**	N	-1.282506	0.408125	-0.454840	**46**	C	-1.130424	-2.716655	3.484636
**6**	N	-0.290145	-2.175205	-0.053156	**47**	H	-0.864272	-2.311665	4.456190
**7**	C	2.496284	-1.136669	0.368834	**48**	C	-1.322404	-3.909542	-1.364599
**8**	C	-0.300975	2.448498	3.779017	**49**	H	-1.462965	-4.359838	-2.339035
**9**	C	-1.817659	1.051546	0.604955	**50**	C	0.304741	-2.777159	-3.640603
**10**	C	-0.897916	1.336147	1.717773	**51**	H	-0.264331	-3.671741	-3.875237
**11**	C	-1.213271	2.212824	2.752377	**52**	C	0.053626	3.872616	-2.230961
**12**	H	-2.165108	2.731275	2.754656	**53**	H	-0.874602	3.952485	-2.788426
**13**	C	-2.062179	0.095641	-1.497867	**54**	C	-1.981052	-4.394607	-0.272515
**14**	H	-1.573742	-0.419239	-2.318739	**55**	H	-2.663498	-5.234919	-0.360285
**15**	C	-3.165240	1.397734	0.627624	**56**	C	1.076449	-2.184926	-4.628537
**16**	H	-3.612429	1.895790	1.479221	**57**	H	1.103031	-2.610782	-5.626495
**17**	C	-0.861150	-2.706060	1.079617	**58**	C	4.119097	2.237535	0.171777
**18**	C	0.923444	1.777733	3.707973	**59**	H	5.092098	2.081237	0.619856
**19**	H	1.680832	1.918866	4.471904	**60**	C	3.742658	3.476876	-0.257711
**20**	C	-3.409461	0.414544	-1.546539	**61**	H	4.406929	4.329434	-0.151015
**21**	H	-4.004887	0.148374	-2.410955	**62**	C	-2.055572	-3.775818	3.398768
**22**	C	3.225801	1.145704	0.059374	**63**	H	-2.514618	-4.170224	4.299338
**23**	C	-3.970006	1.079858	-0.458902	**64**	C	1.825035	-1.043411	-4.328659
**24**	C	1.019369	-1.060598	-2.036976	**65**	H	2.437177	-0.583918	-5.100334
**25**	C	3.554347	-0.206485	0.486896	**66**	C	2.069010	4.892736	-1.405139
**26**	C	-0.464216	-2.791560	-1.237312	**67**	H	2.728393	5.748389	-1.292008
**27**	C	1.629506	2.515441	-0.993235	**68**	C	4.816306	-0.594208	0.960456
**28**	C	-5.411357	1.464863	-0.398609	**69**	H	5.625333	0.124741	1.048416
**29**	C	-0.546105	-2.192161	2.354126	**70**	C	0.868092	5.010014	-2.062908
**30**	H	0.184790	-1.402296	2.426492	**71**	H	0.556358	5.965897	-2.470870
**31**	C	2.483515	3.649163	-0.877408	**72**	C	-2.350450	-4.320156	2.171916
**32**	C	1.178066	0.925522	2.648427	**73**	H	-3.036957	-5.157170	2.083489
**33**	H	2.118659	0.390000	2.567337	**74**	C	4.012069	-2.851540	1.181814
**34**	C	0.280789	-2.224224	-2.351749	**75**	H	4.192235	-3.890278	1.446190
**35**	C	-1.751021	-3.815046	0.996330	**76**	C	5.043384	-1.916960	1.308784
**36**	C	0.422963	2.653607	-1.709494	**77**	H	6.019196	-2.224030	1.671446
**37**	H	-0.184995	1.777289	-1.870682	**78**	C	-7.460295	1.439508	-1.517630
**38**	C	1.798671	-0.489738	-3.051429	**79**	H	-7.588083	2.521528	-1.446941
**39**	H	2.390688	0.396745	-2.844352	**80**	H	-7.836441	1.069747	-2.469414
**40**	C	2.756804	-2.468087	0.717040	**81**	H	-7.978078	0.958673	-0.685340
**41**	H	1.972427	-3.213709	0.627162	**82**	O	-6.068324	1.103348	-1.495261

Table S27. Cartesian coordinates of the optimized ^{3}MLCT structure of complex **1b**.

**1**	Ir	0.791486	-0.291996	-0.247774	**42**	C	-0.863617	2.761266	5.232385
**2**	N	0.279473	0.589329	1.701781	**43**	H	-1.913903	3.058693	5.210245
**3**	N	1.739878	1.572029	-0.436765	**44**	H	-0.250765	3.662433	5.341123
**4**	O	-6.013242	1.644216	0.673894	**45**	H	-0.695294	2.150635	6.125736
**5**	N	-1.304445	0.163472	-0.375625	**46**	C	-0.763963	-3.059637	3.312991
**6**	N	0.035656	-2.255006	-0.183821	**47**	H	-0.606280	-2.641661	4.302269
**7**	C	2.614856	-0.805954	0.342502	**48**	C	-0.688948	-4.076132	-1.581830
**8**	C	-0.477383	2.007573	3.998969	**49**	H	-0.754049	-4.498427	-2.576102
**9**	C	-1.878110	0.779441	0.727018	**50**	C	0.638578	-2.535728	-3.804156
**10**	C	-0.997657	1.053298	1.825291	**51**	H	0.277338	-3.519484	-4.084499
**11**	C	-1.377614	1.762046	2.983662	**52**	C	-0.671349	3.926678	-1.902235
**12**	H	-2.393279	2.130273	3.072232	**53**	H	-1.651742	3.906386	-2.367632
**13**	C	-2.113467	-0.200441	-1.406120	**54**	C	-1.240006	-4.723133	-0.512696
**14**	H	-1.624577	-0.691236	-2.240441	**55**	H	-1.755498	-5.670143	-0.640308
**15**	C	-3.239975	1.074666	0.734350	**56**	C	1.220477	-1.725187	-4.775352
**16**	H	-3.696620	1.562997	1.587753	**57**	H	1.296146	-2.083895	-5.796744
**17**	C	-0.450391	-2.913492	0.919508	**58**	C	3.743785	2.769499	0.140181
**18**	C	0.839397	1.522785	3.834724	**59**	H	4.750912	2.735795	0.534907
**19**	H	1.594184	1.679885	4.597492	**60**	C	3.176647	3.958676	-0.222193
**20**	C	-3.454331	0.058219	-1.442482	**61**	H	3.724618	4.890276	-0.118305
**21**	H	-4.043249	-0.241704	-2.299604	**62**	C	-1.467460	-4.272650	3.170883
**22**	C	3.003111	1.574538	0.023893	**63**	H	-1.859556	-4.778178	4.047098
**23**	C	-4.054502	0.734429	-0.334308	**64**	C	1.706151	-0.455734	-4.443586
**24**	C	1.036214	-0.791009	-2.136746	**65**	H	2.158385	0.165507	-5.210523
**25**	C	3.535304	0.271631	0.411965	**66**	C	1.257146	5.174931	-1.187465
**26**	C	-0.049396	-2.832273	-1.395572	**67**	H	1.805688	6.105621	-1.077499
**27**	C	1.166707	2.741622	-0.873183	**68**	C	4.855952	0.051959	0.805825
**28**	C	-5.475501	1.069271	-0.260537	**69**	H	5.575778	0.863082	0.838938
**29**	C	-0.265279	-2.394362	2.217330	**70**	C	0.002897	5.154885	-1.746042
**30**	H	0.281087	-1.472985	2.339277	**71**	H	-0.463957	6.075513	-2.080057
**31**	C	1.870102	3.977278	-0.754397	**72**	C	-1.636569	-4.816053	1.921157
**32**	C	1.162760	0.836758	2.688399	**73**	H	-2.156098	-5.760238	1.788071
**33**	H	2.161569	0.440438	2.543004	**74**	C	4.362533	-2.299569	1.109271
**34**	C	0.538830	-2.073482	-2.492352	**75**	H	4.693119	-3.296682	1.383828
**35**	C	-1.124357	-4.163200	0.777304	**76**	C	5.264316	-1.232161	1.154864
**36**	C	-0.106585	2.745775	-1.480016	**77**	H	6.290151	-1.402241	1.465361
**37**	H	-0.629290	1.810507	-1.609393	**78**	C	-7.543080	0.965256	-1.350055
**38**	C	1.615113	0.009285	-3.139235	**79**	H	-7.710118	2.043358	-1.284478
**39**	H	1.998386	0.993702	-2.893455	**80**	H	-7.934029	0.578794	-2.290644
**40**	C	3.048180	-2.089502	0.710797	**81**	H	-8.038536	0.479954	-0.505381
**41**	H	2.357111	-2.925286	0.679113	**82**	O	-6.149645	0.669638	-1.356153

Table S28. Cartesian coordinates of the optimized ^{3}MC structure of complex **1b**.

**1**	Ir	0.940818	0.137468	-0.431896	**42**	C	-0.794544	1.700507	5.646122
**2**	N	0.271872	0.424181	1.675557	**43**	H	-1.855193	1.540887	5.849212
**3**	N	0.677839	2.194231	-0.595628	**44**	H	-0.556853	2.755333	5.805771
**4**	O	-5.671334	0.384816	0.450976	**45**	H	-0.220171	1.122293	6.378517
**5**	N	-1.315027	-2.040895	1.018368	**46**	C	3.207029	-2.506900	2.691863
**6**	N	1.267454	-1.899929	-0.368127	**47**	H	3.692106	-1.936312	3.477749
**7**	C	2.863568	0.708120	-0.690188	**48**	C	0.774548	-4.003688	-1.418911
**8**	C	-0.430812	1.272335	4.260882	**49**	H	0.309938	-4.532277	-2.241988
**9**	C	-1.790469	-0.878783	1.475038	**50**	C	-0.557753	-2.301126	-3.530781
**10**	C	-0.872259	-0.000412	2.245197	**51**	H	-0.485944	-3.359302	-3.762504
**11**	C	-1.240907	0.411449	3.523035	**52**	C	-2.927956	2.756289	-0.179359
**12**	H	-2.172134	0.041742	3.940158	**53**	H	-3.842235	2.174318	-0.098104
**13**	C	-2.138991	-2.812369	0.308562	**54**	C	1.399366	-4.689442	-0.415736
**14**	H	-1.727387	-3.756243	-0.038459	**55**	H	1.442580	-5.774431	-0.426527
**15**	C	-3.099724	-0.454713	1.257288	**56**	C	-1.194809	-1.452207	-4.427527
**16**	H	-3.457720	0.492322	1.644632	**57**	H	-1.623776	-1.854869	-5.339593
**17**	C	1.931819	-2.558171	0.636588	**58**	C	1.794040	4.314566	-0.774617
**18**	C	0.749882	1.701382	3.648796	**59**	H	2.723559	4.855159	-0.900176
**19**	H	1.430029	2.378313	4.154797	**60**	C	0.616609	4.986520	-0.600052
**20**	C	-3.450246	-2.462146	-0.002619	**61**	H	0.593217	6.072159	-0.587709
**21**	H	-4.071234	-3.112390	-0.607048	**62**	C	3.249195	-3.915423	2.723369
**22**	C	1.808921	2.902202	-0.762100	**63**	H	3.755914	-4.424051	3.536955
**23**	C	-3.935993	-1.250349	0.480536	**64**	C	-1.270453	-0.081312	-4.162661
**24**	C	-0.096032	-0.398598	-2.061578	**65**	H	-1.760897	0.579584	-4.871807
**25**	C	3.021495	2.103031	-0.895795	**66**	C	-1.836365	4.903028	-0.265212
**26**	C	0.702067	-2.593891	-1.370378	**67**	H	-1.864619	5.988550	-0.247618
**27**	C	-0.525431	2.842019	-0.453180	**68**	C	4.272646	2.637201	-1.220580
**28**	C	-5.302166	-0.738529	0.174607	**69**	H	4.399571	3.702479	-1.387155
**29**	C	2.568408	-1.840664	1.671320	**70**	C	-2.987549	4.164609	-0.132669
**30**	H	2.564305	-0.759510	1.639802	**71**	H	-3.944339	4.660264	-0.004972
**31**	C	-0.586840	4.264008	-0.435536	**72**	C	2.660860	-4.636399	1.712428
**32**	C	1.056784	1.263780	2.375263	**73**	H	2.695561	-5.721940	1.709097
**33**	H	1.964180	1.593671	1.878072	**74**	C	5.224177	0.416882	-1.169663
**34**	C	-0.005534	-1.786708	-2.353483	**75**	H	6.084741	-0.236693	-1.281213
**35**	C	1.998758	-3.980012	0.650312	**76**	C	5.369947	1.794404	-1.352947
**36**	C	-1.724152	2.107817	-0.336832	**77**	H	6.339731	2.209473	-1.608463
**37**	H	-1.679560	1.027172	-0.401343	**78**	O	-6.054754	-1.644935	-0.439992
**38**	C	-0.723006	0.441618	-2.997102	**79**	C	-7.374629	-1.214923	-0.790064
**39**	H	-0.789330	1.508846	-2.811078	**80**	H	-7.326699	-0.355022	-1.461310
**40**	C	3.982960	-0.122400	-0.846572	**81**	H	-7.838295	-2.064722	-1.287203
**41**	H	3.886830	-1.195304	-0.711163	**82**	H	-7.933904	-0.942305	0.107188

Table S29. Cartesian coordinates of the optimized ^{3}MC/S_0 MECP structure of complex **1b**.

**1**	Ir	0.953870	0.139708	-0.437926	**42**	C	-0.789324	1.696058	5.633903
**2**	N	0.260743	0.424565	1.657315	**43**	H	-1.849533	1.537535	5.838995
**3**	N	0.691850	2.198442	-0.597045	**44**	H	-0.550516	2.750986	5.791658
**4**	O	-5.676870	0.381062	0.446450	**45**	H	-0.213800	1.117771	6.364902
**5**	N	-1.322570	-2.046542	1.012361	**46**	C	3.208381	-2.507133	2.691649
**6**	N	1.266968	-1.901095	-0.366129	**47**	H	3.691535	-1.936036	3.478303
**7**	C	2.886514	0.718589	-0.663649	**48**	C	0.760770	-4.005432	-1.409409
**8**	C	-0.429507	1.266053	4.248203	**49**	H	0.288330	-4.534243	-2.227911
**9**	C	-1.796985	-0.882687	1.465168	**50**	C	-0.565947	-2.300503	-3.519593
**10**	C	-0.878516	-0.003829	2.232353	**51**	H	-0.475455	-3.355409	-3.758918
**11**	C	-1.241834	0.405561	3.512862	**52**	C	-2.915557	2.754345	-0.178860
**12**	H	-2.170819	0.035712	3.934187	**53**	H	-3.828662	2.170553	-0.098151
**13**	C	-2.147240	-2.818675	0.304435	**54**	C	1.399879	-4.690732	-0.414665
**14**	H	-1.736082	-3.763323	-0.040764	**55**	H	1.450993	-5.775210	-0.430463
**15**	C	-3.106271	-0.458756	1.248821	**56**	C	-1.191654	-1.451239	-4.423770
**16**	H	-3.464848	0.488357	1.635781	**57**	H	-1.592424	-1.849682	-5.349983
**17**	C	1.934533	-2.559336	0.636086	**58**	C	1.804001	4.320861	-0.770052
**18**	C	0.749134	1.695442	3.632067	**59**	H	2.732773	4.864156	-0.891140
**19**	H	1.432103	2.370712	4.136561	**60**	C	0.624236	4.990288	-0.601613
**20**	C	-3.457912	-2.467662	-0.008159	**61**	H	0.597504	6.075976	-0.592561
**21**	H	-4.078955	-3.118414	-0.611755	**62**	C	3.250239	-3.915795	2.723457
**22**	C	1.822162	2.908513	-0.756961	**63**	H	3.754179	-4.424022	3.538995
**23**	C	-3.942913	-1.255039	0.473524	**64**	C	-1.286752	-0.084696	-4.148044
**24**	C	-0.157727	-0.407311	-2.021131	**65**	H	-1.759746	0.579779	-4.865334
**25**	C	3.038149	2.111547	-0.884487	**66**	C	-1.828256	4.902400	-0.267081
**26**	C	0.684579	-2.595826	-1.357074	**67**	H	-1.857786	5.987901	-0.249262
**27**	C	-0.513052	2.843714	-0.453534	**68**	C	4.285040	2.647951	-1.221710
**28**	C	-5.308589	-0.742461	0.169118	**69**	H	4.407923	3.712976	-1.393747
**29**	C	2.571142	-1.841101	1.670042	**70**	C	-2.978178	4.162715	-0.134032
**30**	H	2.565989	-0.760019	1.637302	**71**	H	-3.935929	4.656426	-0.006625
**31**	C	-0.577538	4.265448	-0.437470	**72**	C	2.663504	-4.637189	1.712287
**32**	C	1.049068	1.262821	2.355779	**73**	H	2.696476	-5.722915	1.710025
**33**	H	1.952292	1.595583	1.853727	**74**	C	5.243214	0.430575	-1.166593
**34**	C	-0.040264	-1.789785	-2.328883	**75**	H	6.103875	-0.222055	-1.284699
**35**	C	2.001300	-3.981083	0.649636	**76**	C	5.383386	1.806905	-1.360016
**36**	C	-1.710729	2.107701	-0.333992	**77**	H	6.349114	2.222909	-1.628939
**37**	H	-1.664002	1.027414	-0.394946	**78**	O	-6.062447	-1.648112	-0.444764
**38**	C	-0.777148	0.432030	-2.961957	**79**	C	-7.381726	-1.215525	-0.793282
**39**	H	-0.860798	1.496701	-2.768813	**80**	H	-7.332241	-0.355067	-1.463699
**40**	C	4.006943	-0.108943	-0.823631	**81**	H	-7.847385	-2.064025	-1.290624
**41**	H	3.916341	-1.180847	-0.676139	**82**	H	-7.939247	-0.942256	0.104875

Figure S1. Electronic absorption spectra of complexes **1a** – **4a** in CH_2Cl_2 (black) and CH_3CN (red) at 298 K.

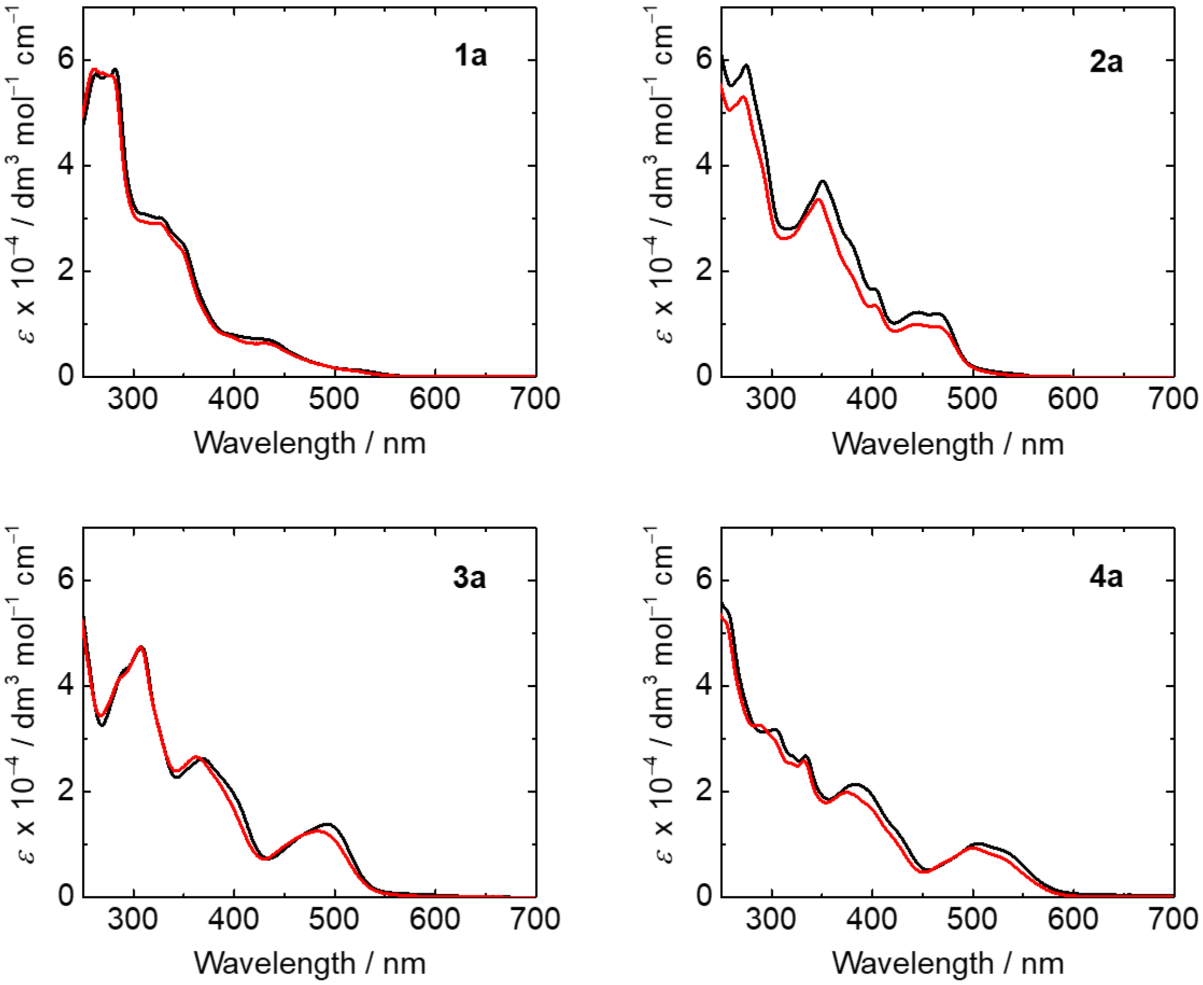

Figure S2. Electronic absorption spectra of complexes **1b** – **4b** in CH_2Cl_2 (black) and CH_3CN (red) at 298 K.

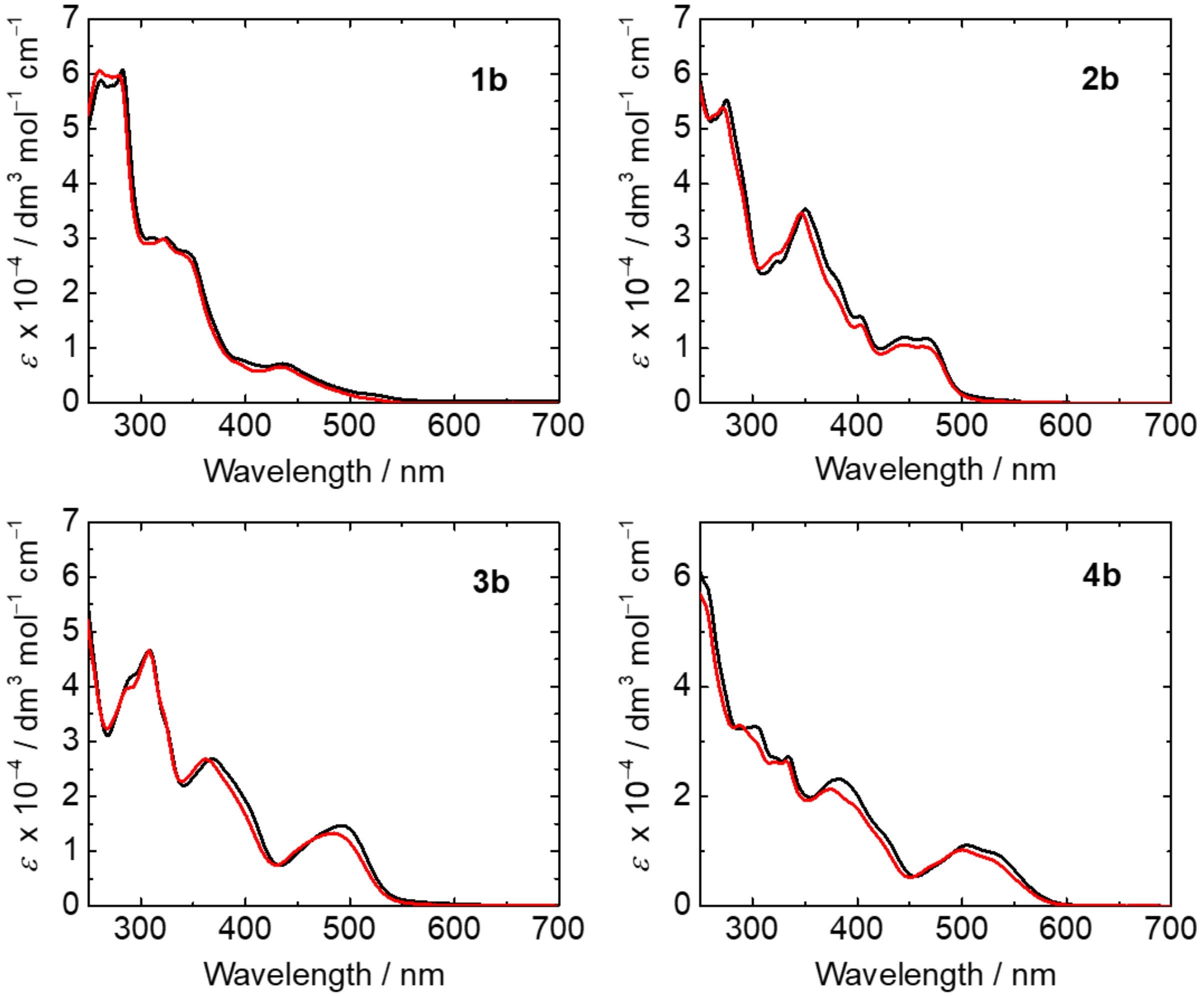

Figure S3. Normalized emission spectra of complexes **1a** – **4a** in degassed CH_2Cl_2 (black) and CH_3CN (red) at 298 K and alcohol glass at 77 K (blue).

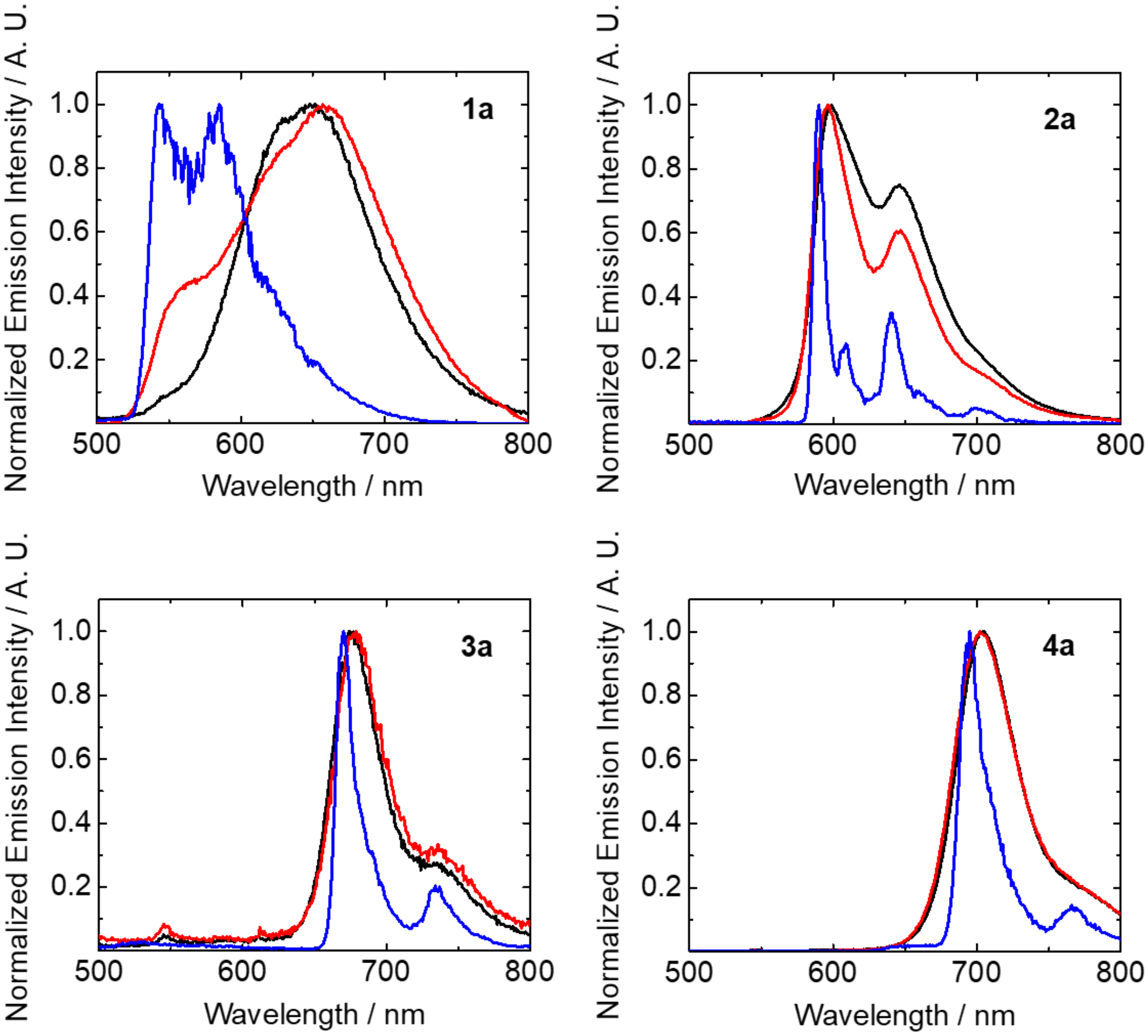

Figure S4. Normalized emission spectra of complexes **1b** – **4b** in degassed CH_2Cl_2 (black) and CH_3CN (red) at 298 K and alcohol glass at 77 K (blue).

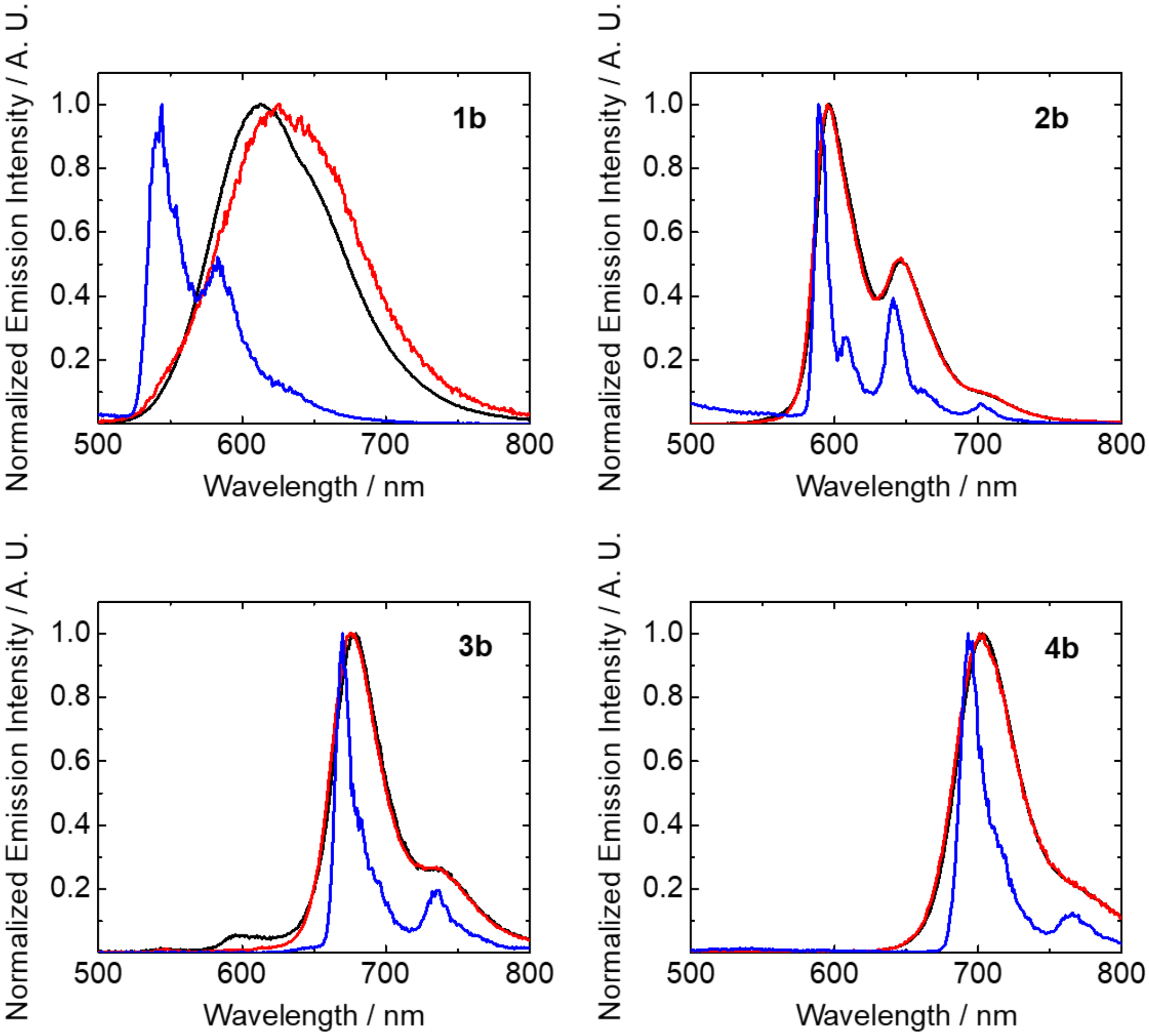

Figure S5. Latimer diagrams showing the excited-state redox potentials of complexes **1a** – **4a** versus SCE.

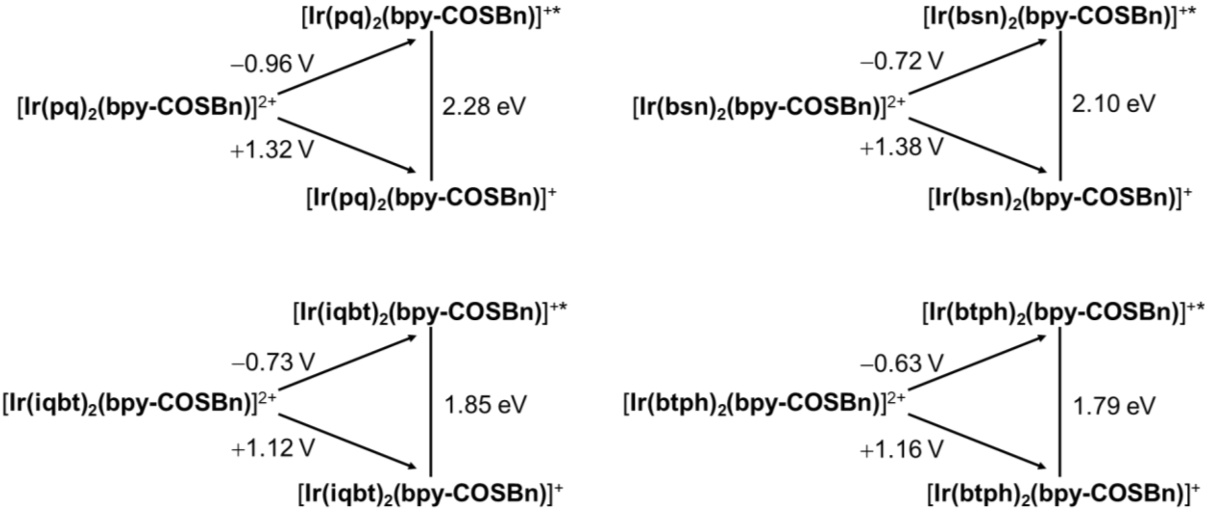

Figure S6. HPLC chromatograms of the reaction mixtures of complexes **1a** – **4a** (20 μM) without (black) or with L-Cys (25 μM) (red) in aerated potassium phosphate buffer (50 mM, pH 7.0)/DMSO (3:2, *v*/*v*) containing TCEP (250 μM) after incubation at 298 K for 1 h. The absorbance was monitored at 350 nm.

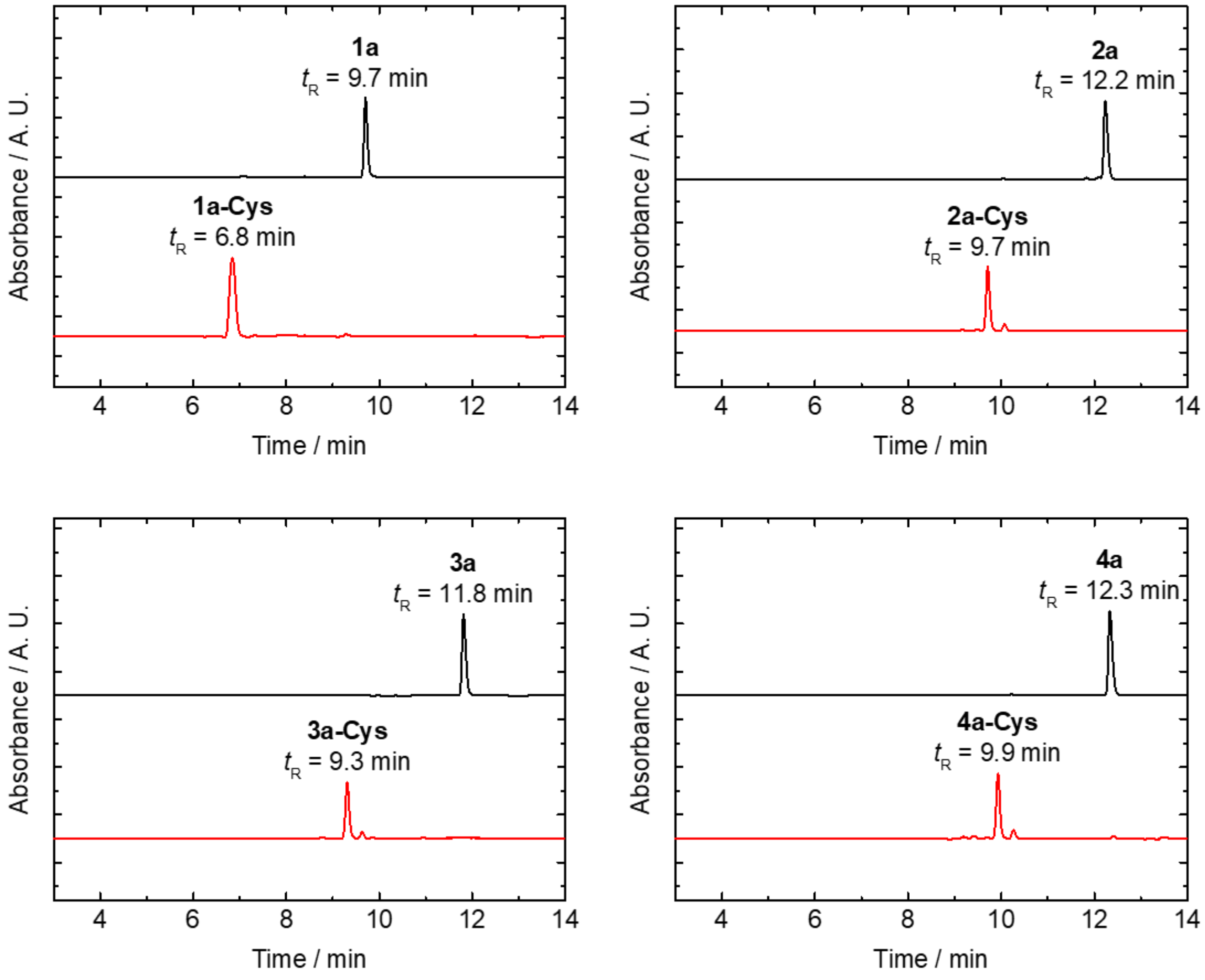

Figure S7. ESI mass spectra of the reaction mixtures of complexes **1a** – **4a** (20 μM) with L-Cys (25 μM) in aerated potassium phosphate buffer (50 mM, pH 7.0)/DMSO (3:2, *v*/*v*) containing TCEP (250 μM) after incubation at 298 K for 1 h.

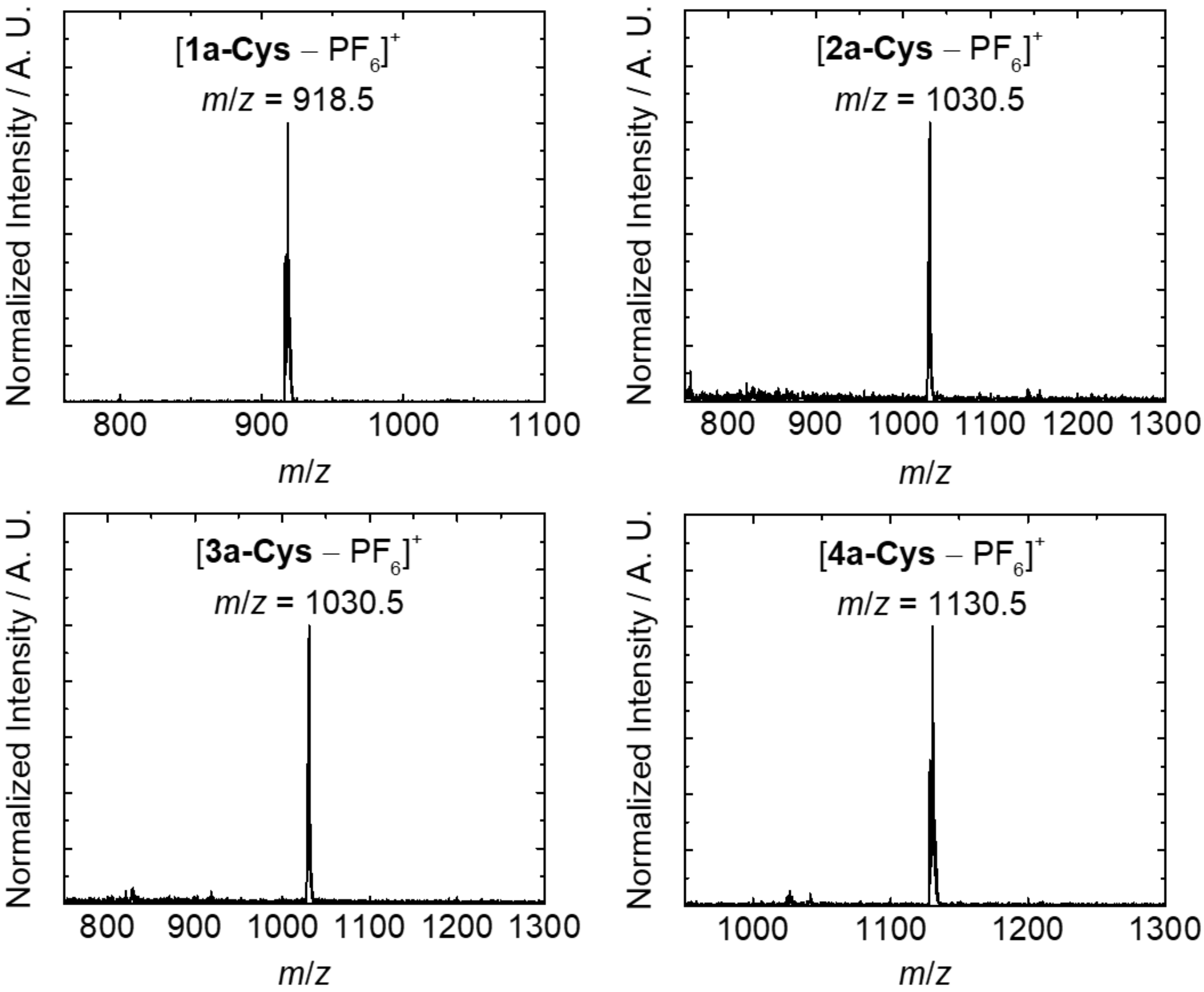

Figure S8. ^{1}H NMR spectrum of conjugate **1a-Cys** in CD_3CN at 298 K.

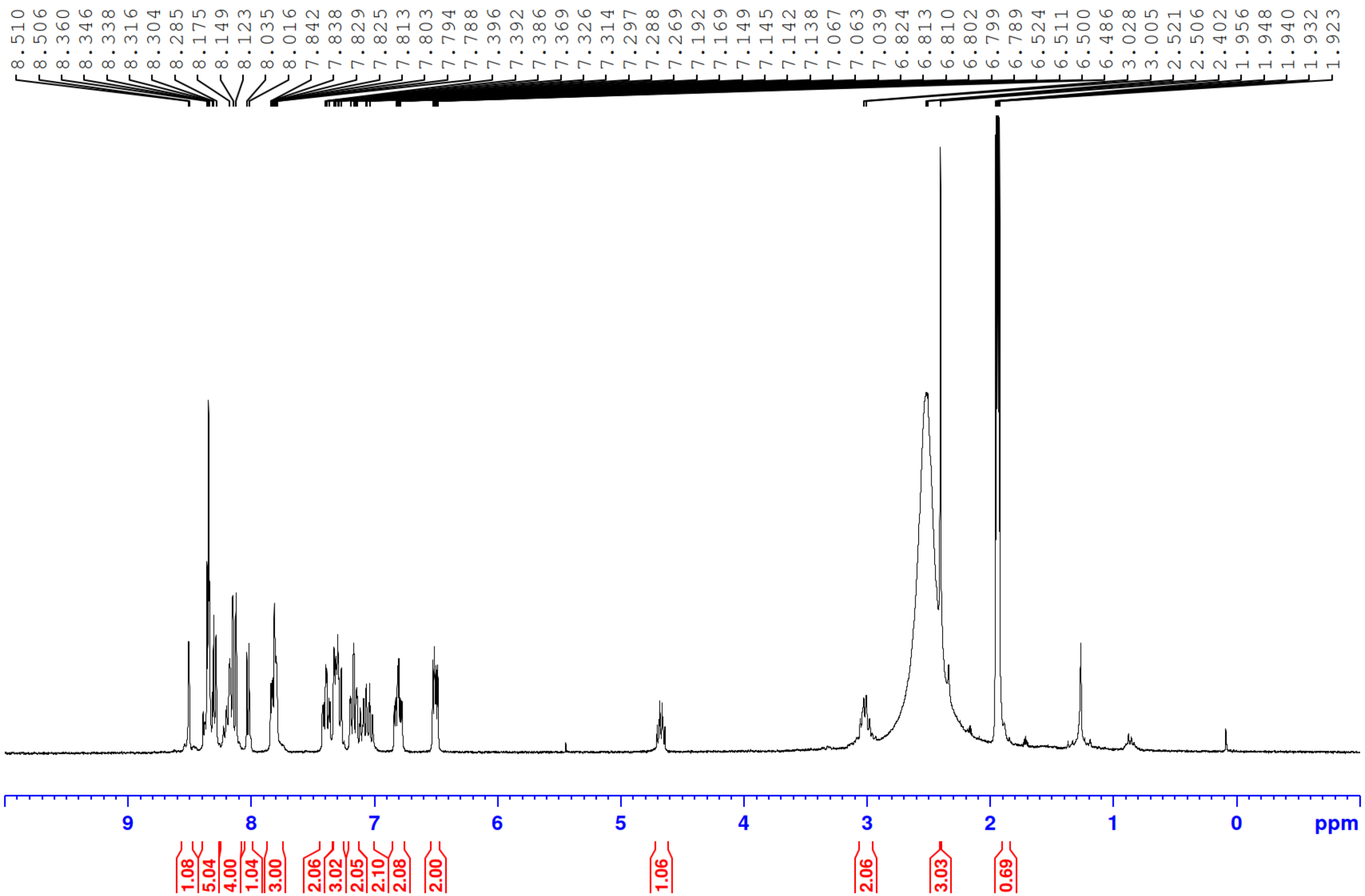

Figure S9. ^1H–^1H COSY NMR spectrum of conjugate **1a-Cys** in CD_3CN at 298 K.

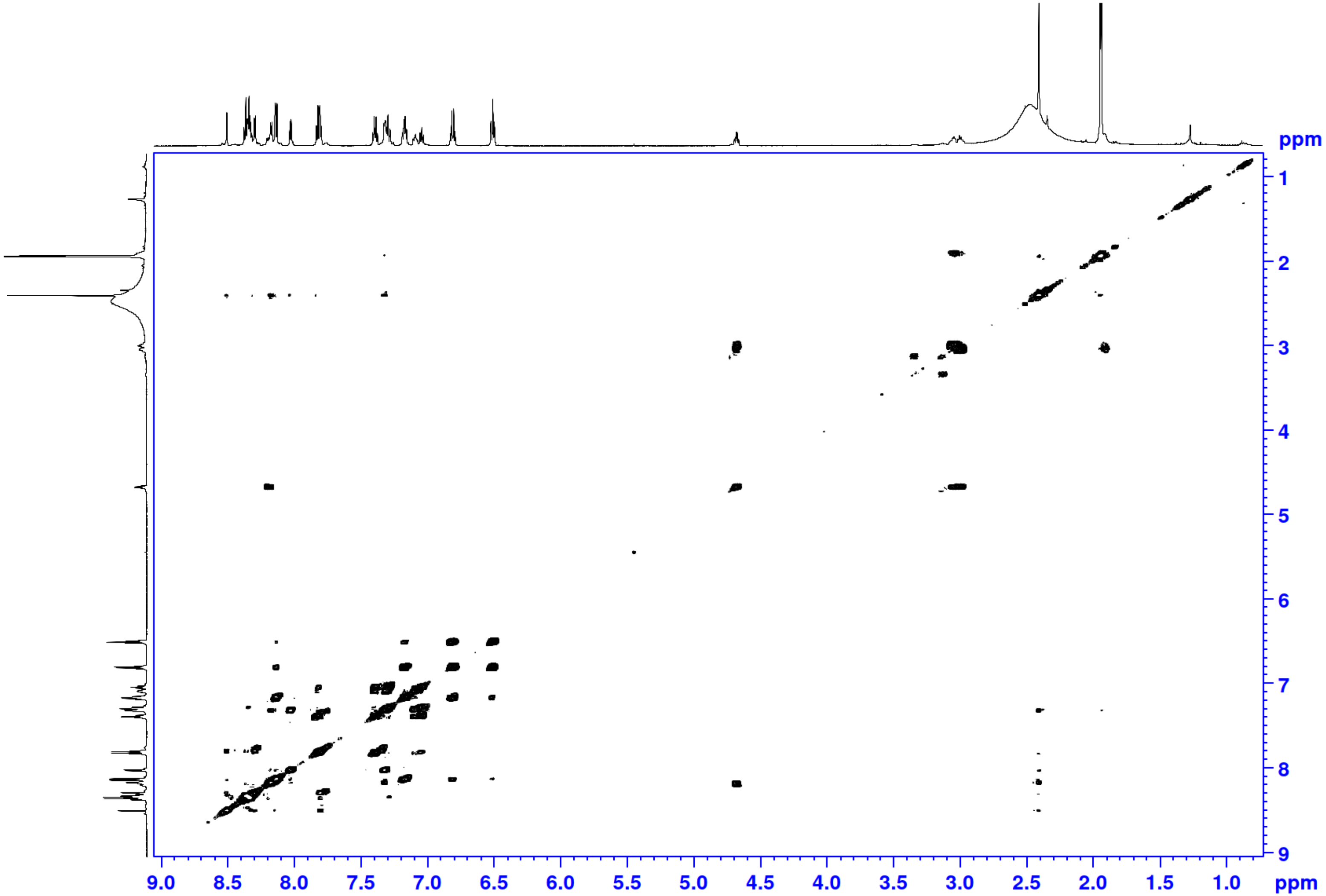

Figure S10. Second-order kinetics for the reaction of the thioester complexes **1a** – **4a** (20 μM) and ligand bpy-COSBn (100 μM) with L-Cys (25 μM for the complexes and 250 μM for the ligand) at different time points in aerated potassium phosphate buffer (50 mM, pH 7.0)/DMSO (3:2, *v*/*v*) containing TCEP (250 μM) after incubation at 298 K. The slope of the linear fit corresponds to the k_2 of the reaction.

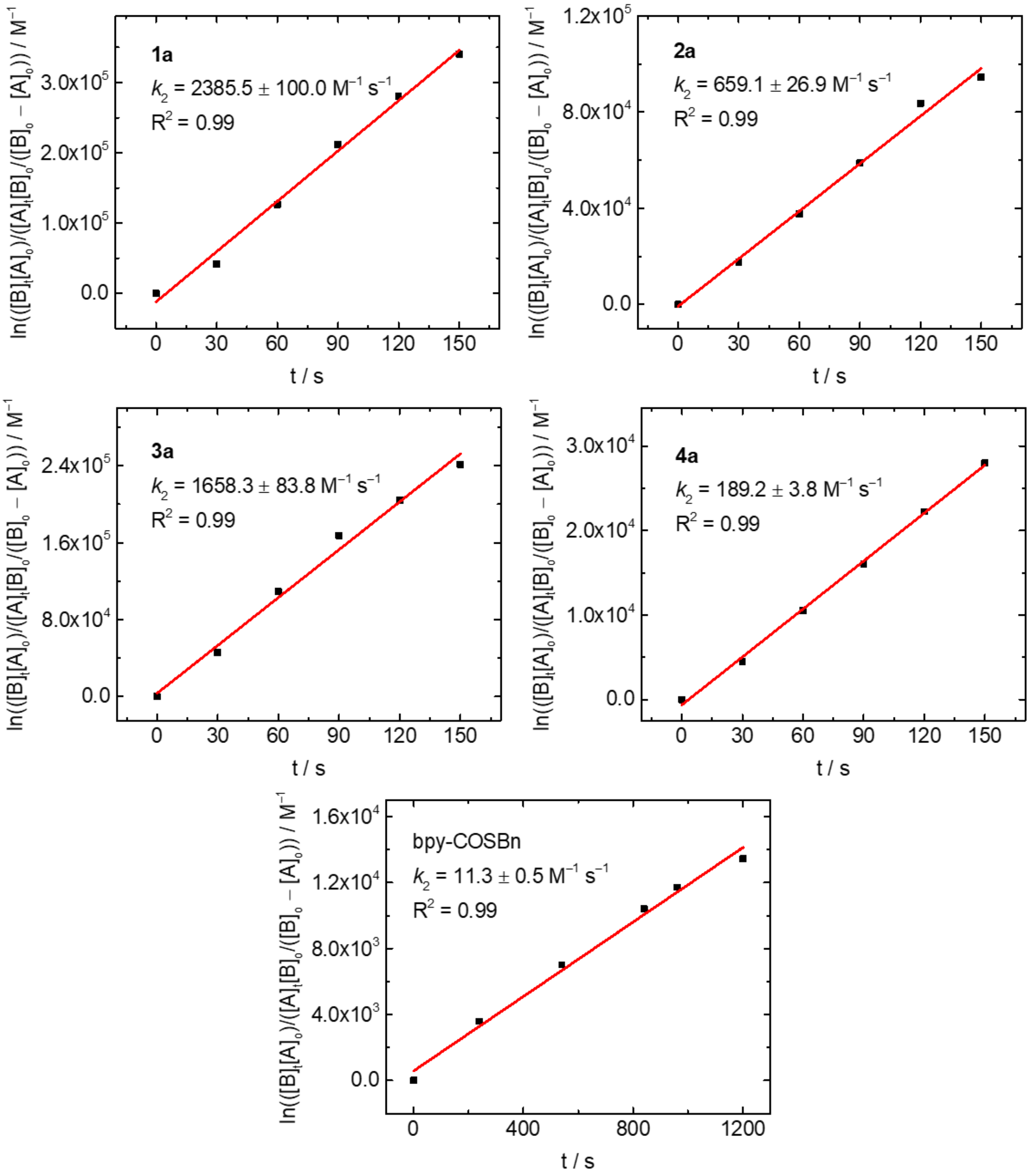

Figure S11. HPLC chromatograms of complex **1a** (20 μM) (black) and the reaction mixtures of complex **1a** (20 μM) upon incubation with L-Lys (2 mM), L-His (2 mM), L-Ser (2 mM), or L-Thr (2 mM) (red) in aerated potassium phosphate buffer (50 mM, pH 7.0)/DMSO (3:2, *v*/*v*) containing TCEP (10 mM) at 298 K for 1 h. The absorbance was monitored at 350 nm.

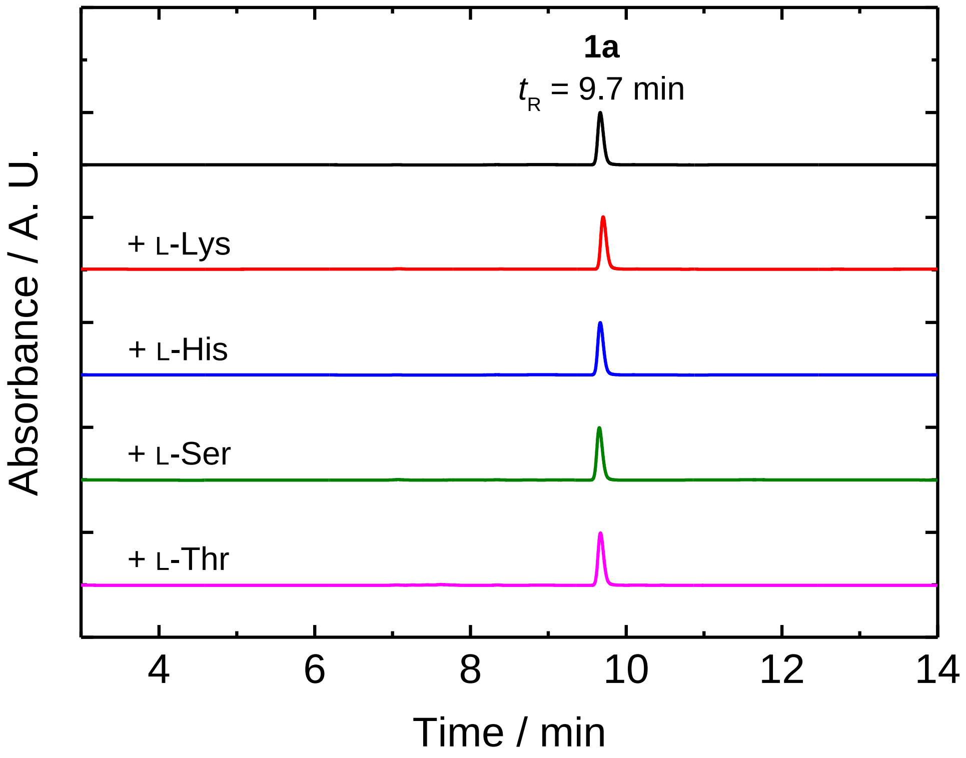

Figure S12. Emission spectra of complex **1a** (10 μM) upon the gradual addition of L-Cys in aerated potassium phosphate buffer (50 mM, pH 7.0)/CH_3CN (3:2, *v*/*v*) containing TCEP (1 mM) at 298 K.

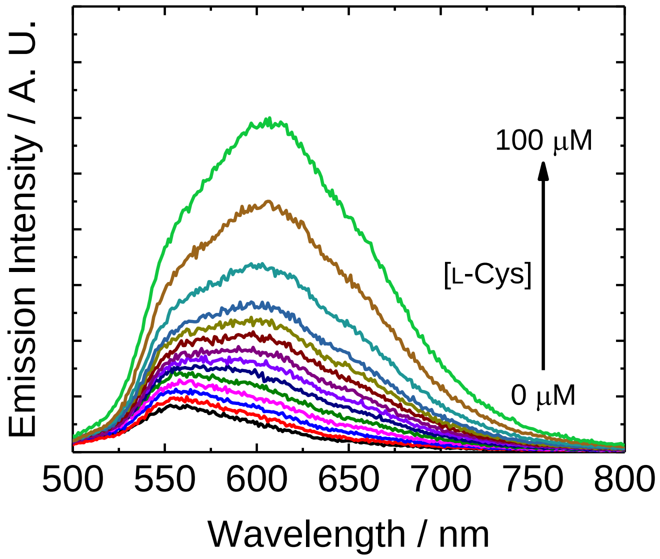

Figure S13. Normalized emission spectra of conjugates **1a-Cys** – **4a-Cys** in degassed CH_3CN (black) and potassium phosphate buffer (50 mM, pH 7.4)/MeOH (2:3, *v*/*v*) (red) at 298 K.

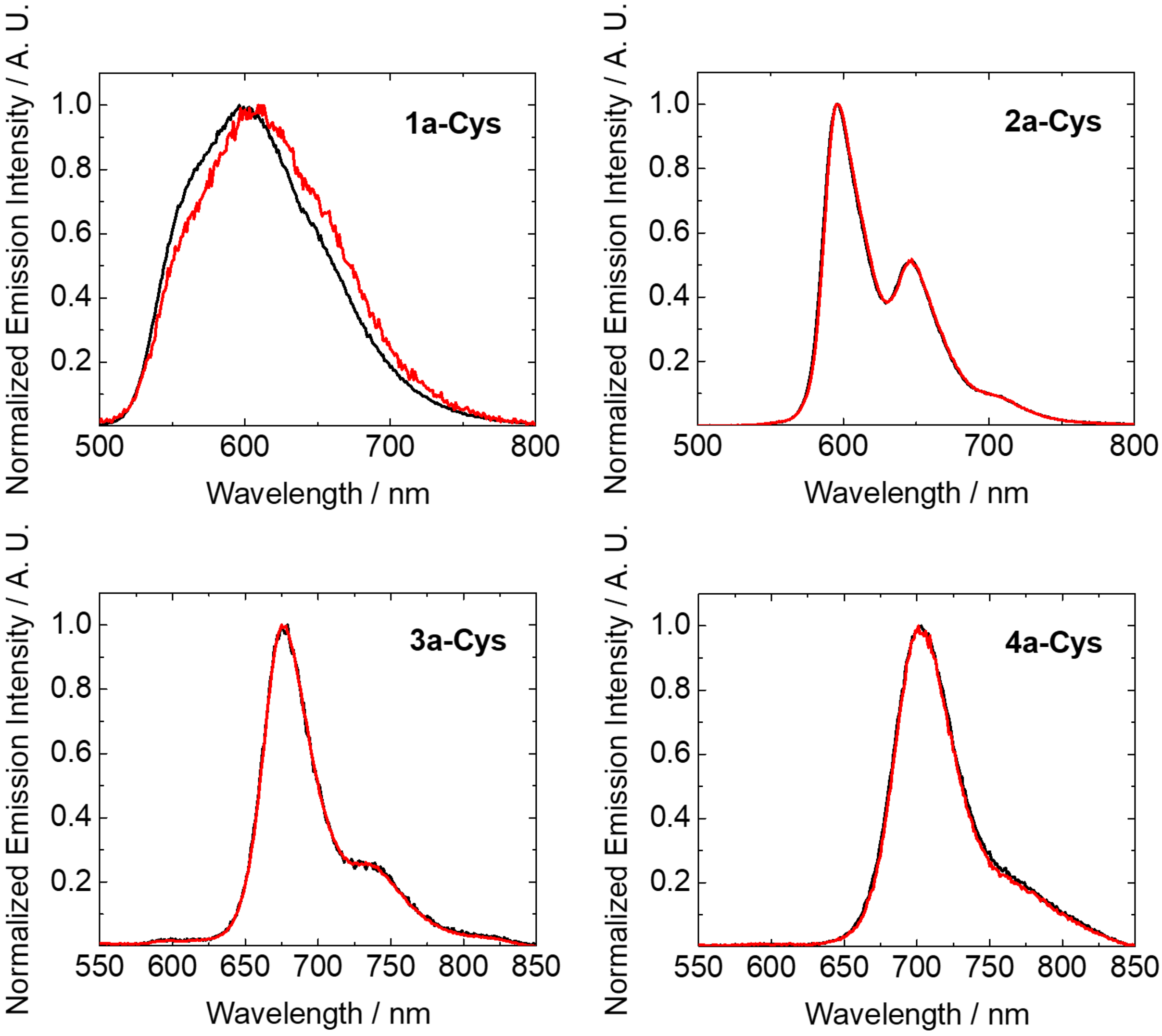

Figure S14. Emission spectra and ESI mass spectra of the reaction mixtures of complex **1a** (10 μM) with (a) L-Cys, (b) ethanethiol, and (c) GSH (100 μM) in aerated potassium phosphate buffer (50 mM, pH 7.0)/CH_3CN (3:2, *v*/*v*) containing TCEP (1 mM) at 298 K for 1 h.

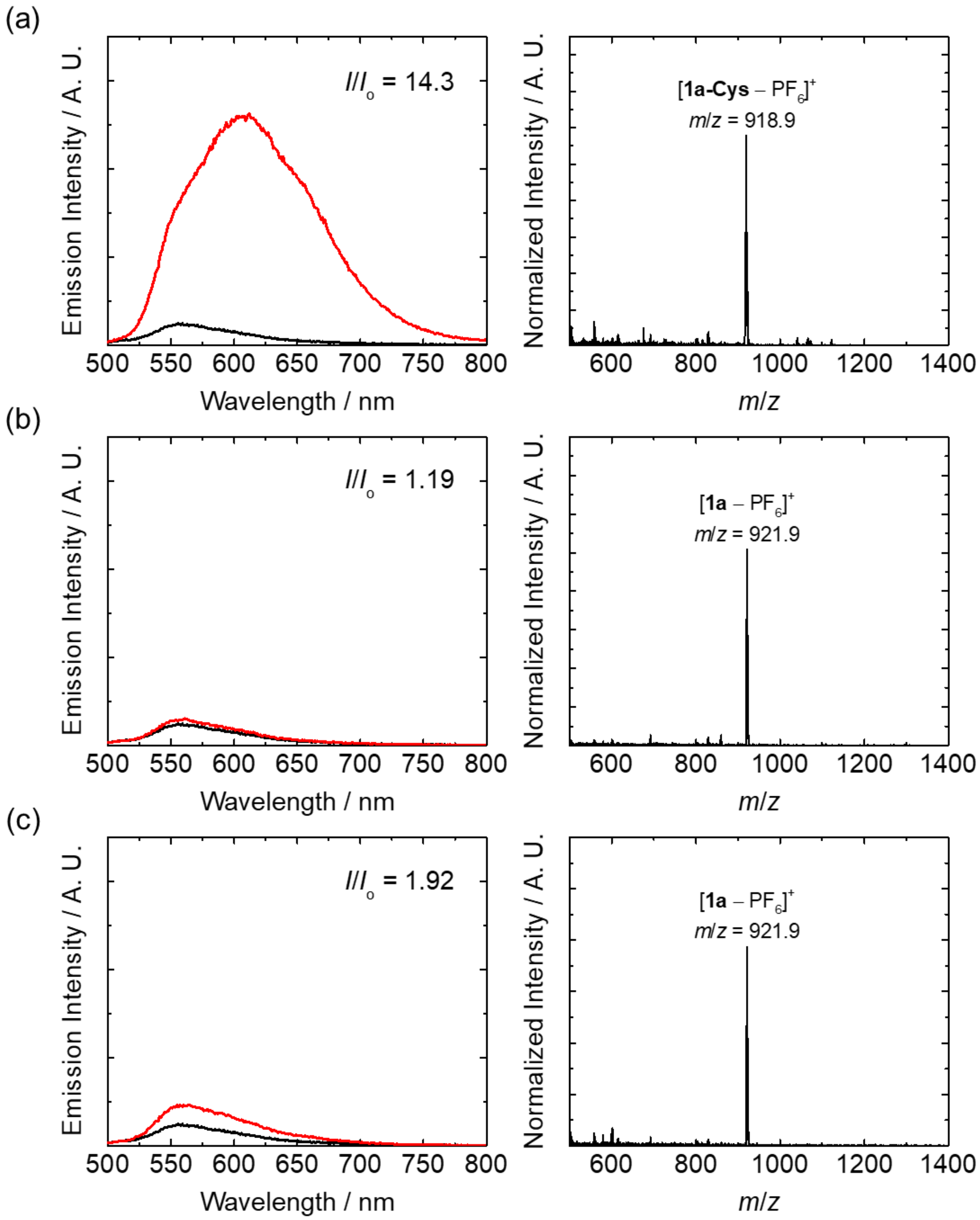

Figure S15. ESI mass spectrum of a CH_2Cl_2 extract of complex **1a** (10 μM) in aerated potassium phosphate buffer (50 mM, pH 7.0)/CH_3CN (3:2, *v*/*v*) containing TCEP (1 mM) after incubation at 37°C for 1 h.

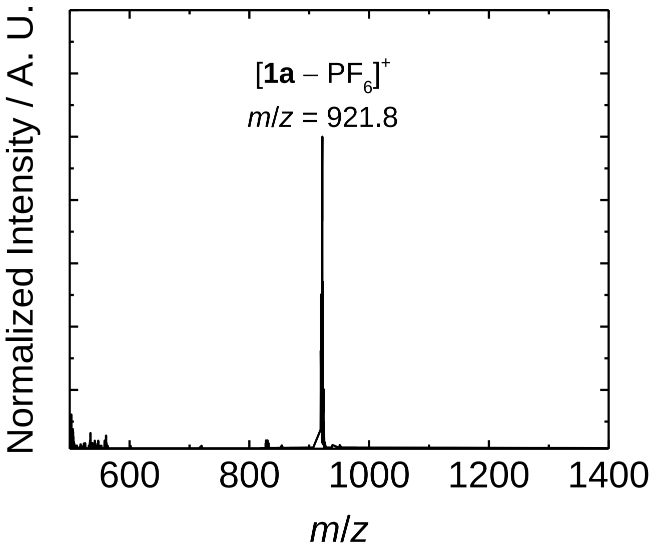

Figure S16. Normalized intracellular emission spectra of HeLa cells incubated with (a) complex **1a** (10 μM, 1 h, λ_{ex} = 405 nm) and (b) conjugate **1a-Cys** (20 μM, 6 h, λ_{ex} = 405 nm).

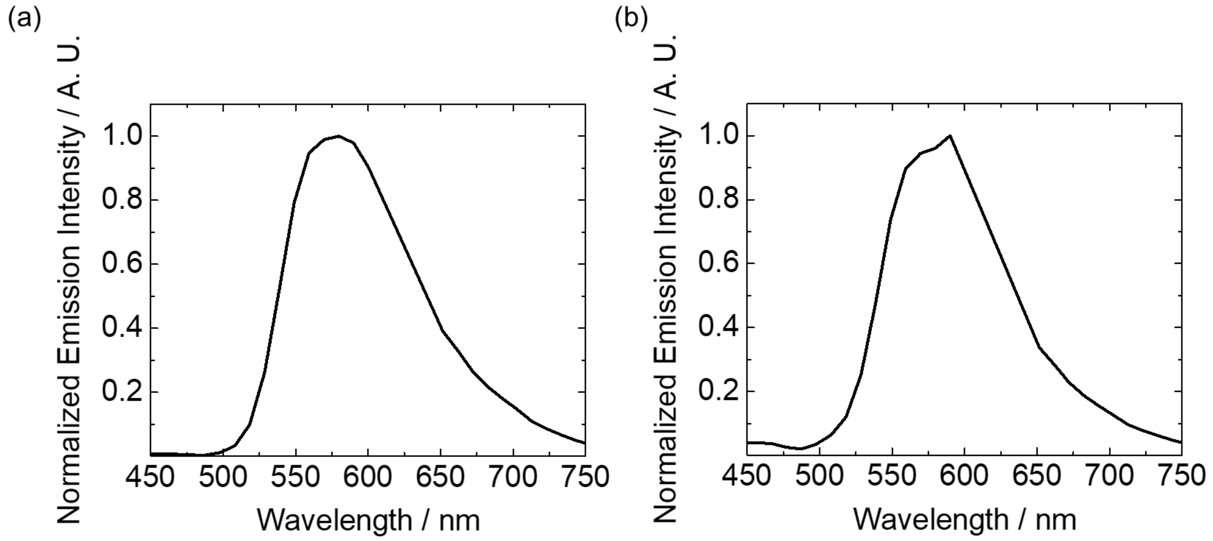

Figure S17. Semipreparative HPLC chromatograms of the crude reaction mixtures of complex **3a** (1 mM) and peptides CASP, CYNT, and CMYI (1.5 mM) in aerated potassium phosphate buffer (50 mM, pH 7.0)/DMSO (3:2, *v*/*v*) containing TCEP (10 mM) at 298 K for 12 h. The absorbance was monitored at 350 nm.

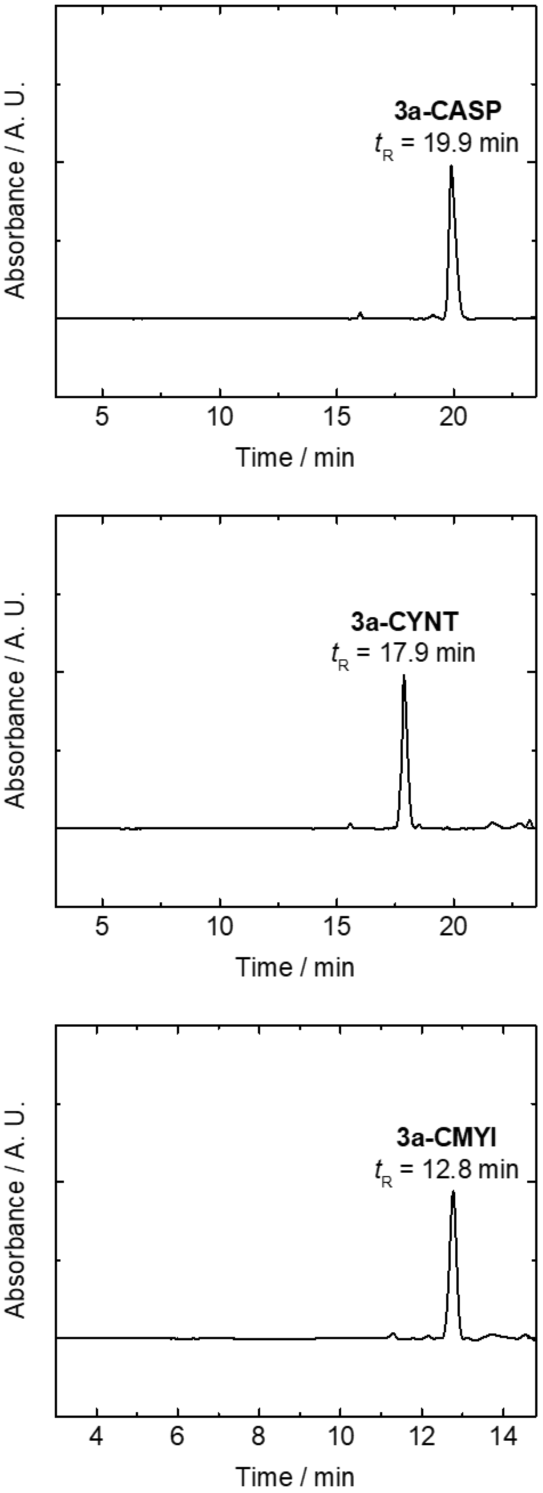

Figure S18. Analytical HPLC chromatograms of the purified conjugates **3a-Cys**, **3a-CASP**, **3a-CYNT**, and **3a-CMYI**. The absorbance was monitored at 350 nm.

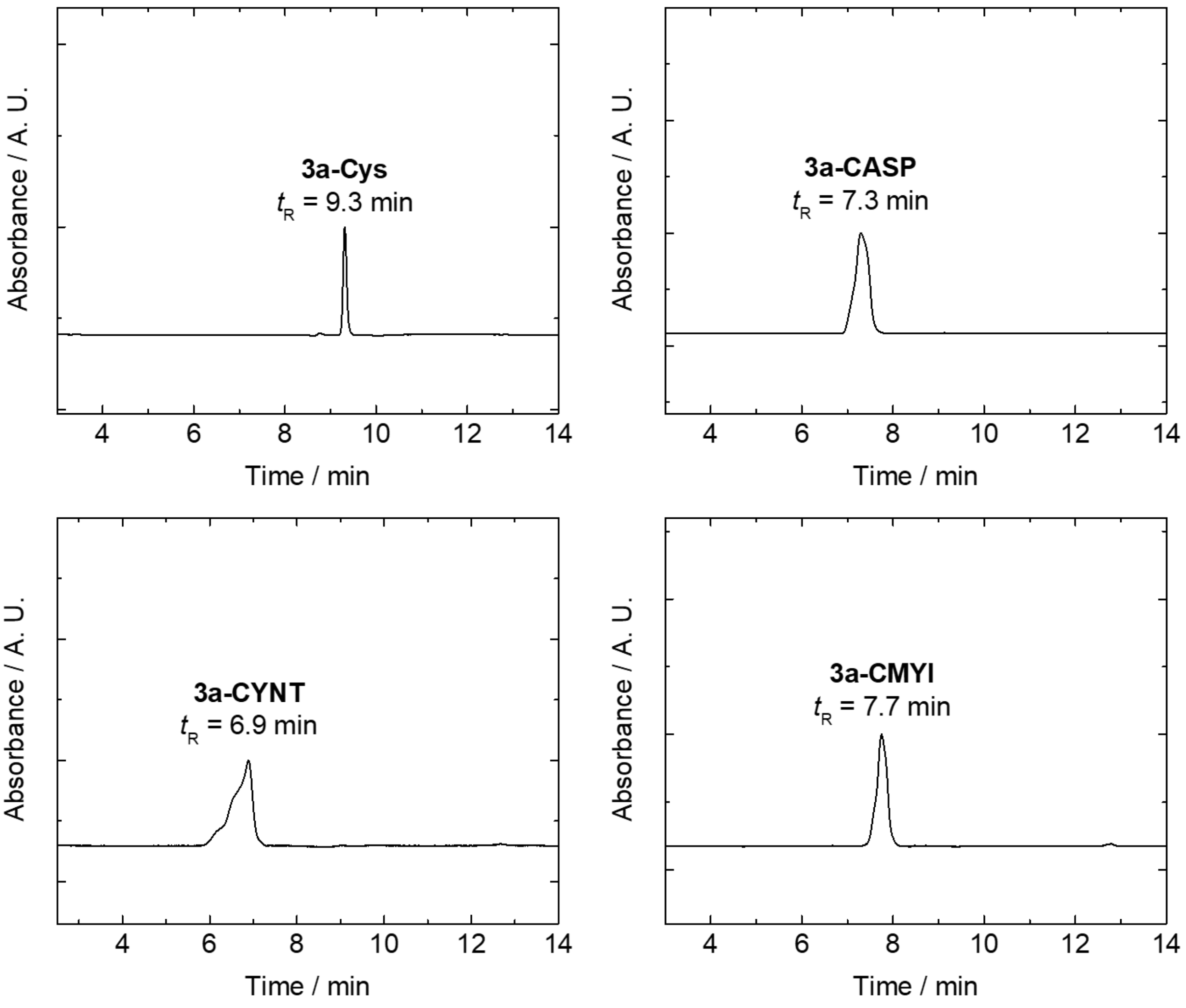

Figure S19. ESI mass spectra of the purified conjugates **3a-Cys**, **3a-CASP**, **3a-CYNT**, and **3a-CMYI** in CH_3CN.

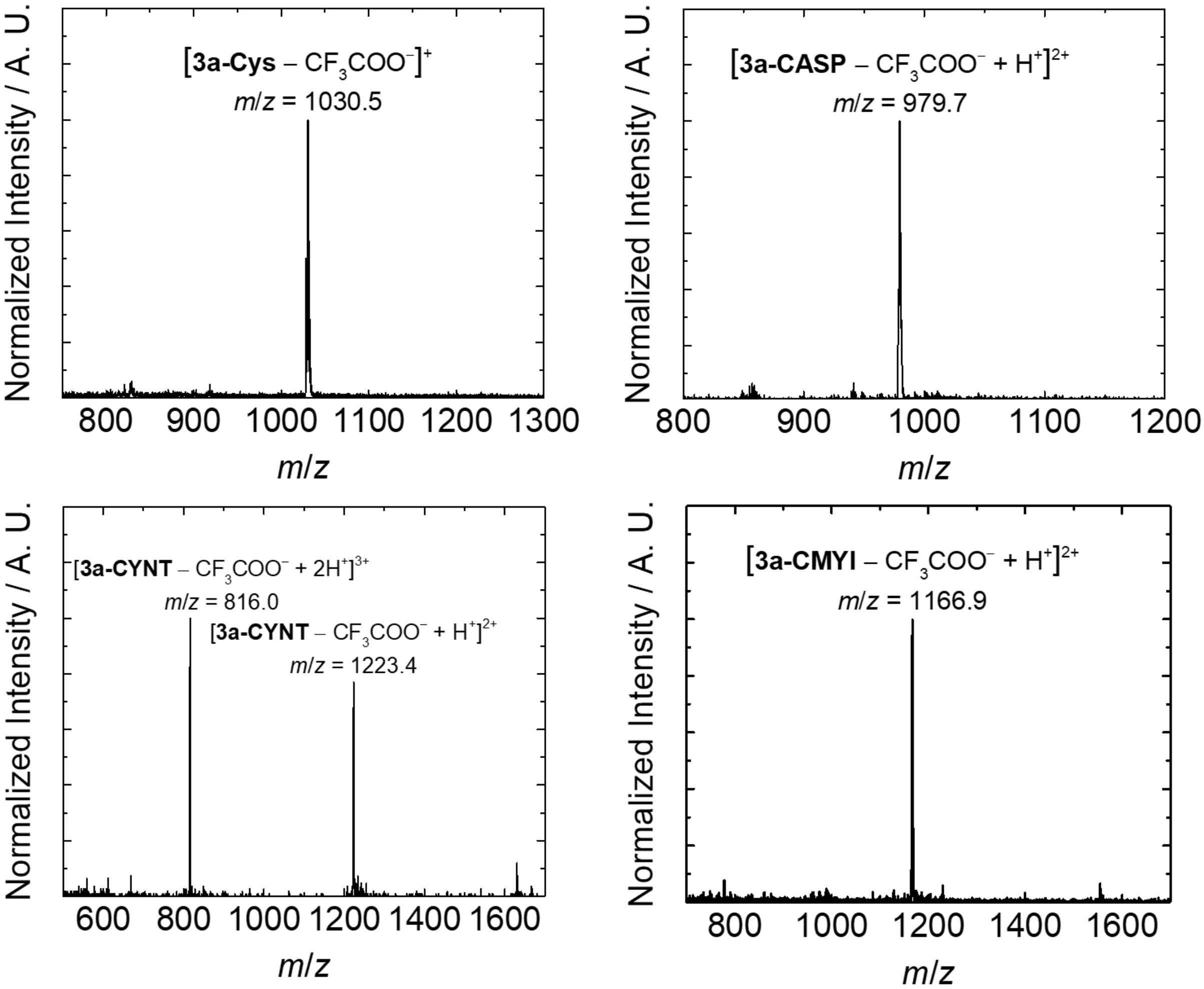

Figure S20. Normalized emission spectra of the peptide conjugates of complex **3a** in degassed potassium phosphate buffer (50 mM, pH 7.4)/MeOH (2:3, *v*/*v*) at 298 K.

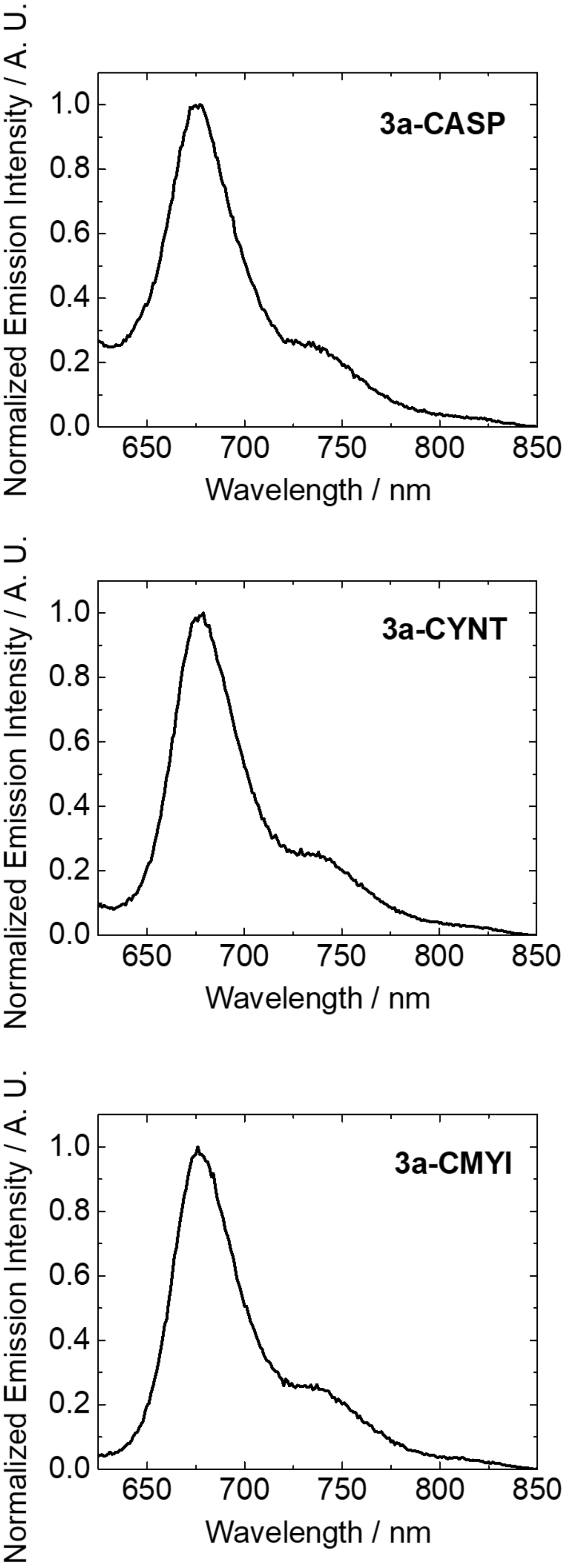

Figure S21. Relative amounts of iridium associated with an average MDA-MB-231 cell upon incubation with conjugate **3a-CASP** (10 μM, 4 h) at 37°C without or with preincubation of the cells at 4°C for 1 h. The uptake values at 37°C were taken as the reference.

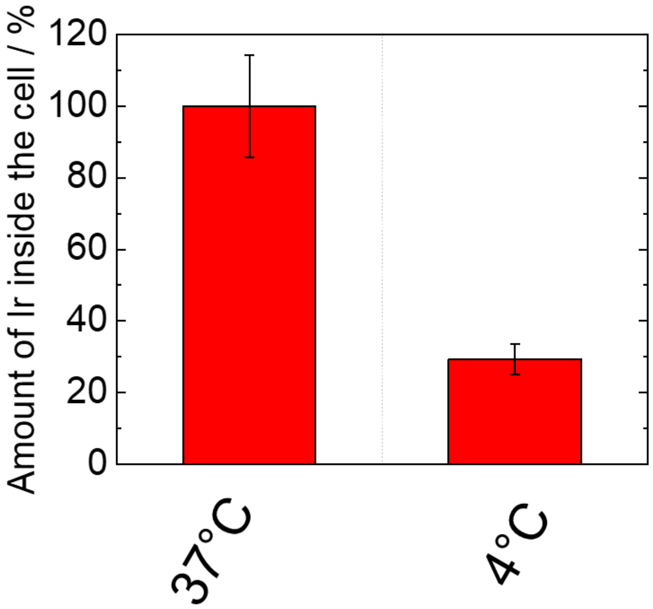

Figure S22. Relative amounts of iridium associated with an average MDA-MB-231 cell upon incubation with conjugate **3a-CASP** (10 μM, 16 h) at 37°C without or with preincubation of the cells with EIPA (50 μM, 1.5 h), Me-β-CD (5 mM, 1 h), or chlorpromazine (30 μM, 1 h). The uptake values at 37°C without pretreatment were taken as the reference.

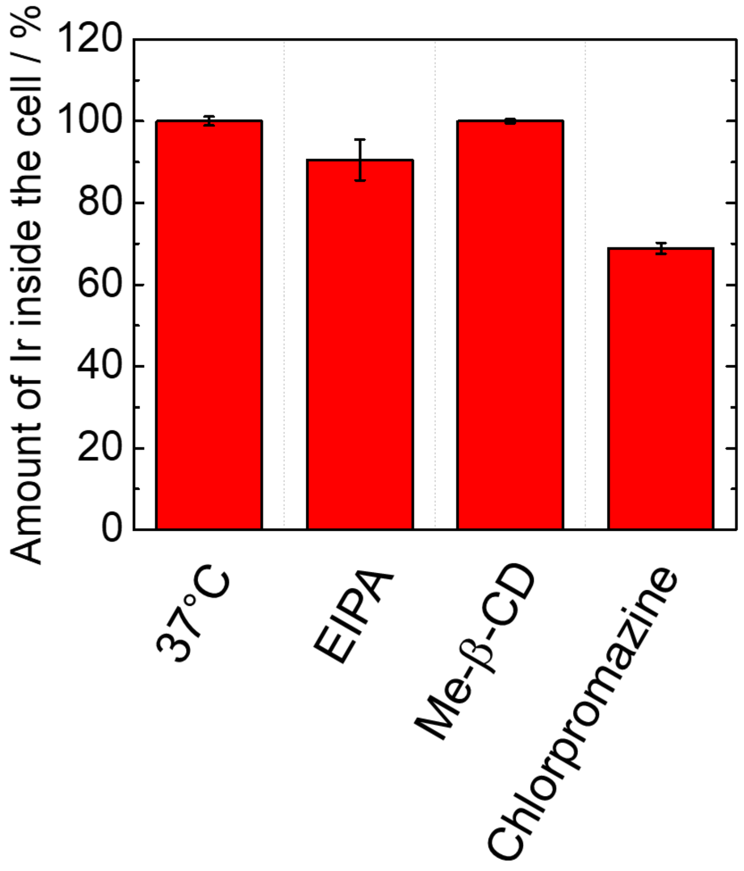

Figure S23. Relative amounts of iridium associated with an average MDA-MB-231 cell upon incubation with conjugate **3a-CYNT** (10 μM, 16 h) at 37°C without or with preincubation of the cells with acetazolamide (1 mM, 6 h). The uptake values at 37°C without pretreatment were taken as the reference.

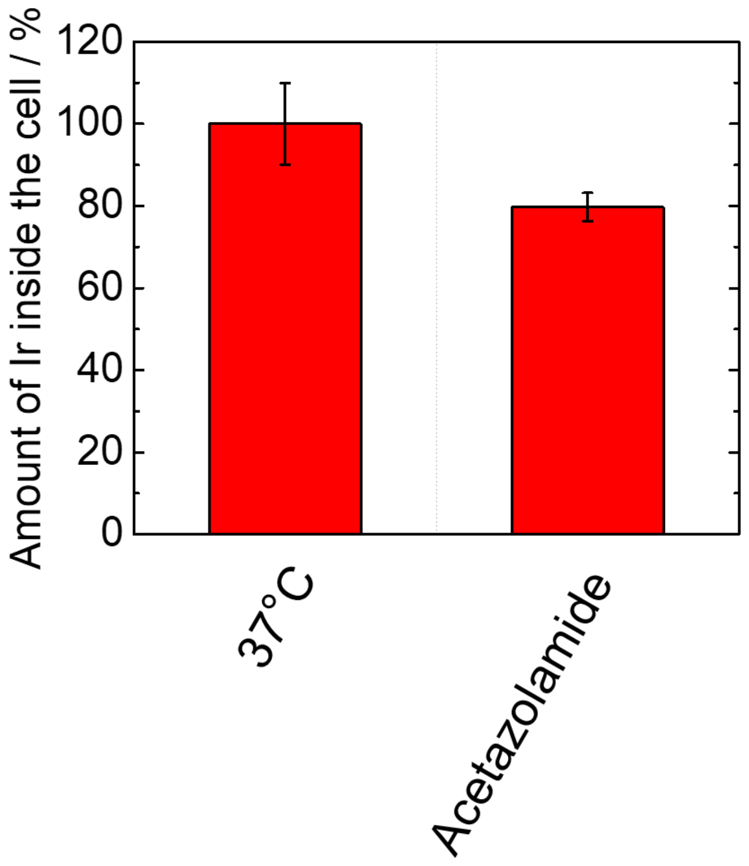

Figure S24. Relative amounts of iridium associated with an average MDA-MB-231 cell upon incubation with conjugate **3a-CMYI** (10 μM, 16 h) at 37°C without or with preincubation of the cells with gefitinib (50 μM, 1 h). The uptake values at 37°C without pretreatment were taken as the reference.

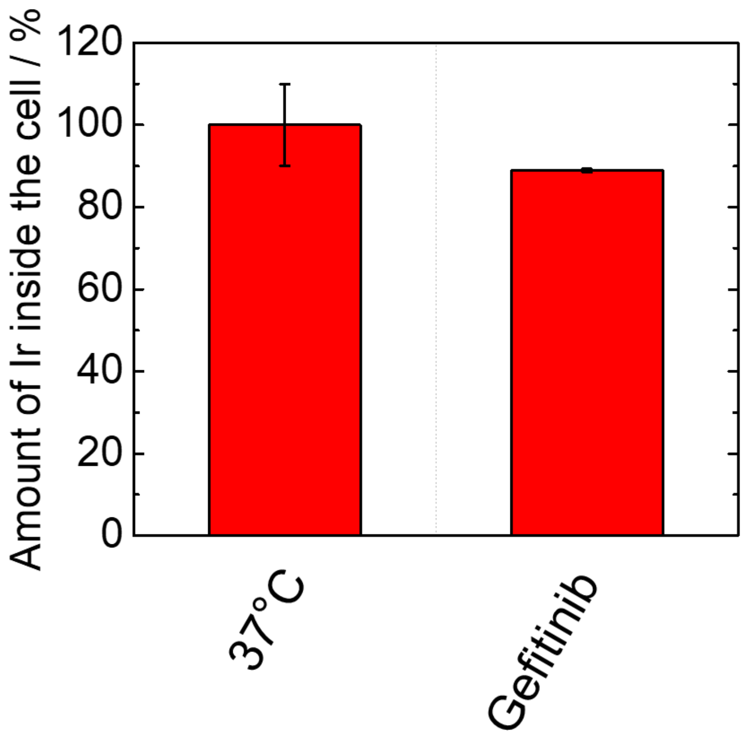

Figure S25. LSCM images of live MDA-MB-231 cells incubated with conjugates **3a-Cys**, **3a-CASP**, **3a-CYNT**, and **3a-CMYI** (10 μM, 16 h, λ_{ex} = 488 nm, λ_{em} = 650 – 750 nm), and further incubated with MitoTracker Green (100 nM, 20 min, λ_{ex} = 488 nm, λ_{em} = 505 – 525 nm). PCC = 0.43 (**3a-Cys**), 0.46 (**3a-CASP**), 0.36 (**3a-CYNT**), and 0.28 (**3a-CMYI**). Scale bars = 25 μm.

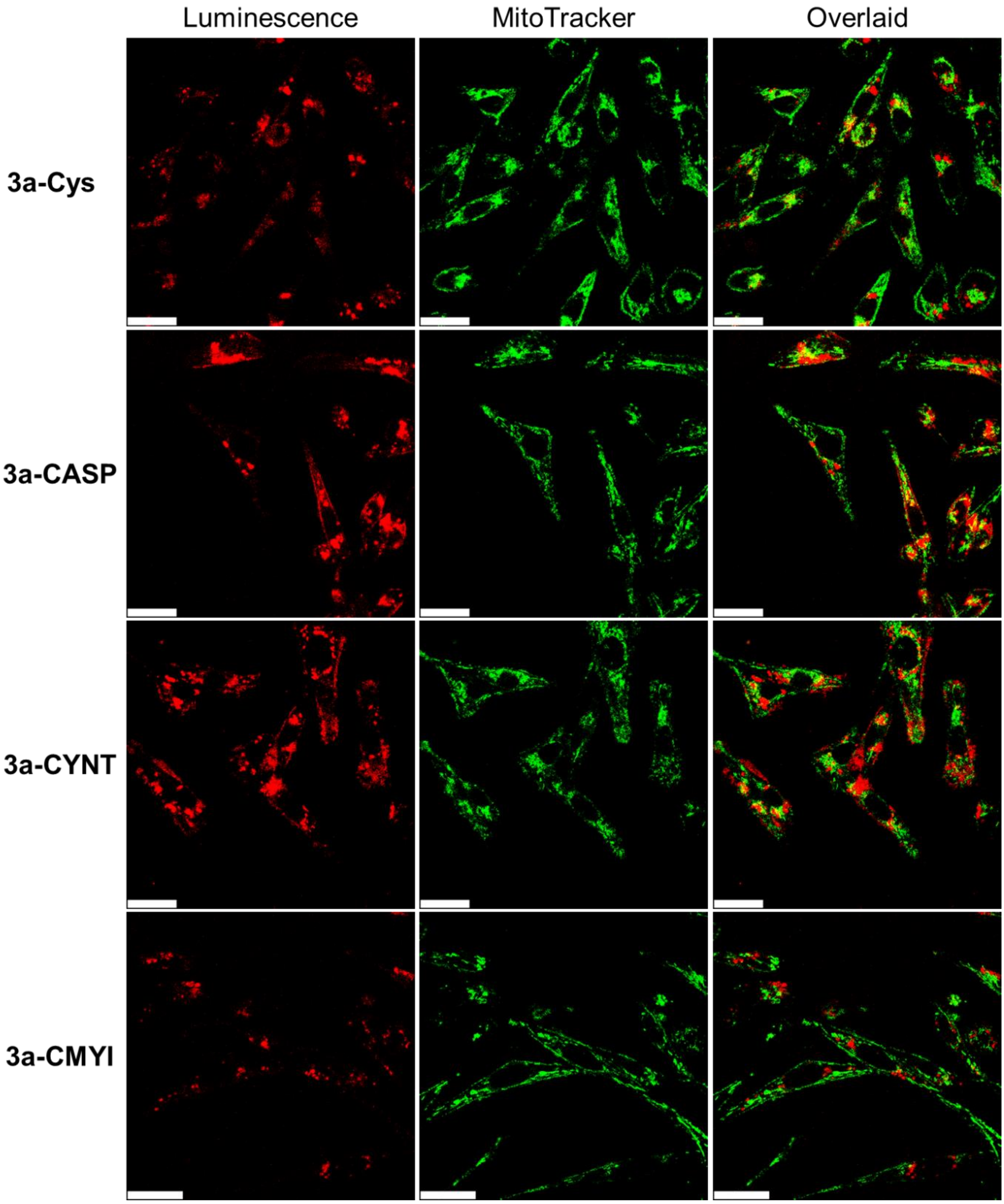

Figure S26. Comparison of the geometric structures of the S_0 state (black), ^{3}MLCT state (red), ^{3}MC state (magenta), ^{3}MC/S_0 MECP (lime), ^{3}IL state (blue), and ^{3}IL/S_0 MECP (orange) for complexes **1a-Me**, **1a-Cys**, and **1b**. The root-mean-square deviations (Å) with respect to the geometric structure of S_0 state are provided. Hydrogen atoms are removed for clarity.

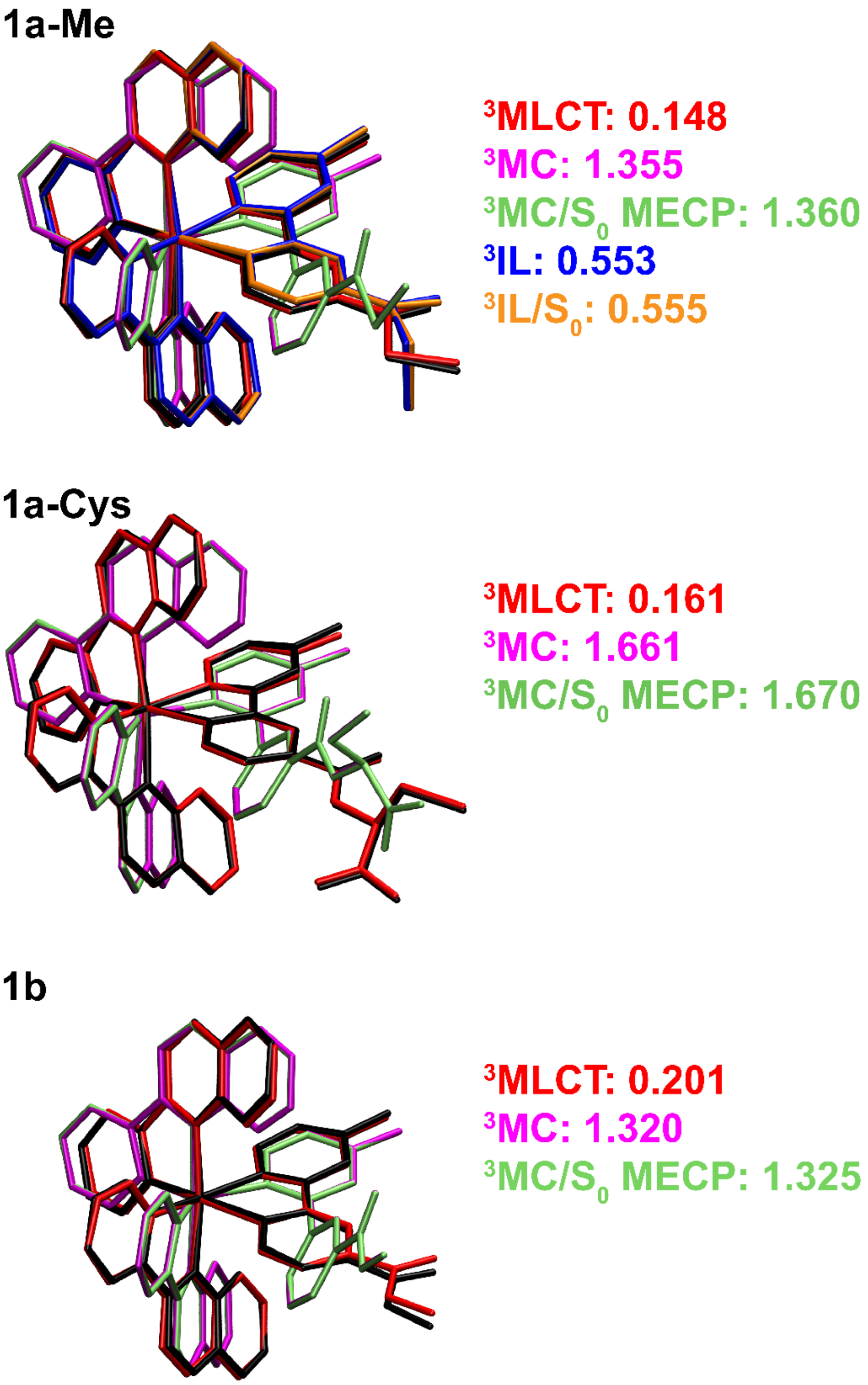

Figure S27. Schematic diagrams of potential energy profiles (eV) along the nuclear coordinate (*Q*) for complexes (a) **1a-Me** and (b) **1b**. Relative energies of the optimized S_0, ^{3}MLCT, ^{3}IL, and ^{3}MC structures on the S_0 and T_1 surfaces are provided.

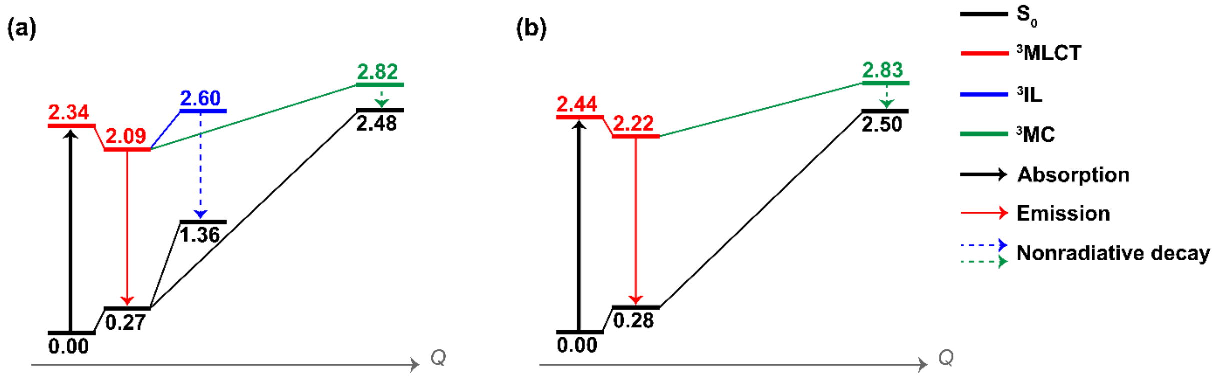

Figure S28. ^{1}H NMR spectrum of bpy-COSBn in $(CD_3)_2CO$ at 298 K.

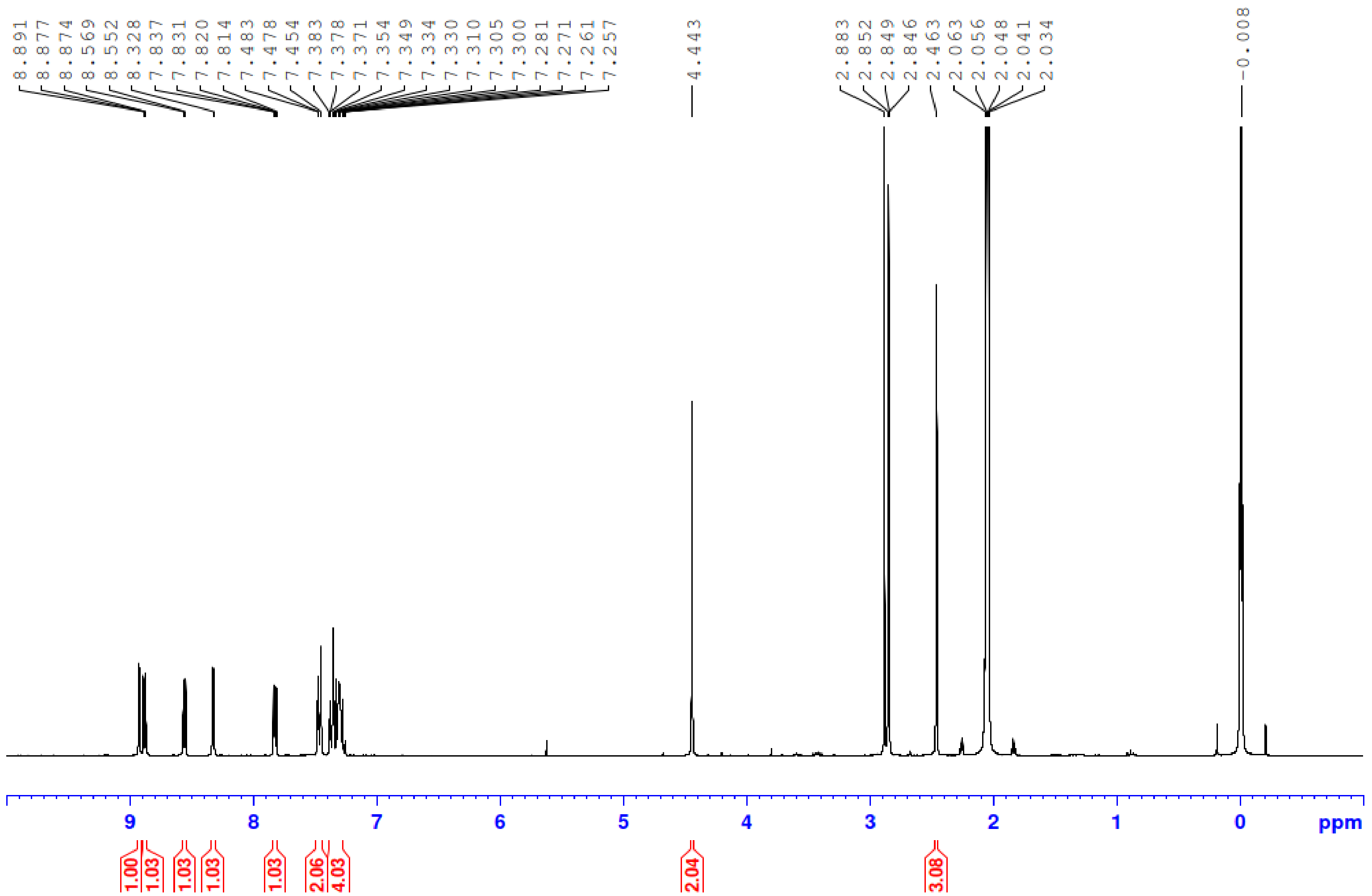

Figure S29. ^{1}H NMR spectrum of complex **1a** in $(CD_3)_2CO$ at 298 K.

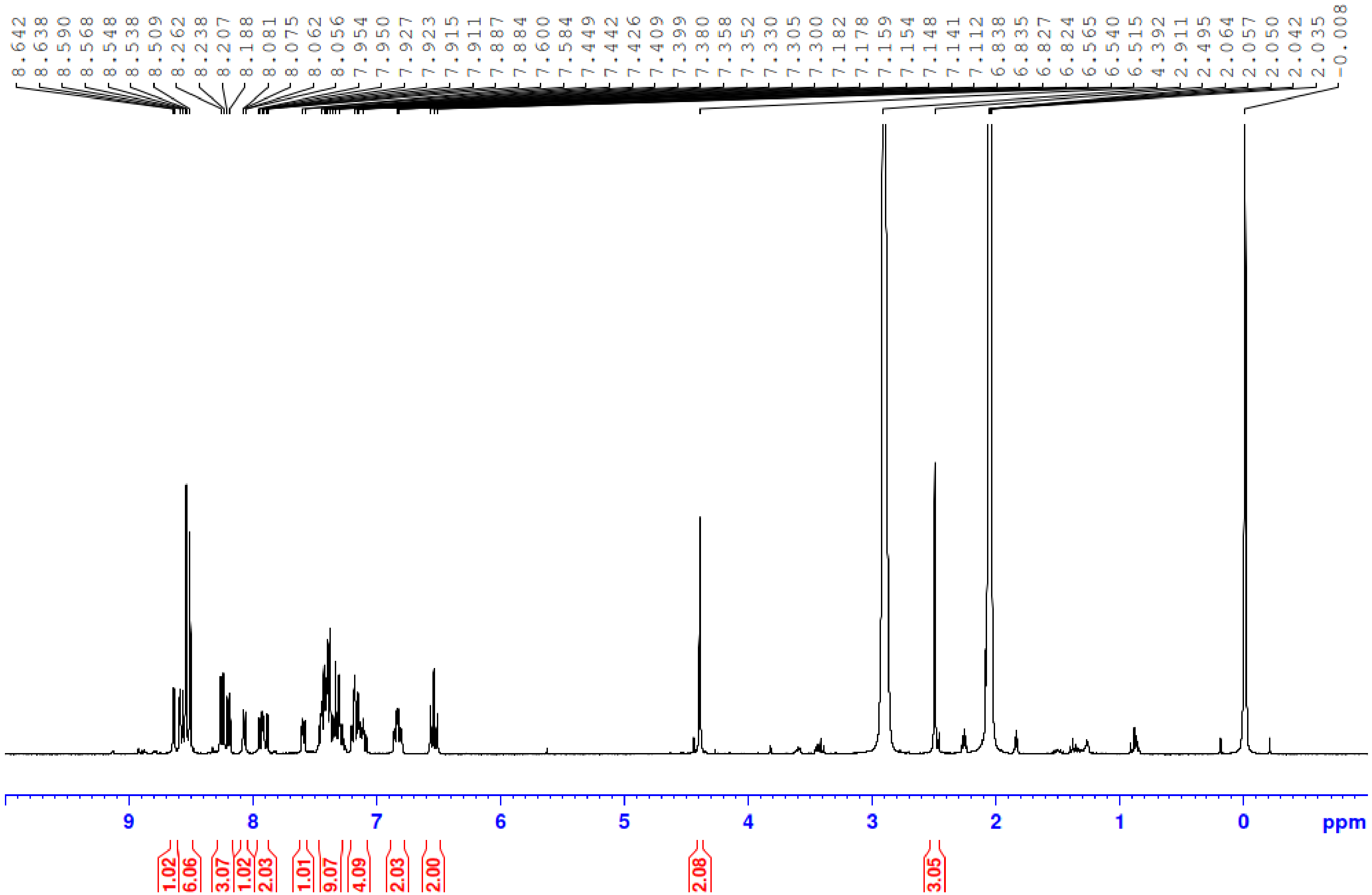

Figure S30. ^{13}C NMR spectrum of complex **1a** in $(CD_3)_2CO$ at 298 K.

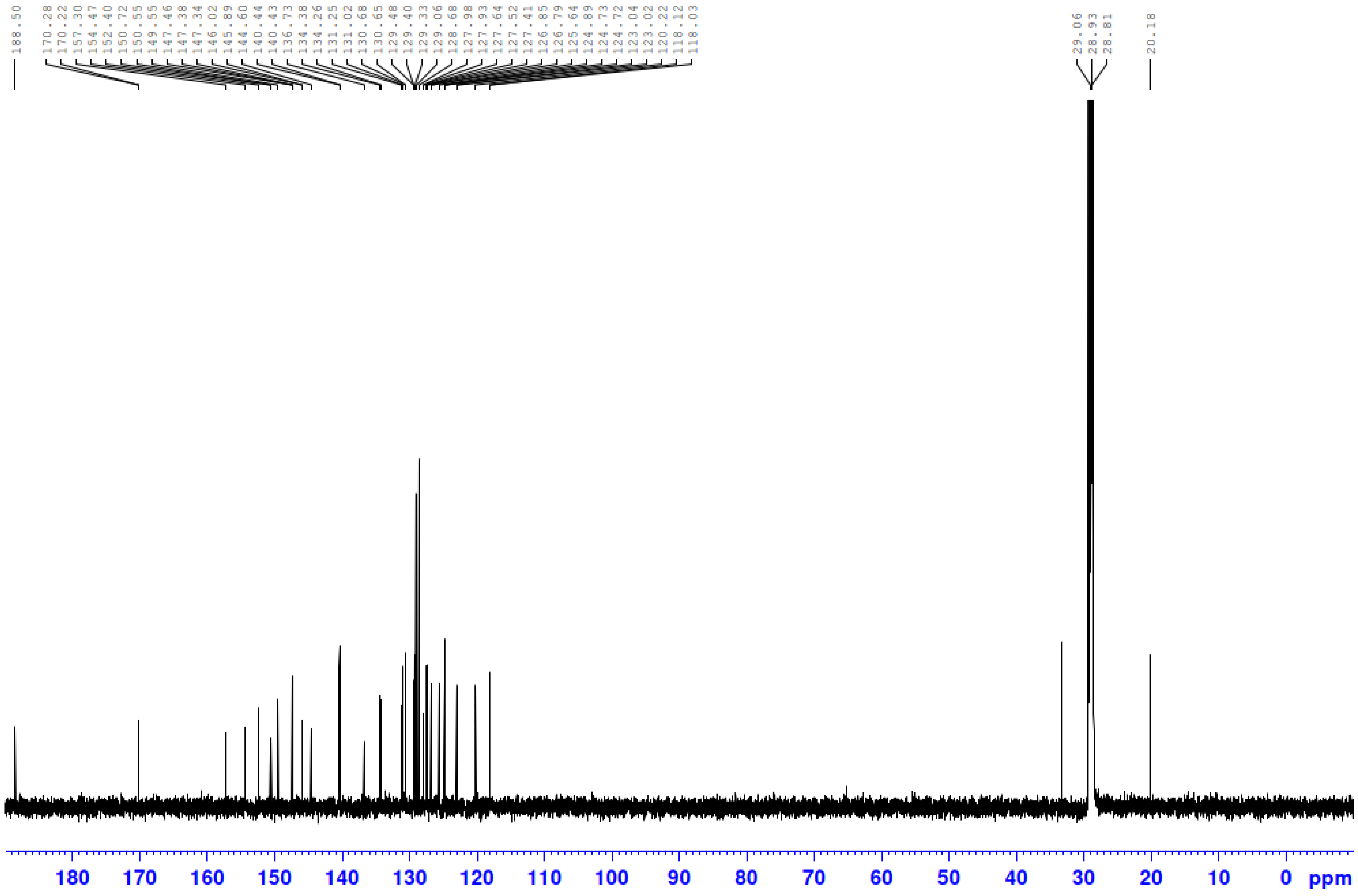

Figure S31. (a) Experimental and (b) simulated HR-ESI mass spectra of complex **1a** in CH_3CN.

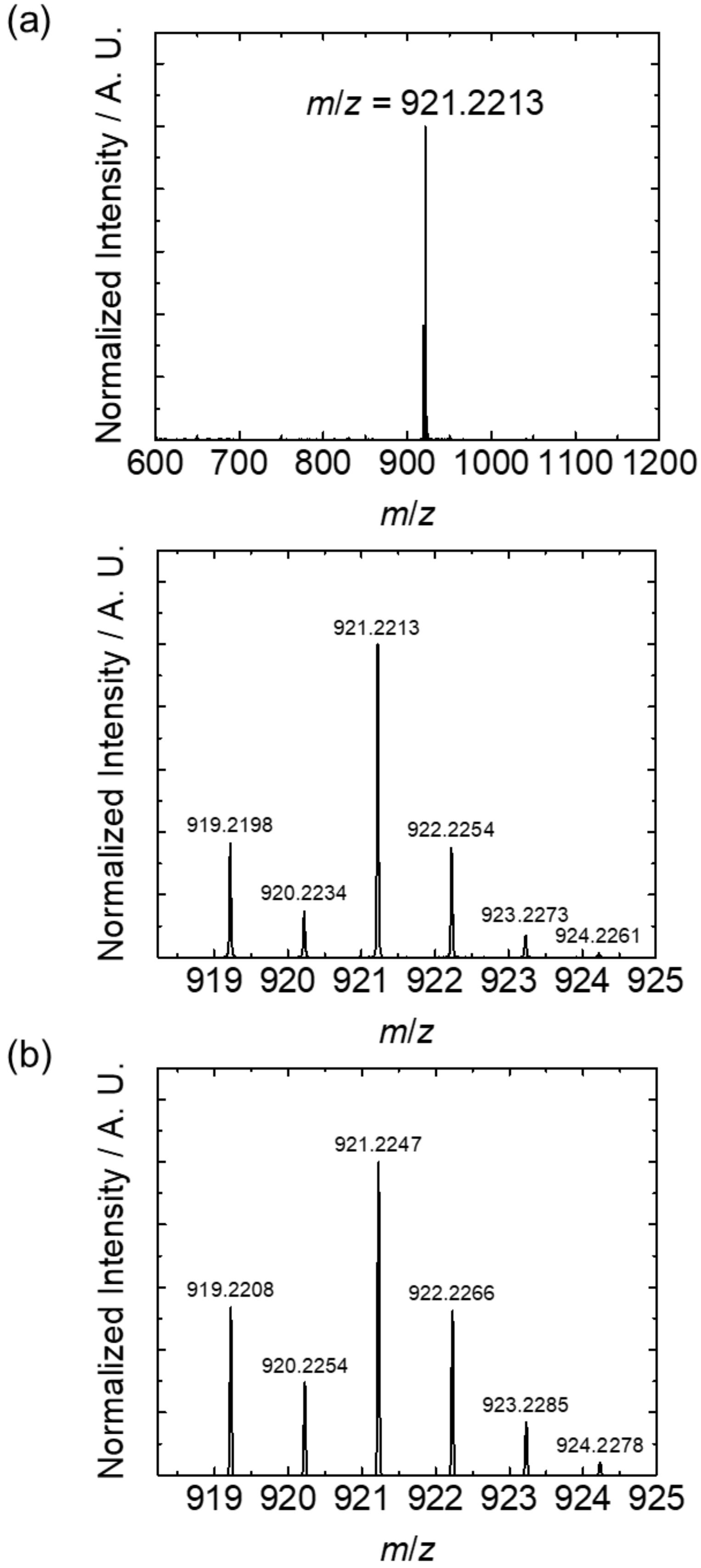

Figure S32. ^{1}H NMR spectrum of complex **1b** in $(CD_3)_2CO$ at 298 K.

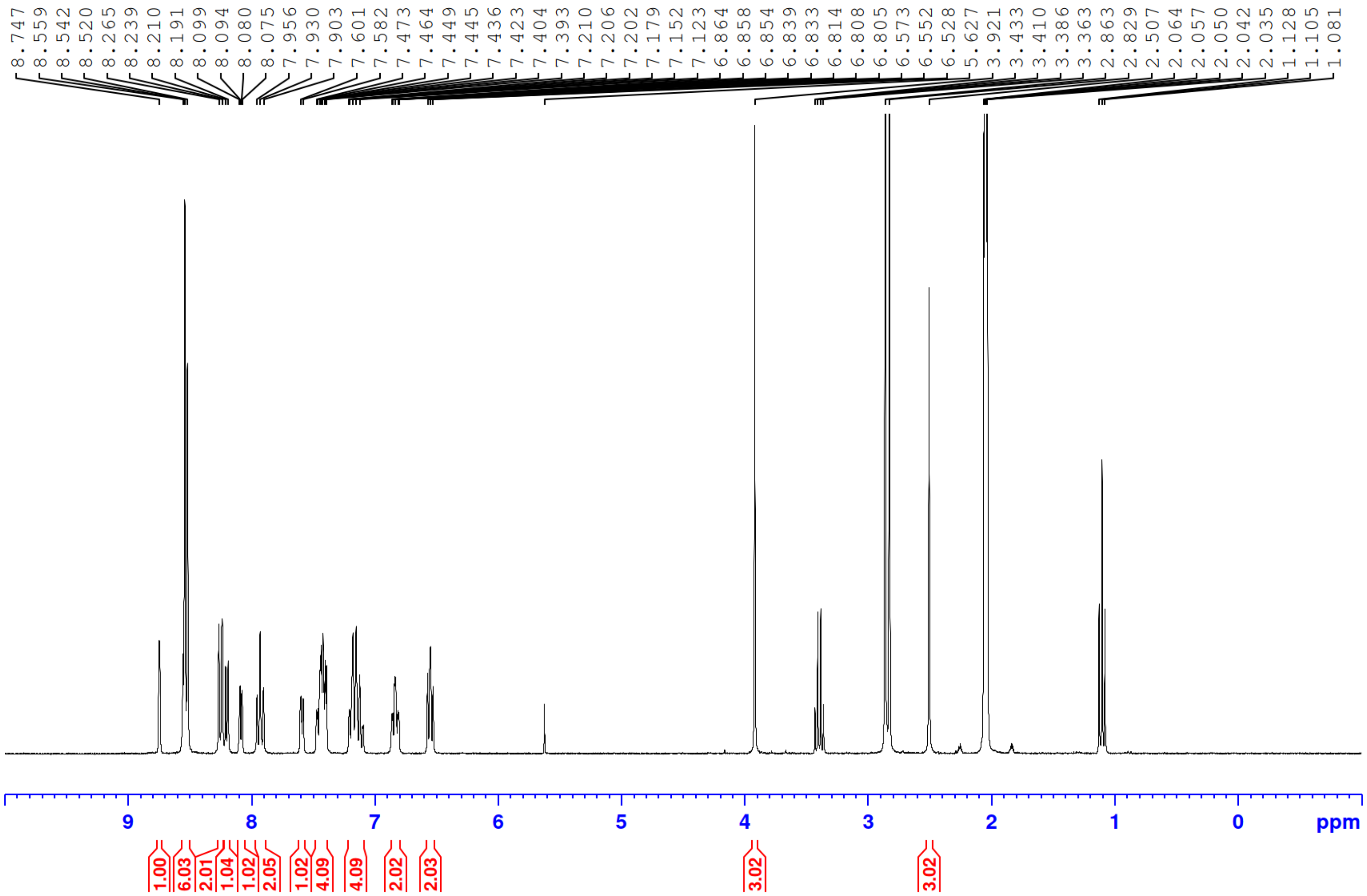

Figure S33. ^{13}C NMR spectrum of complex **1b** in $(CD_3)_2CO$ at 298 K.

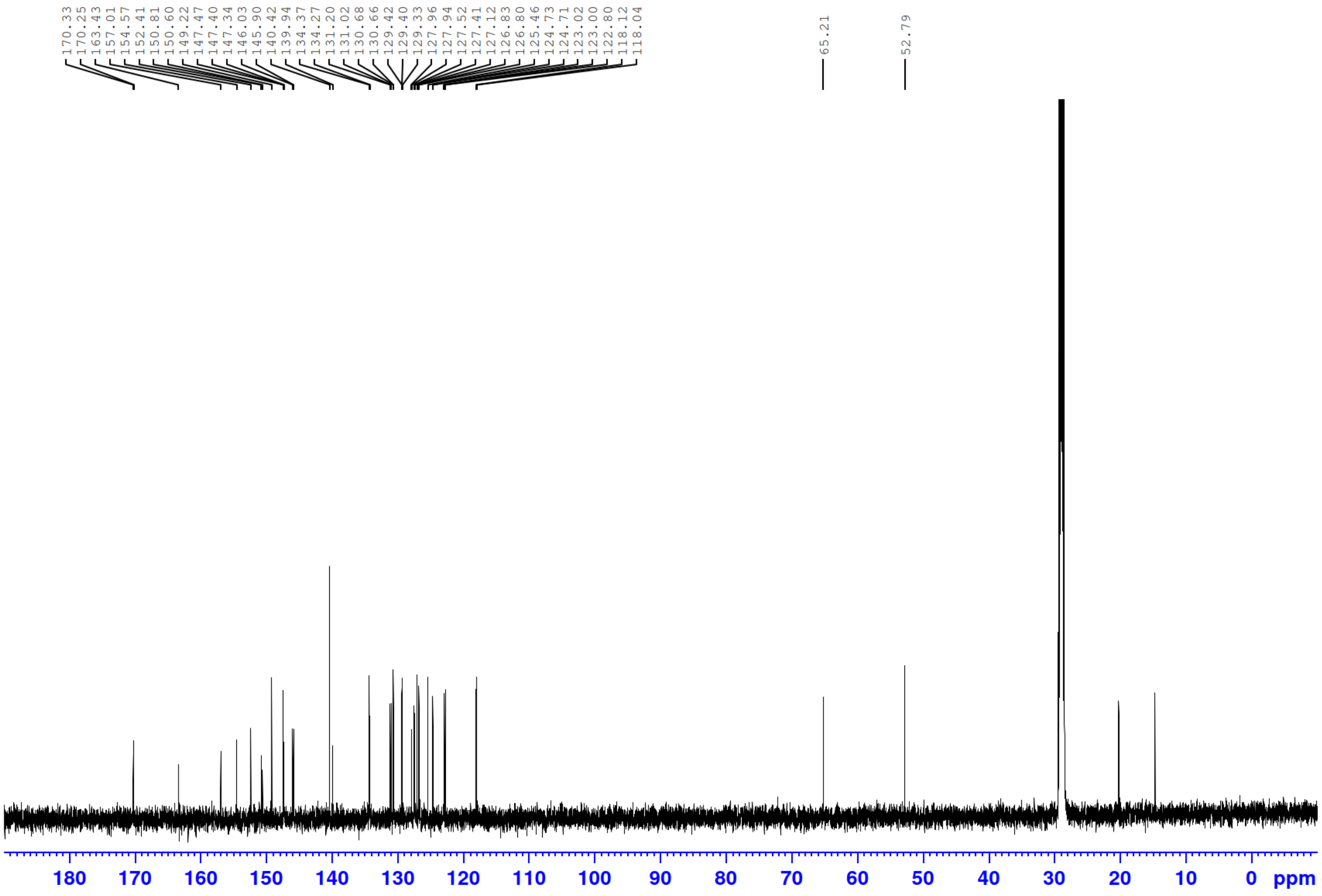

Figure S34. (a) Experimental and (b) simulated HR-ESI mass spectra of complex **1b** in CH_3CN.

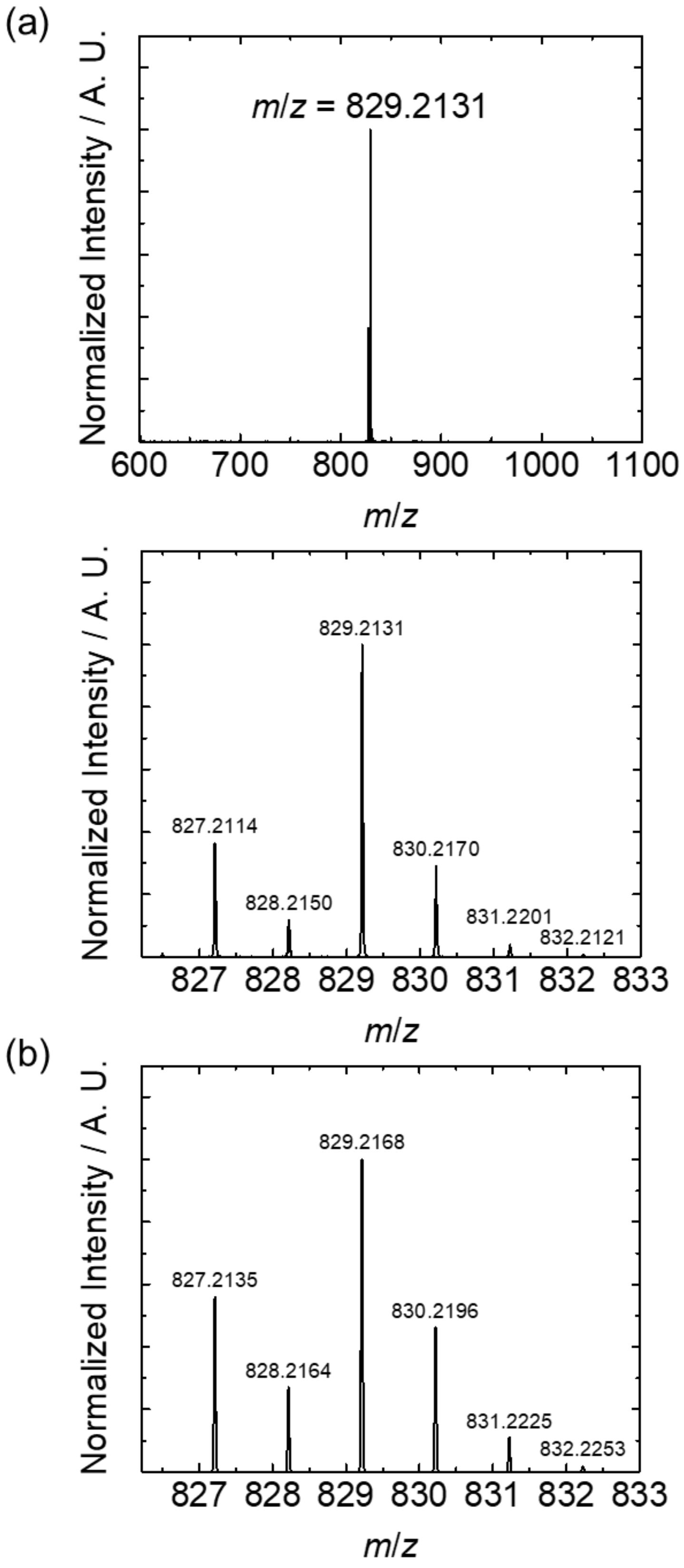

Figure S35. ^{1}H NMR spectrum of complex **2a** in $(CD_3)_2CO$ at 298 K.

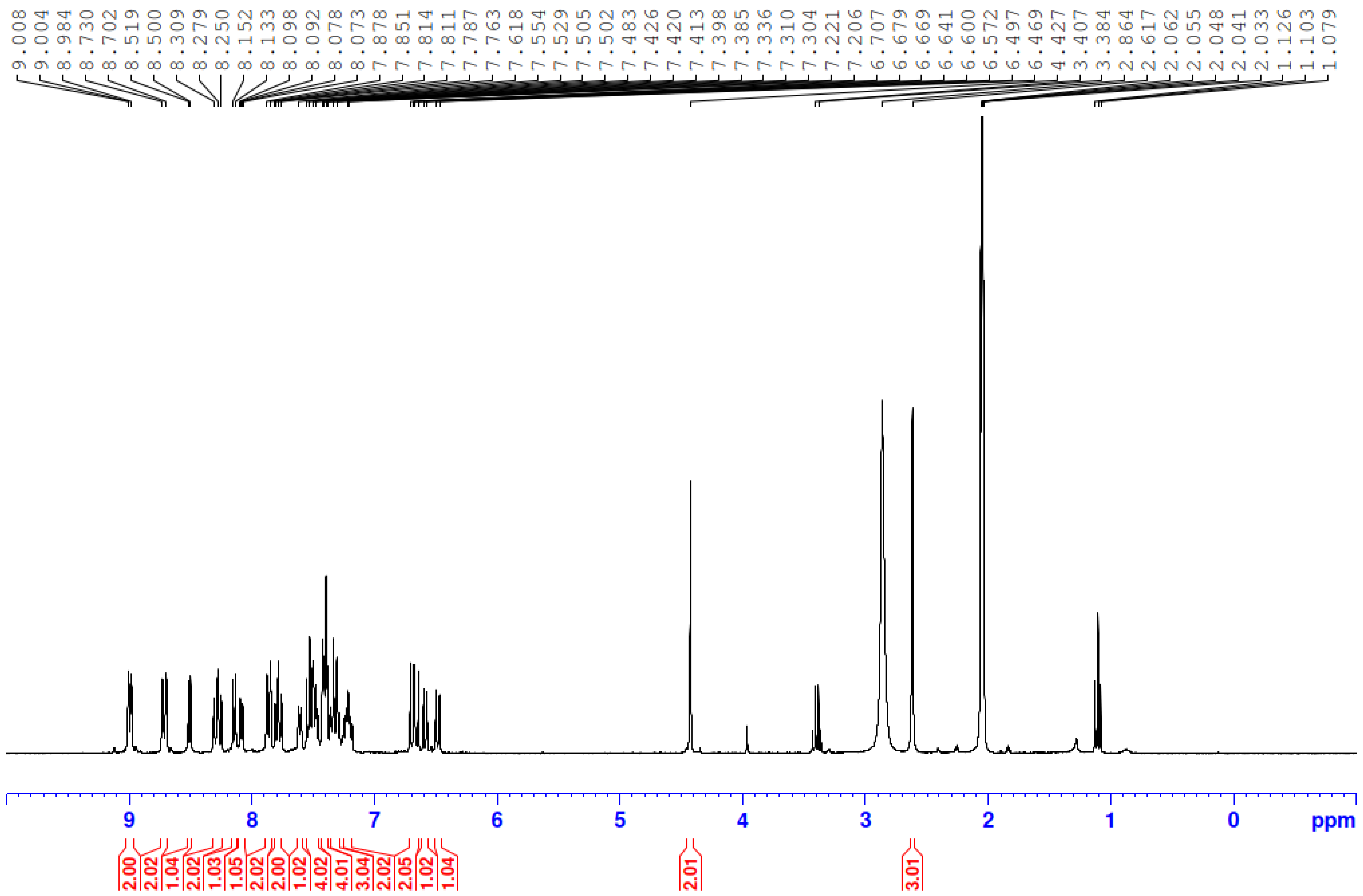

Figure S36. ^{13}C NMR spectrum of complex **2a** in $(CD_3)_2CO$ at 298 K.

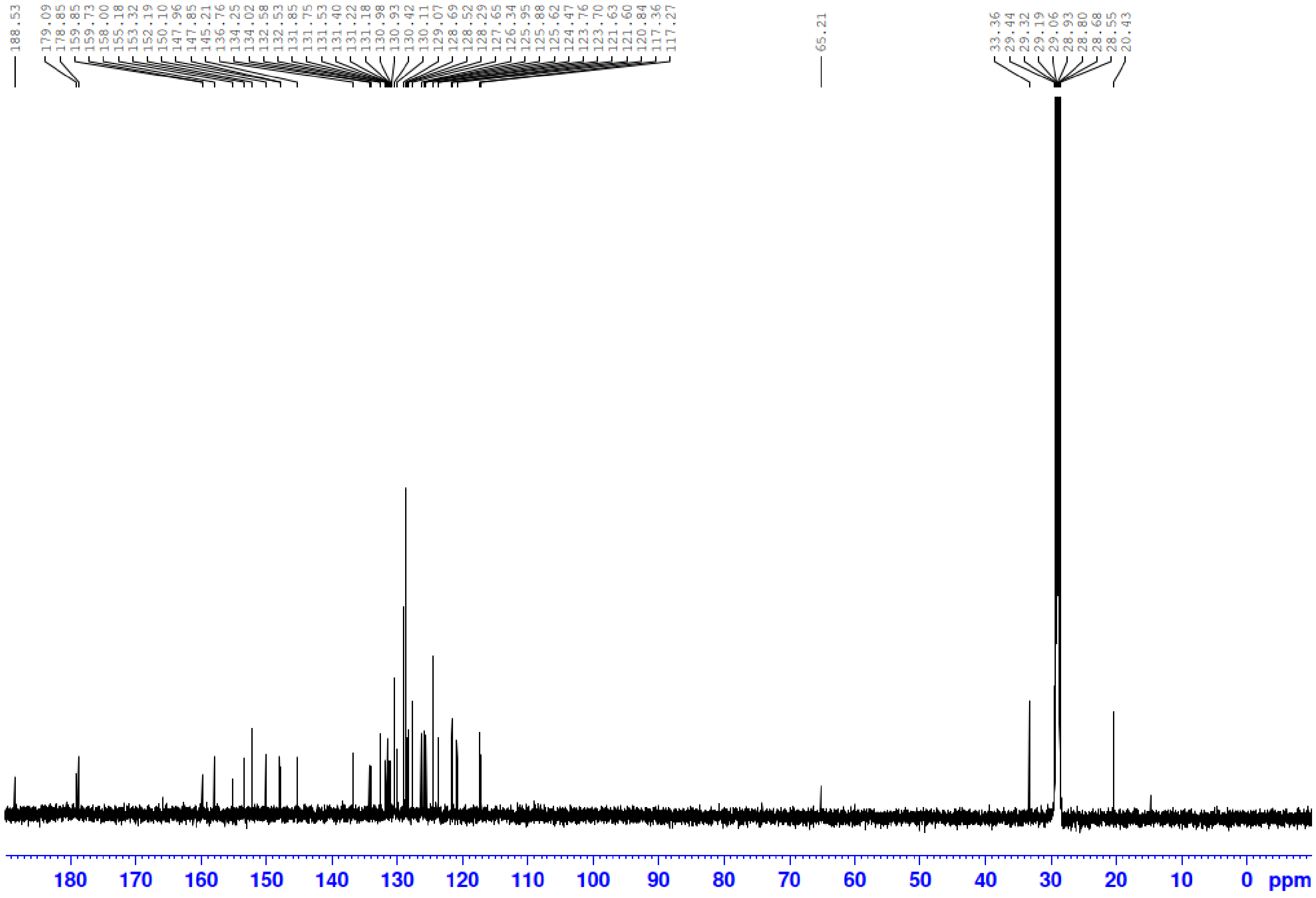

Figure S37. (a) Experimental and (b) simulated HR-ESI mass spectra of complex **2a** in CH_3CN.

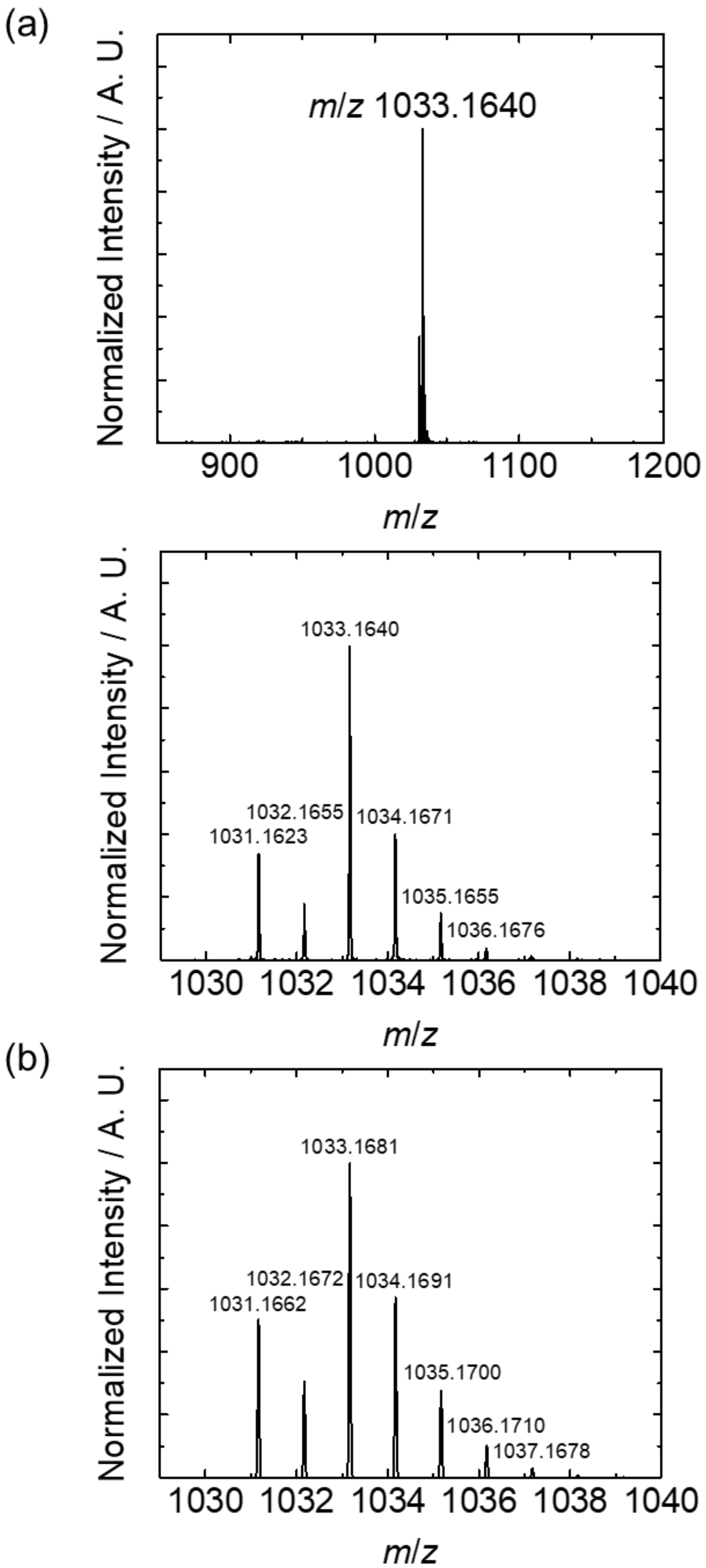

Figure S38. ^{1}H NMR spectrum of complex **2b** in $(CD_3)_2CO$ at 298 K.

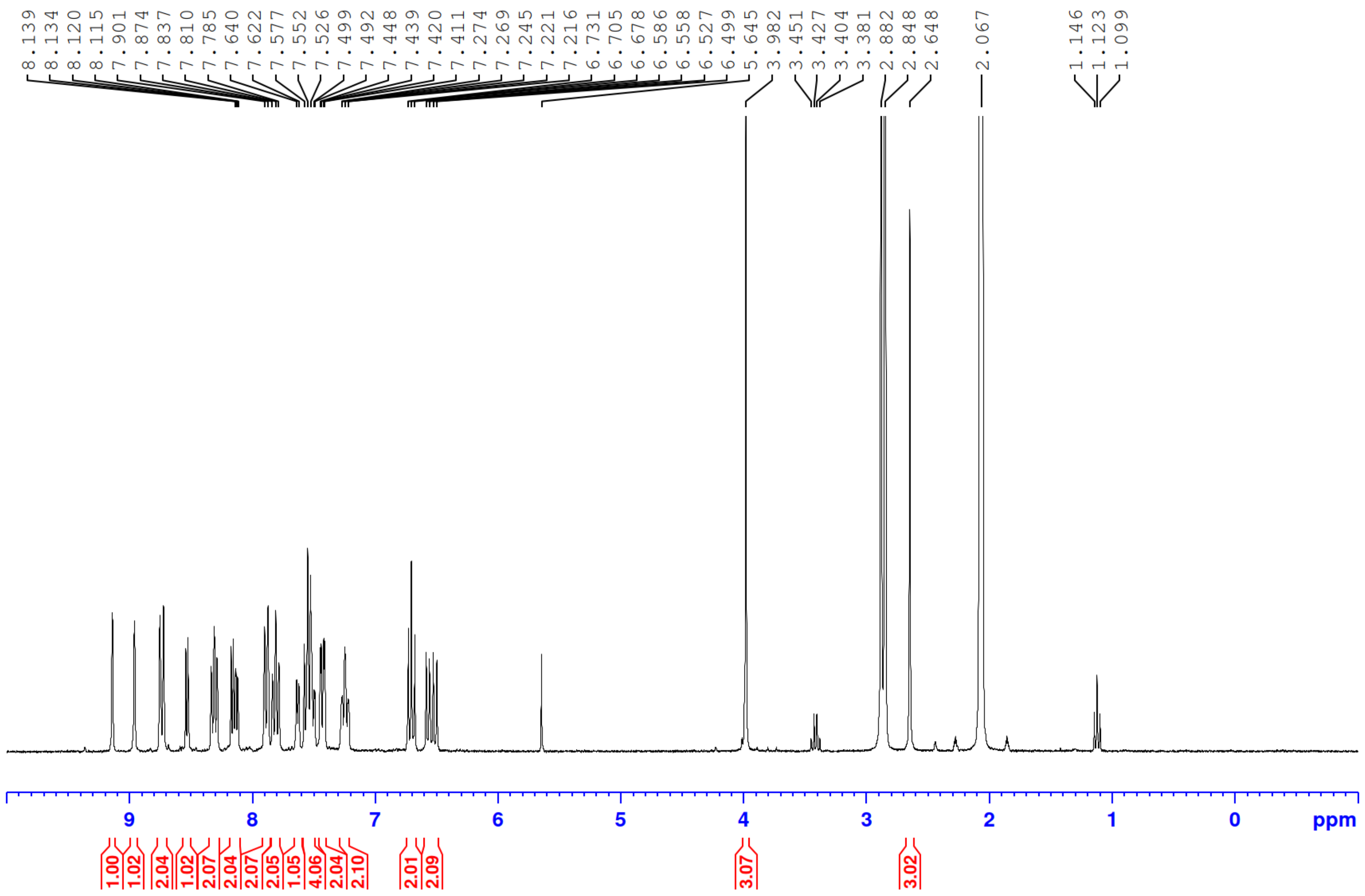

Figure S39. ^{13}C NMR spectrum of complex **2b** in $(CD_3)_2CO$ at 298 K.

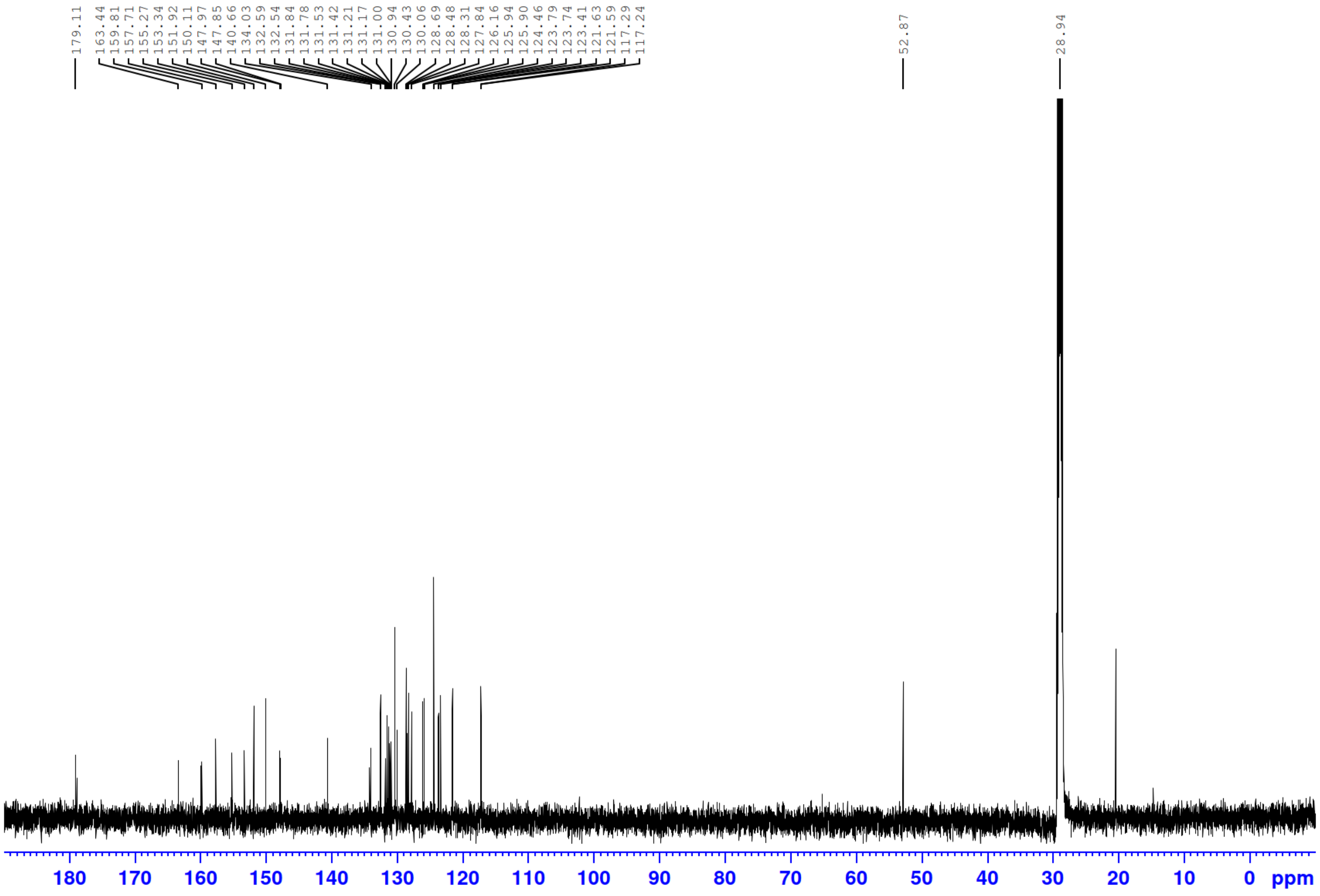

Figure S40. (a) Experimental and (b) simulated HR-ESI mass spectra of complex **2b** in CH_3CN.

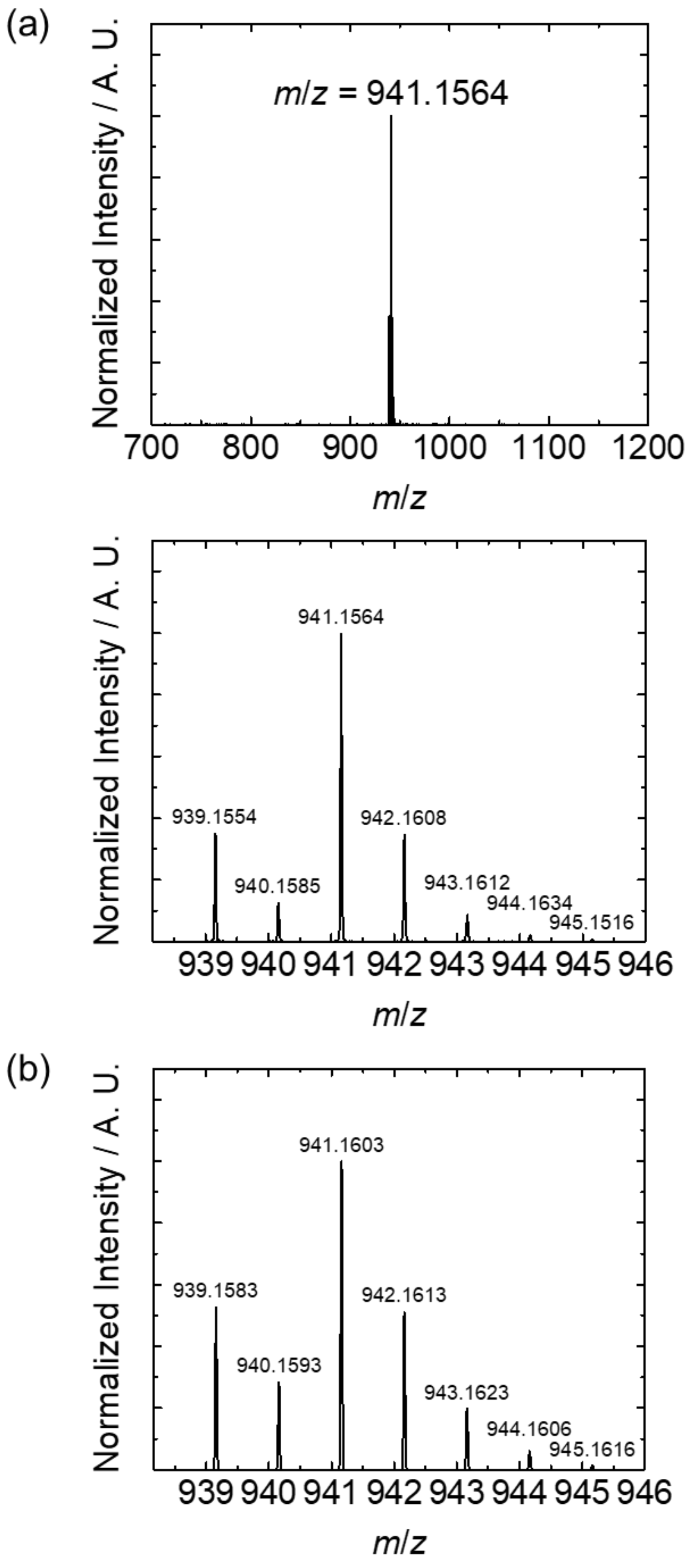

Figure S41. ^{1}H NMR spectrum of complex **3a** in $(CD_3)_2CO$ at 298 K.

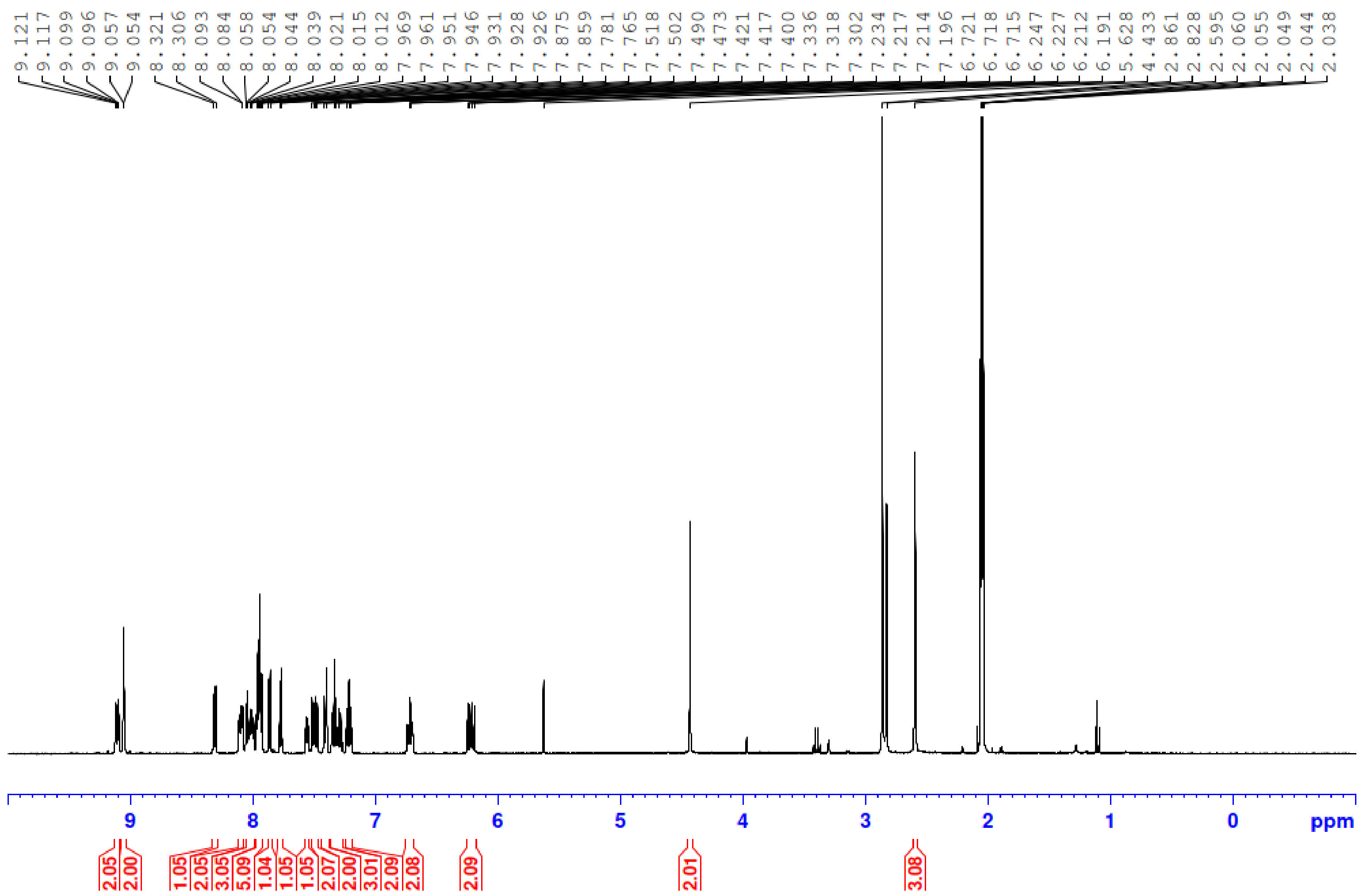

Figure S42. ^{13}C NMR spectrum of complex **3a** in $(CD_3)_2CO$ at 298 K.

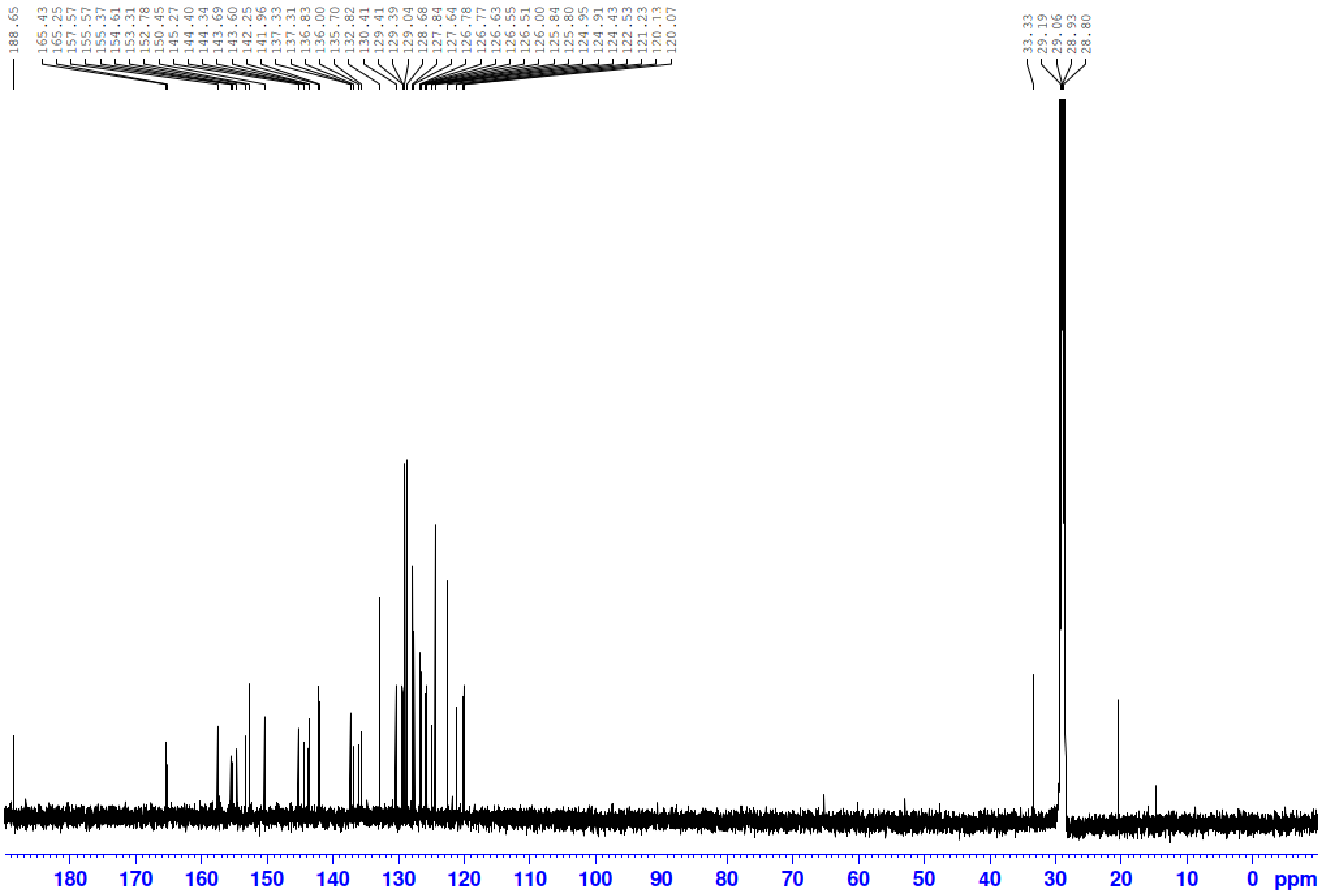

Figure S43. (a) Experimental and (b) simulated HR-ESI mass spectra of complex **3a** in CH_3CN.

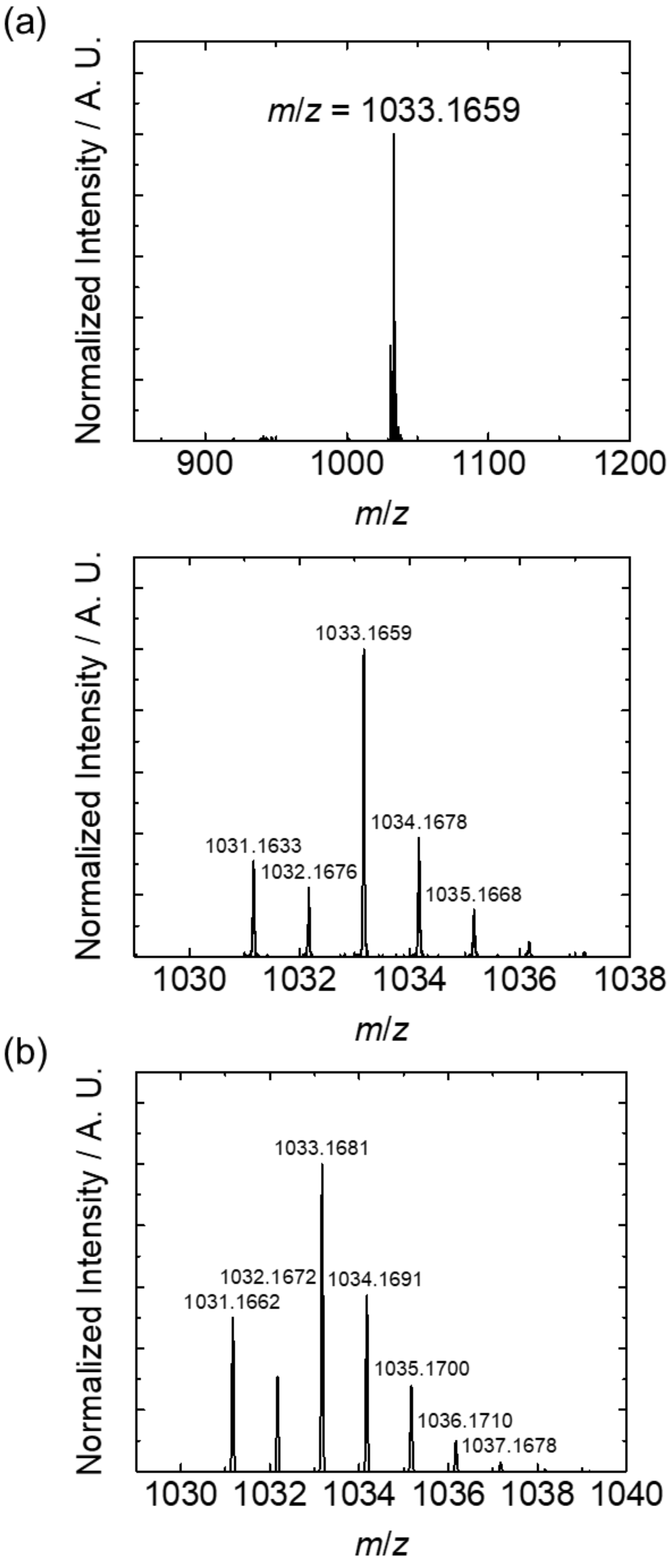

Figure S44. ^{1}H NMR spectrum of complex **3b** in $(CD_3)_2CO$ at 298 K.

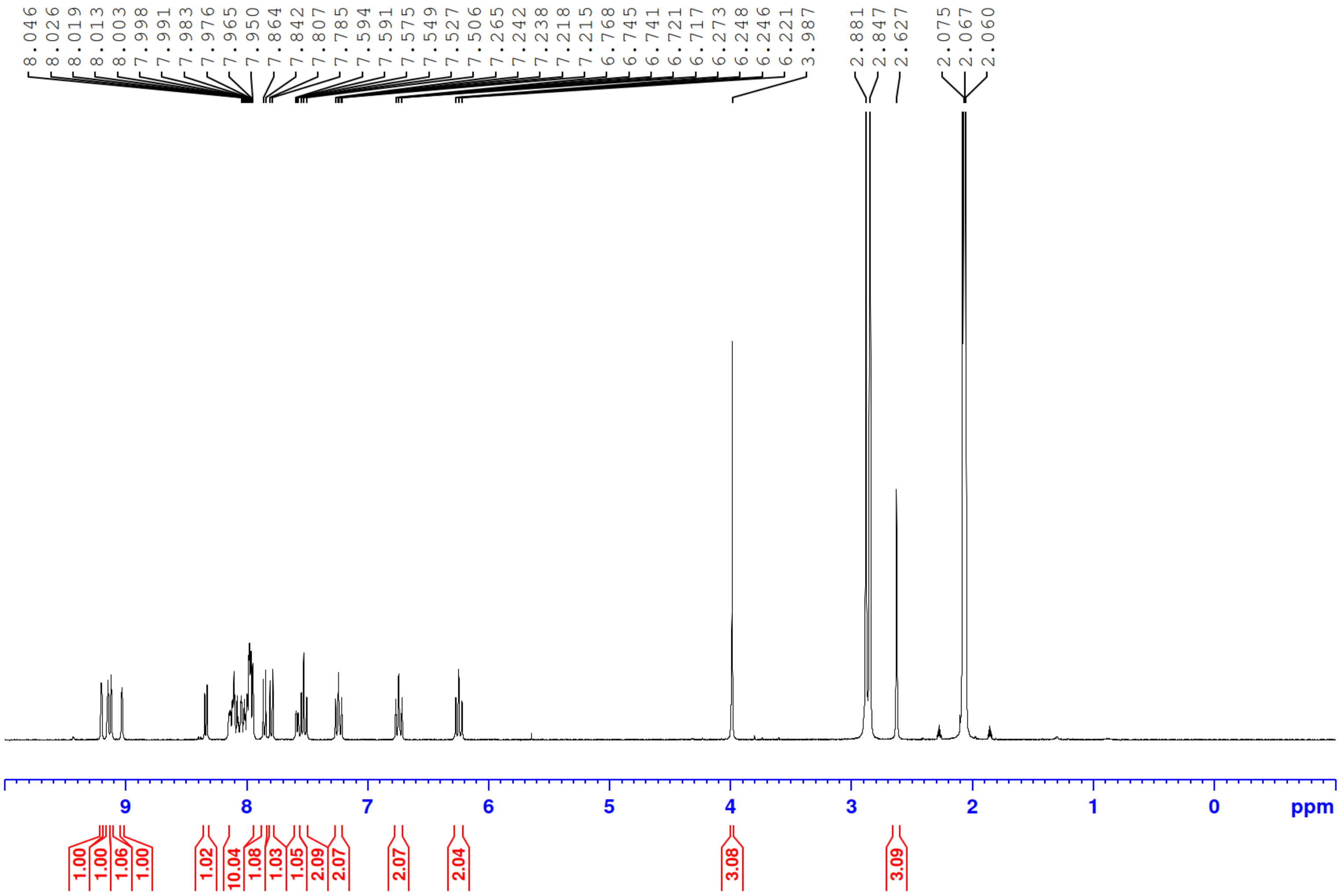

Figure S45. ^{13}C NMR spectrum of complex **3b** in $(CD_3)_2CO$ at 298 K.

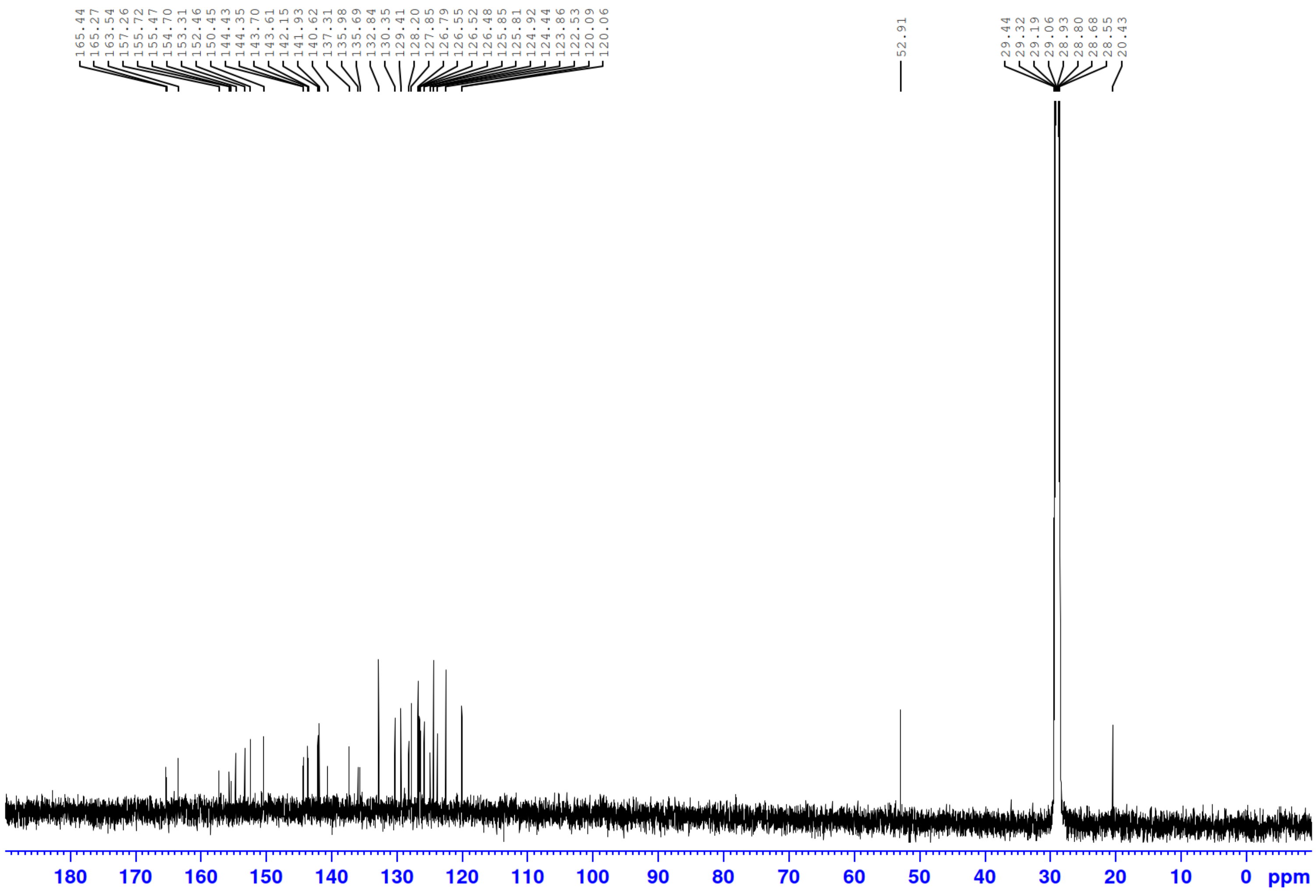

Figure S46. (a) Experimental and (b) simulated HR-ESI mass spectra of complex **3b** in CH_3CN.

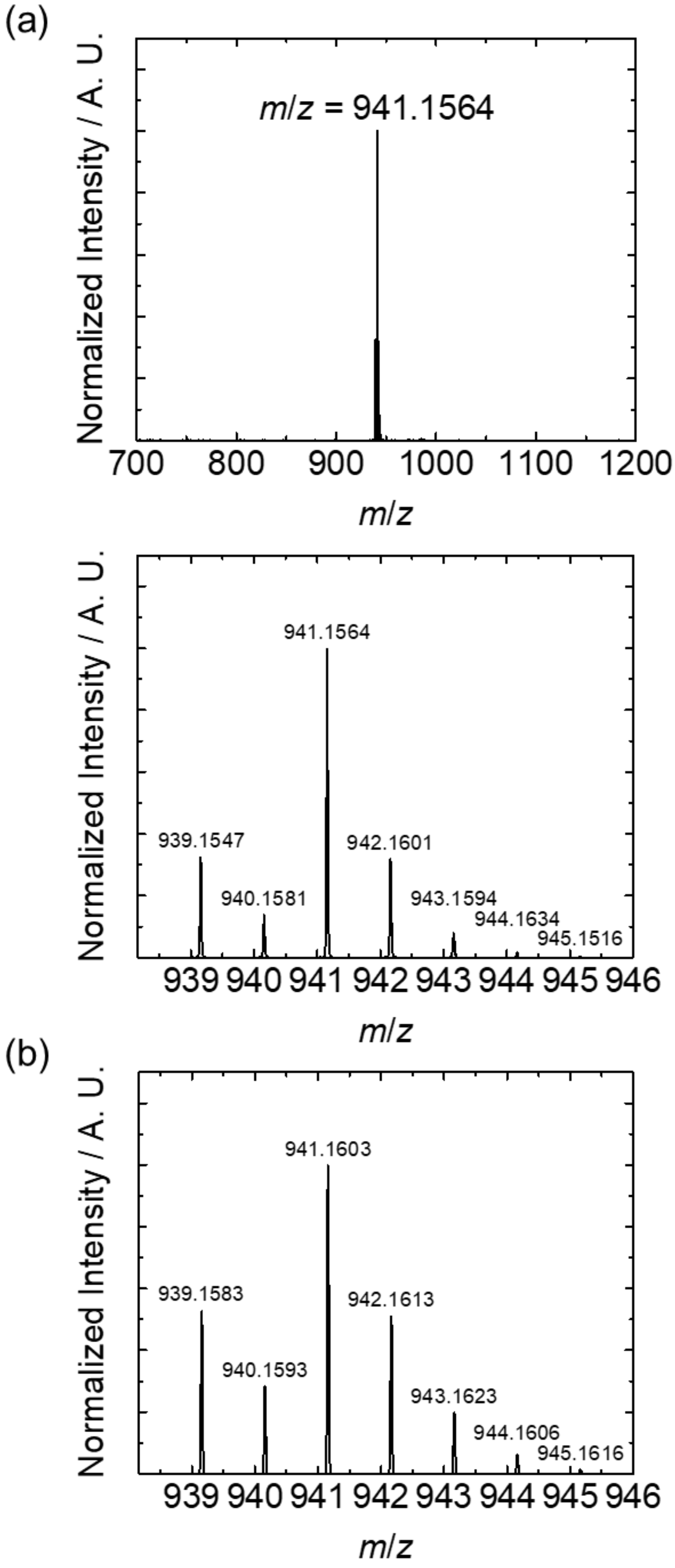

Figure S47. ^{1}H NMR spectrum of complex **4a** in $(CD_3)_2CO$ at 298 K.

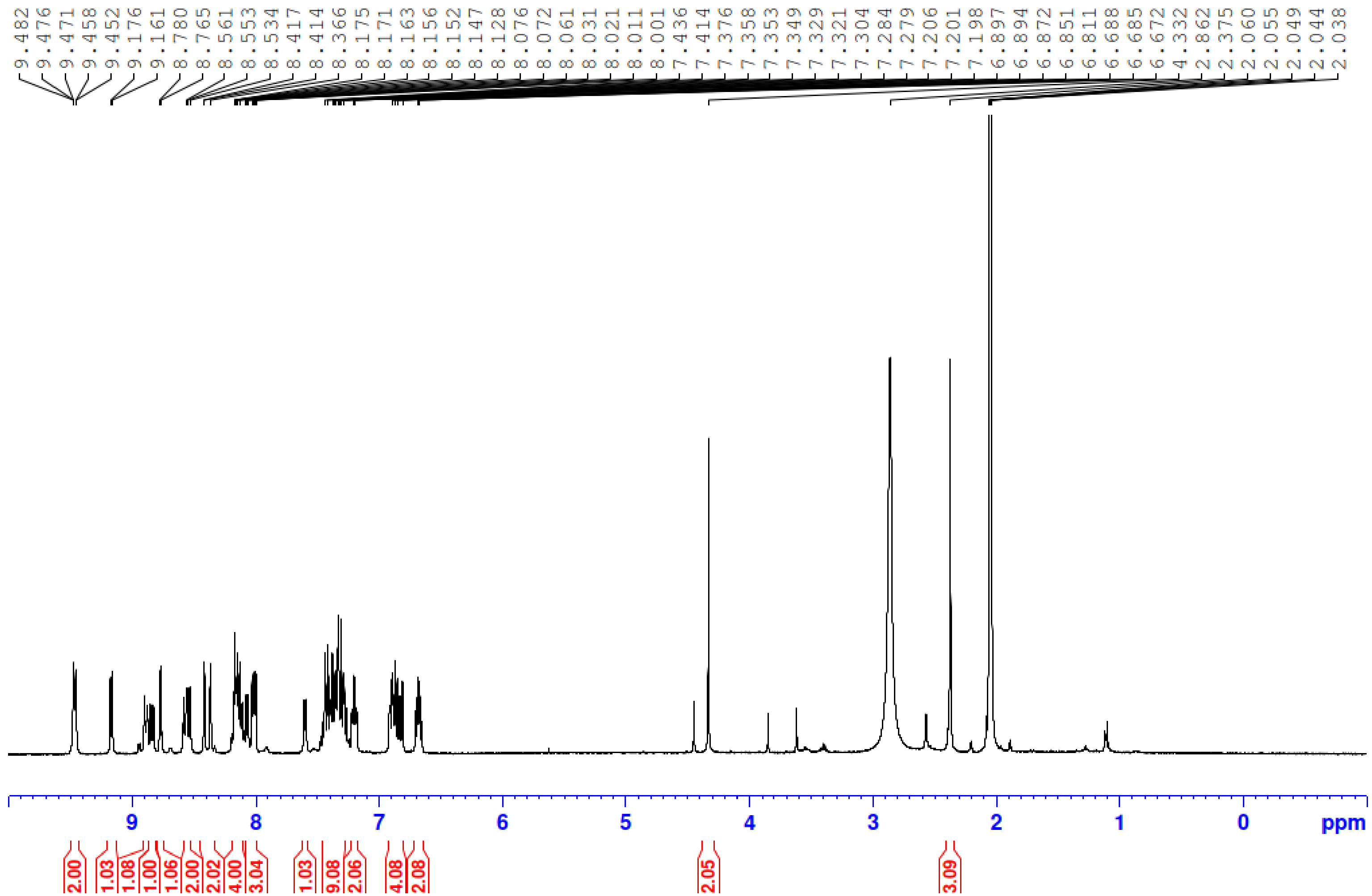

Figure S48. ^{13}C NMR spectrum of complex **4a** in $(CD_3)_2CO$ at 298 K.

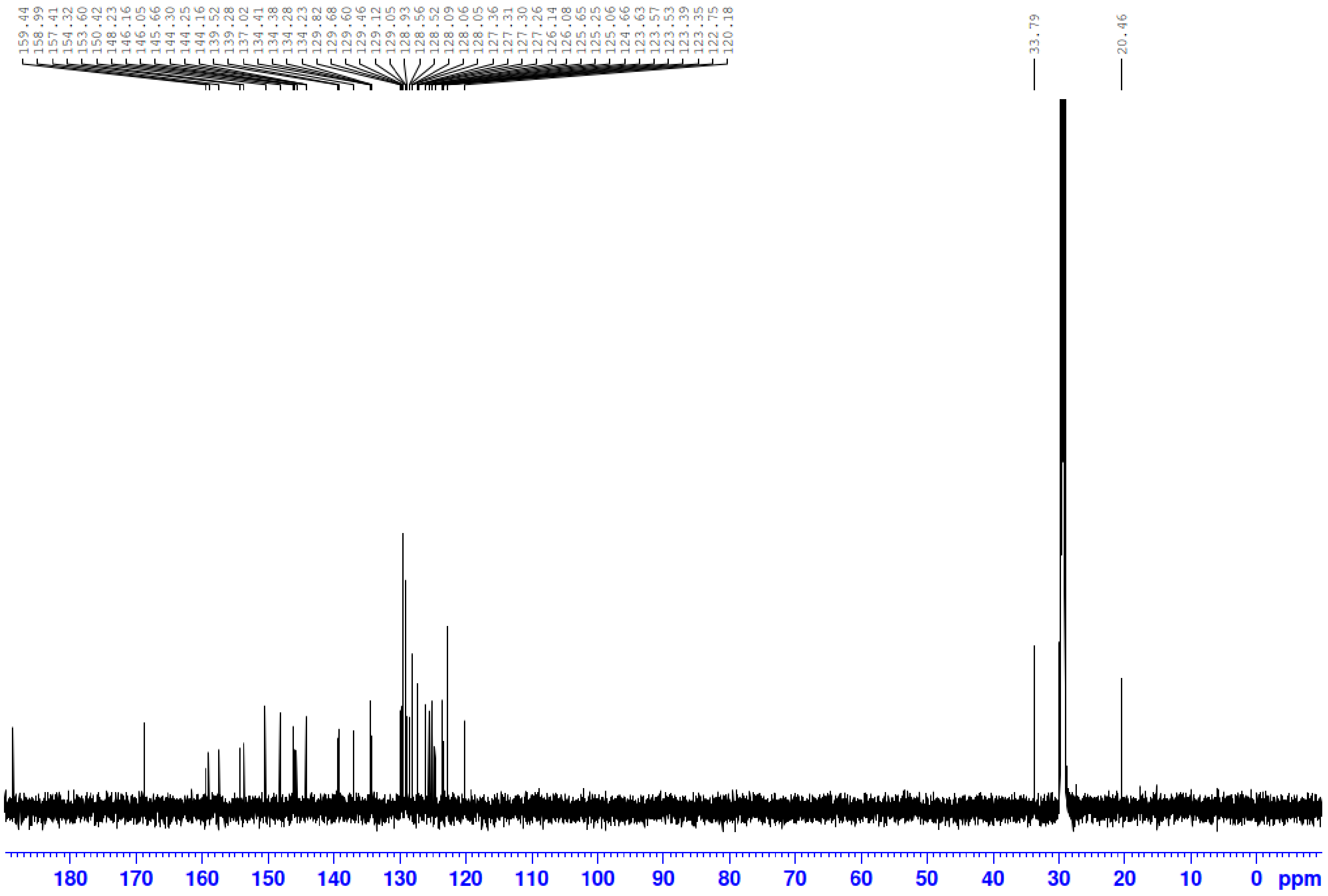

Figure S49. (a) Experimental and (b) simulated HR-ESI mass spectra of complex **4a** in CH_3CN.

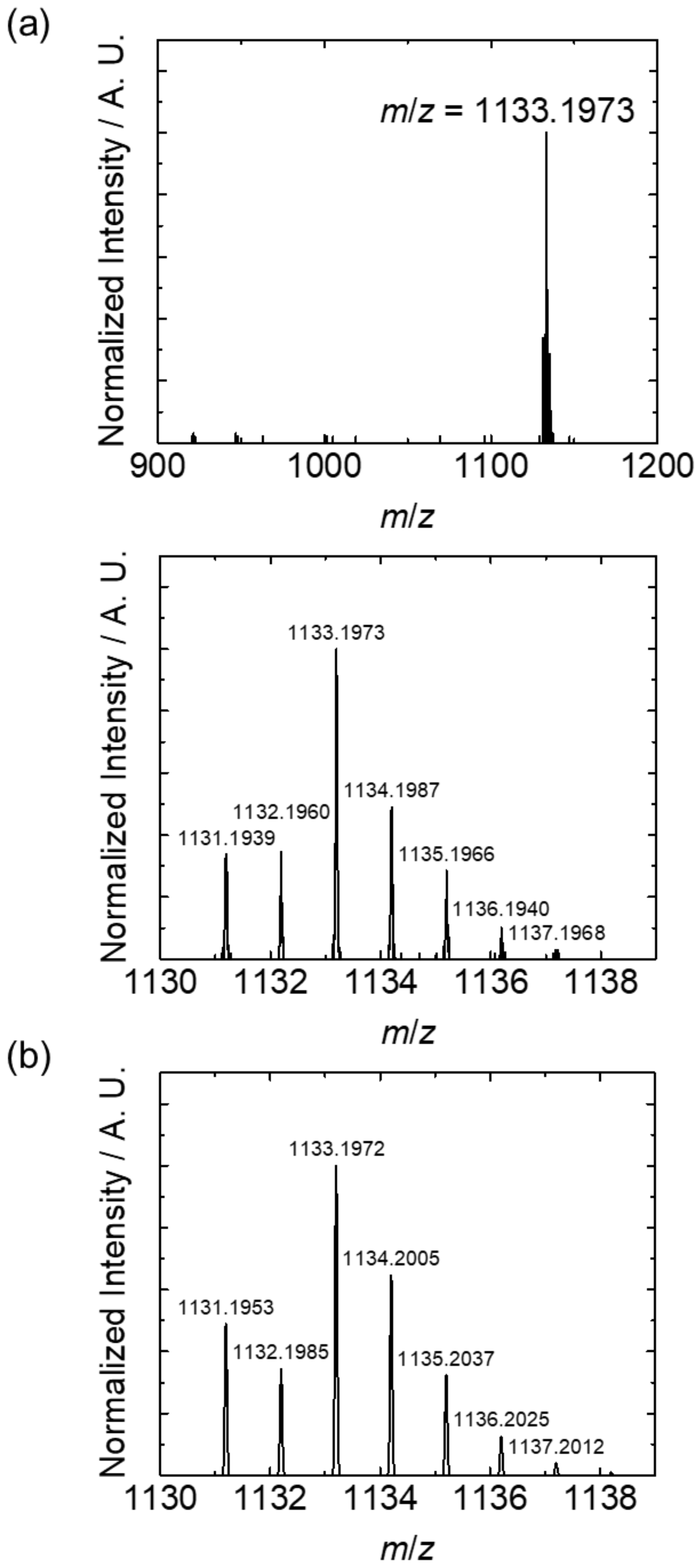

Figure S50. ^{1}H NMR spectrum of complex **4b** in $(CD_3)_2CO$ at 298 K.

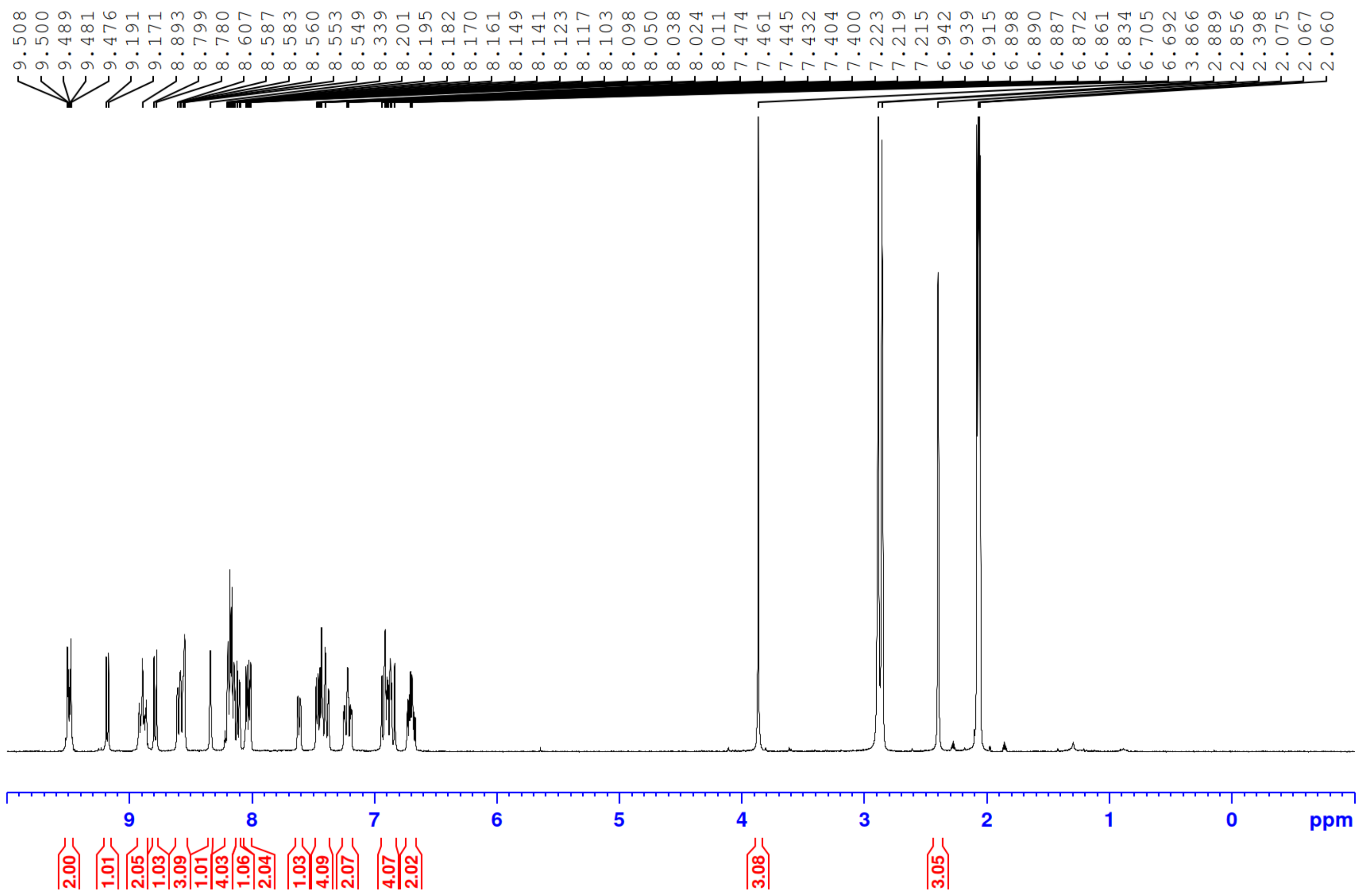

Figure S51. ^{13}C NMR spectrum of complex **4b** in $(CD_3)_2CO$ at 298 K.

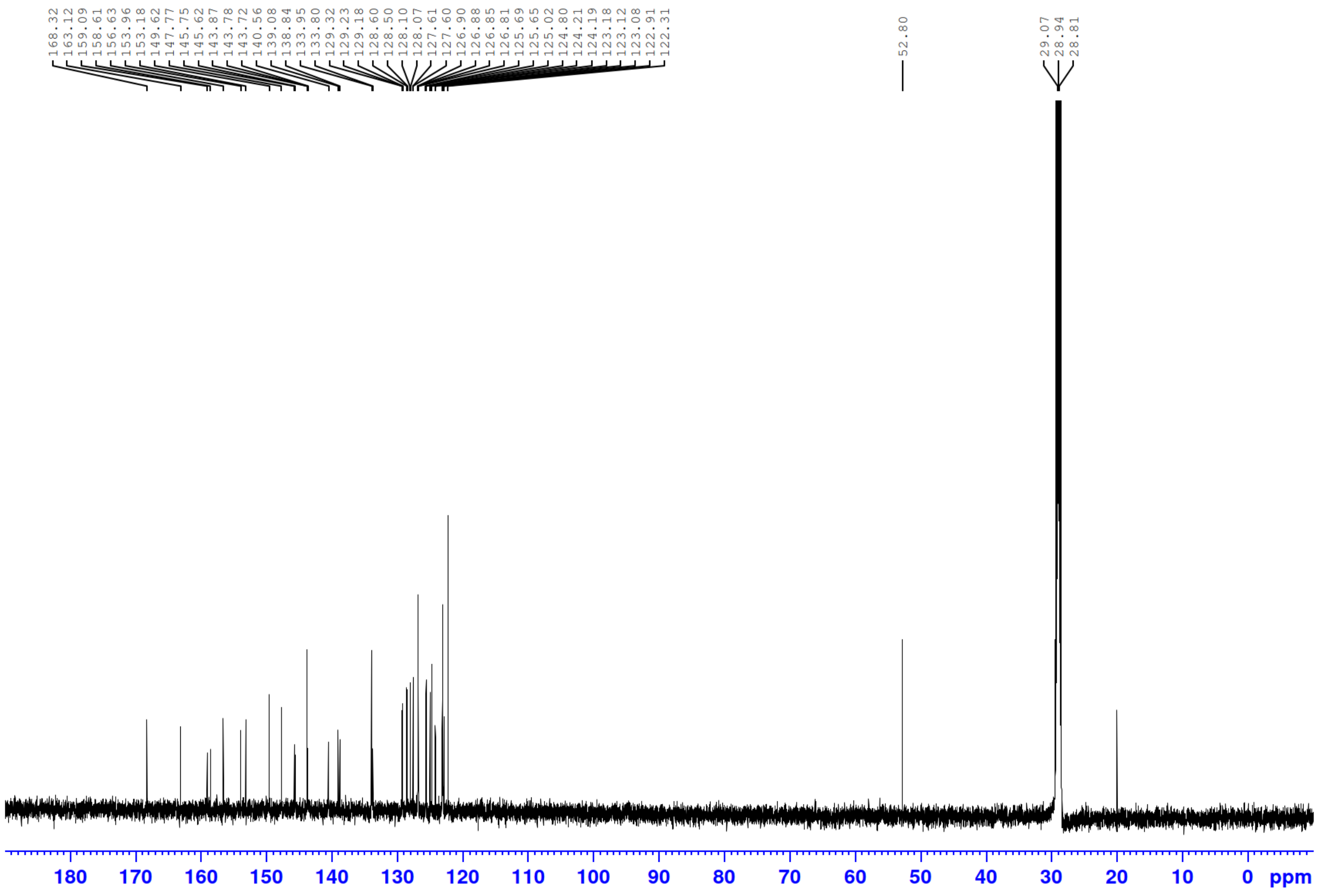

Figure S52. (a) Experimental and (b) simulated HR-ESI mass spectra of complex **4b** in CH_3CN.

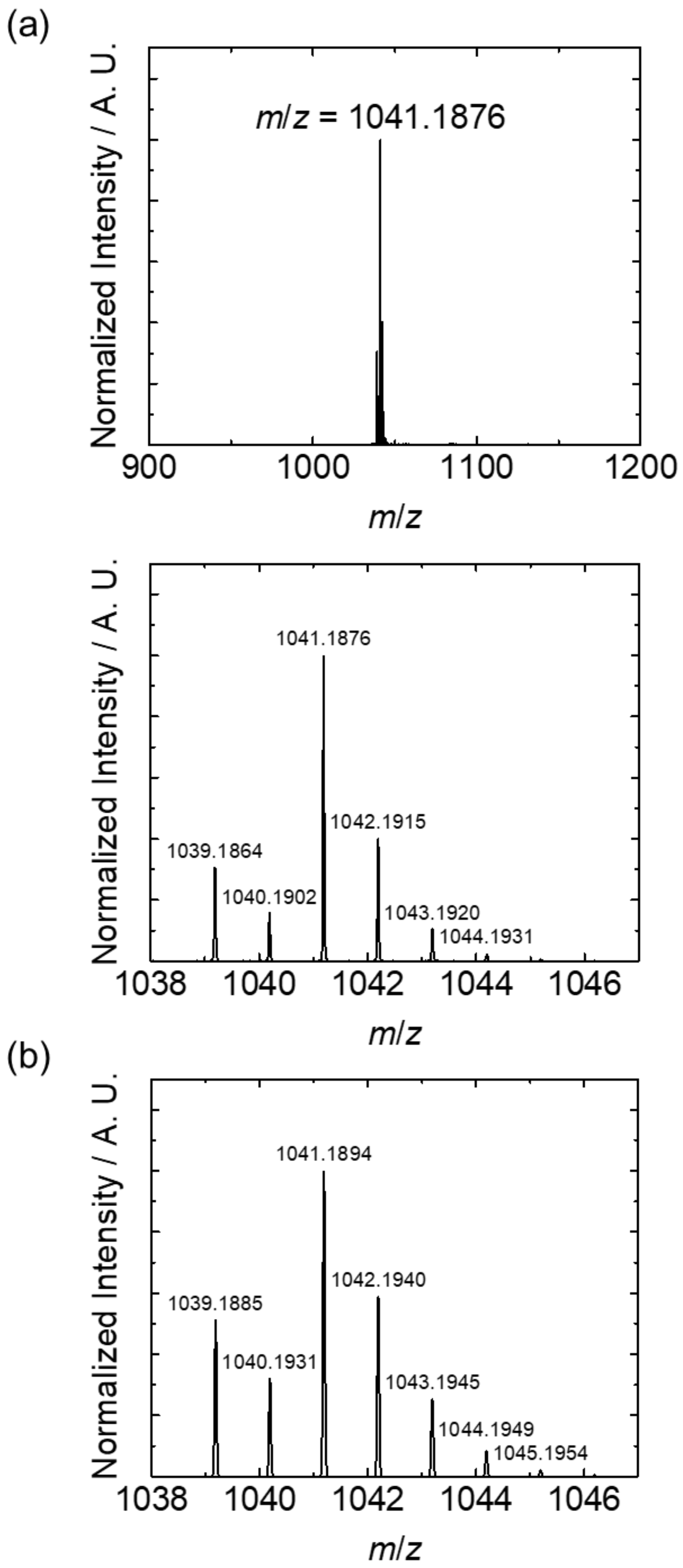

References

1. Armarego, W. L. F.; Chai, C. *Purification of Laboratory Chemicals (Seventh Edition)*. Butterworth-Heinemann: Oxford, **2013**.
2. Peek, B. M.; Ross, G. T.; Edwards, S. W.; Meyer, G. J.; Meyer, T. J.; Erickson, B. W. Synthesis of redox derivatives of lysine and related peptides containing phenothiazine or tris(2,2'-bipyridine)ruthenium(II). *Int. J. Pept. Protein Res.* **1991**, *38*, 114 – 123.
3. Wang, G.; Bergstrom, D. E. Controlled Oxidation of Dimethyl Derivatives of Pyridine, 2,2'-Bipyridine, and 1,10-Phenanthroline. *Synlett* **1992**, *1992*, 422 – 424.
4. Deligeorgiev, T. G. An Improved method for the preparation of 2-aryl-, 2-hetaryl- and 2-styrylbenzothiazoles. *Dyes Pigm.* **1990**, *12*, 243 – 248.
5. Kesarkar, S.; Mróz, W.; Penconi, M.; Pasini, M.; Destri, S.; Cazzaniga, M.; Ceresoli, D.; Mussini, P. R.; Baldoli, C.; Giovanella, U.; Bossi, A. Near-IR Emitting Iridium(III) Complexes with Heteroaromatic β-Diketonate Ancillary Ligands for Efficient Solution-Processed OLEDs: Structure–Property Correlations. *Angew. Chem. Int. Ed.* **2016**, *55*, 2714 – 2718.
6. Zheng, X.; Wang, X.; Mao, H.; Wu, W.; Liu, B.; Jiang, X. Hypoxia-specific ultrasensitive detection of tumours and cancer cells *in vivo*. *Nat. Commun.* **2015**, *6*, 5834.
7. (a) Sprouse, S.; King, K. A.; Spellane, P. J.; Watts, R. J. Photophysical effects of metal-carbon σ bonds in ortho-metalated complexes of iridium(III) and rhodium(III). *J. Am. Chem. Soc.* **1984**, *106*, 6647 – 6653. (b) Garces, F. O.; King, K. A.; Watts, R. J. Synthesis, structure, electrochemistry, and photophysics of methyl-substituted phenylpyridine ortho-metalated iridium(III) complexes. *Inorg. Chem.* **1988**, *27*, 3464 – 3471.

8. Demas, J. N.; Crosby, G. A. The Measurement of Photoluminescence Quantum Yields. A Review. *J. Phys. Chem.* **1971**, *75*, 991 – 1024.

9. Suzuki, K.; Kobayashi, A.; Kaneko, S.; Takehira, K.; Yoshihara, T.; Ishida, H.; Shiina, Y.; Oishi, S.; Tobita, S. Reevaluation of absolute luminescence quantum yields of standard solutions using a spectrometer with an integrating sphere and a back-thinned CCD detector. *Phys. Chem. Chem. Phys.* **2009**, *11*, 9850 – 9860.

10. Abdel-Shafi, A. A.; Beer, P. D.; Mortimer, R. J.; Wilkinson, F. Photosensitized Generation of Singlet Oxygen from Vinyl Linked Benzo-Crown-Ether−Bipyridyl Ruthenium(II) Complexes. *J. Phys. Chem. A* **2000**, *104*, 192 – 202.

11. Perdew, J. P.; Ernzerhof, M.; Burke, K. Rationale for mixing exact exchange with density functional approximations. *J. Chem. Phys.* **1996**, *105*, 9982 − 9985.

12. Adamo, C.; Barone, V. Toward reliable density functional methods without adjustable parameters: The PBE0 model. *J. Chem. Phys.* **1999**, *110*, 6158 − 6170.

13. Grimme, S.; Ehrlich, S.; Goerigk, L. Effect of the damping function in dispersion corrected density functional theory. *J. Comput. Chem.* **2011**, *32*, 1456 − 1465.

14. Andrae, D.; Häußermann, U.; Dolg, M.; Stoll, H.; Preuß, H. Energy-adjusted *ab initio* pseudopotentials for the second and third row transition elements. *Theor. Chim. Acta* **1990**, *77*, 123 − 141.

15. Scalmani, G.; Frisch, M. J. Continuous surface charge polarizable continuum models of solvation. I. General formalism. *J. Chem. Phys.* **2010**, *132*, 114110.

16. Frisch, M. J.; Trucks, G. W.; Schlegel, H. B.; Scuseria, G. E.; Robb, M. A.; Cheeseman, J. R.; Scalmani, G.; Barone, V.; Petersson, G. A.; Nakatsuji, H.; Li, X.; Caricato, M.; Marenich, A. V.; Bloino, J.; Janesko, B. G.; Gomperts, R.; Mennucci, B.; Hratchian, H. P.; Ortiz, J. V.; Izmaylov, A. F.; Sonnenberg, J. L.; Williams-Young, D.; Ding, F.; Lipparini, F.; Egidi, F.; Goings, J.; Peng, B.; Petrone, A.; Henderson, T.; Ranasinghe,

D.; Zakrzewski, V. G.; Gao, J.; Rega, N.; Zheng, G.; Liang, W.; Hada, M.; Ehara, M.; Toyota, K.; Fukuda, R.; Hasegawa, J.; Ishida, M.; Nakajima, T.; Honda, Y.; Kitao, O.; Nakai, H.; Vreven, T.; Throssell, K.; Montgomery, J. A., Jr.; Peralta, J. E.; Ogliaro, F.; Bearpark, M. J.; Heyd, J. J.; Brothers, E. N.; Kudin, K. N.; Staroverov, V. N.; Keith, T. A.; Kobayashi, R.; Normand, J.; Raghavachari, K.; Rendell, A. P.; Burant, J. C.; Iyengar, S. S.; Tomasi, J.; Cossi, M.; Millam, J. M.; Klene, M.; Adamo, C.; Cammi, R.; Ochterski, J. W.; Martin, R. L.; Morokuma, K.; Farkas, O.; Foresman, J. B.; Fox, D. J. *Gaussian 16, Revision A.03*. Gaussian, Inc.: Wallingford CT, **2016**.

17. Lu, T. sobMECP program; http://sobereva.com/286 (accessed November 31, 2024).

18. Runge, E.; Gross, E. K. U. Density-Functional Theory for Time-Dependent Systems. *Phys. Rev. Lett.* **1984**, *52*, 997 − 1000.

19. De Souza, B.; Farias, G.; Neese, F.; Izsák, R. Predicting Phosphorescence Rates of Light Organic Molecules Using Time-Dependent Density Functional Theory and the Path Integral Approach to Dynamics. *J. Chem. Theory Comput.* **2019**, *15*, 1896 − 1904.

20. Van Lenthe, E.; Snijders, J. G.; Baerends, E. J. The zero-order regular approximation for relativistic effects: The effect of spin-orbit coupling in closed shell molecules. *J. Chem. Phys.* **1996**, *105*, 6505 − 6516.

21. Pantazis, D. A.; Chen, X.-Y.; Landis, C. R.; Neese, F. All-Electron Scalar Relativistic Basis Sets for Third-Row Transition Metal Atoms. *J. Chem. Theory Comput.* **2008**, *4*, 908 − 919.

22. Weigend, F.; Ahlrichs, R. Balanced basis sets of split valence, triple zeta valence and quadruple zeta valence quality for H to Rn: Design and assessment of accuracy. *Phys. Chem. Chem. Phys.* **2005**, *7*, 3297 − 3305.

23. Neese, F.; Wennmohs, F.; Becker, U.; Riplinger, C. The ORCA quantum chemistry program package. *J. Chem. Phys.* **2020**, *152*, 224108.

24. Barone, V.; Cossi, M. Quantum Calculation of Molecular Energies and Energy Gradients in Solution by a Conductor Solvent Model. *J. Phys. Chem. A* **1998**, *102*, 1995 − 2001.

25. Cossi, M.; Rega, N.; Scalmani, G.; Barone, V. Energies, structures, and electronic properties of molecules in solution with the C-PCM solvation model. *J. Comput. Chem.* **2003**, *24*, 669 − 681.

26. Younker, J. M.; Dobbs, K. D. Correlating Experimental Photophysical Properties of Iridium(III) Complexes to Spin−Orbit Coupled TDDFT Predictions. *J. Phys. Chem. C* **2013**, *117*, 25714 – 25723.

27. Preiss, J.; Kage, D.; Hoffmann, K.; Martínez, T. J.; Resch-Genger, U.; Presselt, M. Ab Initio Prediction of Fluorescence Lifetimes Involving Solvent Environments by Means of COSMO and Vibrational Broadening. *J. Phys. Chem. A* **2018**, *122*, 9813 − 9820.

28. Strickler, S. J.; Berg, R. A. Relationship between Absorption Intensity and Fluorescence Lifetime of Molecules. *J. Chem. Phys.* **1962**, *37*, 814–822.

29. López-Estrada, O.; Laguna, H. G.; Barrueta-Flores, C.; Amador-Bedolla, C. Reassessment of the Four-Point Approach to the Electron-Transfer Marcus–Hush Theory. *ACS Omega* **2018**, *3*, 2130 − 2140.

30. Beljonne, D.; Shuai, Z.; Pourtois, G.; Bredas, J. L. Spin−Orbit Coupling and Intersystem Crossing in Conjugated Polymers: A Configuration Interaction Description. *J. Phys. Chem. A* **2001**, *105*, 3899 − 3907.

31. Samanta, P. K.; Kim, D.; Coropceanu, V.; Brédas, J.-L. Up-Conversion Intersystem Crossing Rates in Organic Emitters for Thermally Activated Delayed Fluorescence: Impact of the Nature of Singlet vs Triplet Excited States. *J. Am. Chem. Soc.* **2017**, *139*, 4042 − 4051.
